# An Unexpected Isomerization for the Total Synthesis of Daphnepapytone A

**DOI:** 10.1021/acs.orglett.6c00312

**Published:** 2026-03-02

**Authors:** Ilja Lubins, Bernhard Breit

**Affiliations:** Institut für Organische Chemie, 9174 Albert-Ludwigs-Universität Freiburg, Albertstraße 21, 79104 Freiburg i. Bg., Germany

## Abstract

We report a high-yielding total synthesis of sesquiterpenoid daphnepapytone A from (*S*)-glycidol. The groundwork for the synthesis is laid via reductive cleavage of a bicyclic Pauson–Khand-derived cyclopentenone ether, giving efficient access to a trisubstituted chiral cyclopentenone which is difficult to obtain by other means. To introduce the cyclobutane motif, the enone was irradiated in the presence of allene gas, yielding the unwanted exo-cyclobutane at worst and 1:1 mixtures of the endo- and exo-product at best. A serendipitous epimerization of an aldehyde in the following steps converted the unwanted exo-epimer into the endoenantiomer, which significantly improved the final yield. The final cage was connected with a second Pauson–Khand reaction, requiring only one more step to yield the natural product.

## Graphical abstract

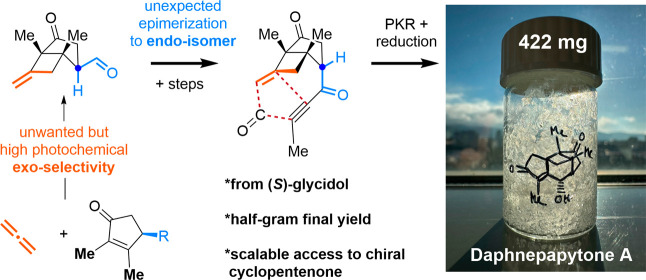

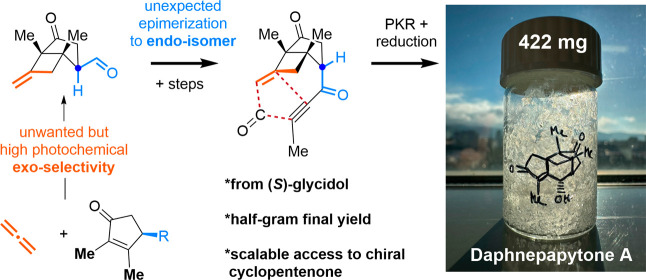

Daphnepapytone A is an unusual sesquiterpenoid that was first isolated from the dried stems of *Daphne papyracea*,^1^ an evergreen shrub from East Asia found at altitudes between 700 and 3100 m. Extracts of this shrub have been shown to possess inhibitory activity against a protease of the Hepatitis C virus,^2^ and daphnepapytone A specifically was found to have moderate α-glycosidase inhibitory activity (IC_50_ = 159 μM).^1^ Only three years after its first isolation, its intriguing structure featuring a cyclopentenone-annelated tricyclo­[4.3.0.0^3,9^]­nonane cage has attracted the attention of organic chemists, leading to four published total syntheses (Scheme 1).^3–6^ All four routes are ex-chiral pool syntheses from (*R*)-carvone as the starting material, bringing with it the isopropenyl group with the correct stereoinformation. Cleavage of carvone via Eschenmoser–Tanabe fragmentation gave a precursor **1** that was converted into allene-yne **3** via an alcohol analogue thereof. Nay’s, Li/She’s, and Hanson’s syntheses follow a biomimetic approach, converting the allene-yne using a rhodium-catalyzed Pauson–Khand reaction (PKR) to access oleodaphnone^7^ (**5**), a guaiane type sesquiterpene, as a photochemical precursor and proposed intermediate in the biosynthesis of daphnepapytone A.^1^ Indeed, the intramolecular photochemical [2 + 2]-cycloaddition proceeds effectively, yielding the tetracyclic precursor **6** of daphnepapytone A which, following a CH-oxidation adjacent to the enone, is selectively reduced to the target compound. Stoltz followed a different approach to establish the central cyclobutane. In an unusual thermal allenone-ene-[2 + 2]-cycloaddition,^8^ the cyclobutane is established, albeit with low selectivity toward the desired endoproduct **4**. A subsequent Co_2_CO_8_-mediated PKR yields the tetracyclic cage **6** of daphnepapytone A, and the final compound is acquired in a similar oxidation–reduction sequence.^3^

**Scheme 1:**
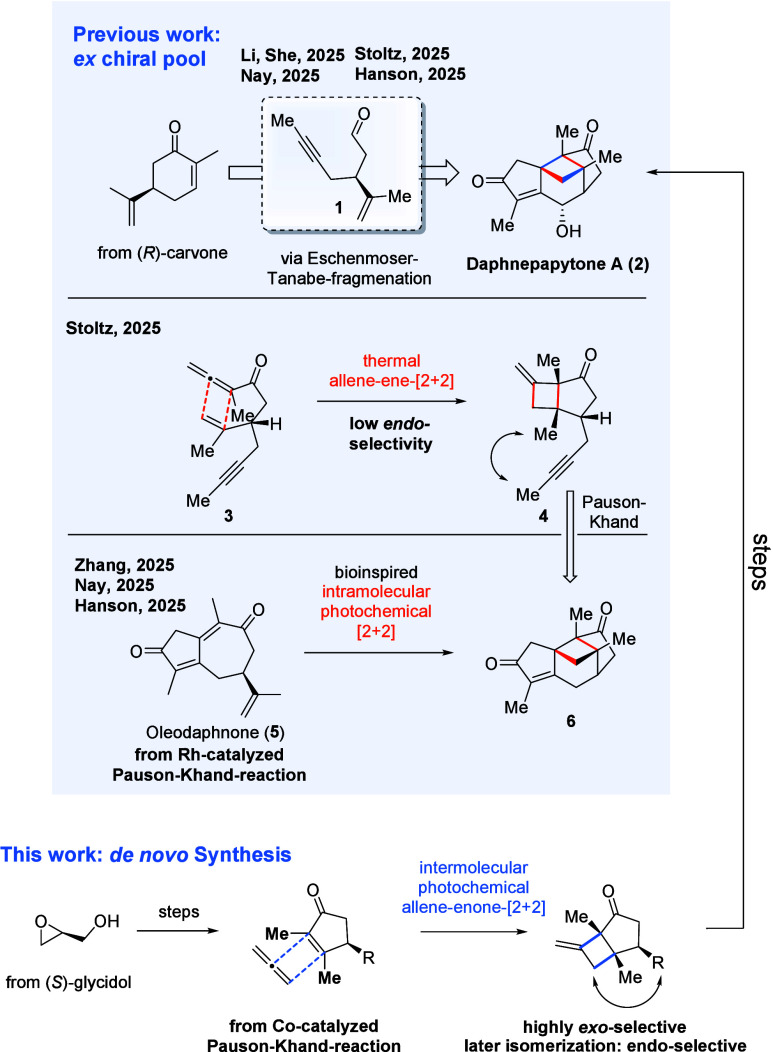
Comparison of Previous Syntheses and Our Approach Towards the Total Synthesis of Daphnepapytone A

In our synthesis plan (Scheme 2i), we envisioned omitting the CH-oxidation of **6** featured in the previously published routes and directly using a propargylic alcohol **7** in a Pauson–Khand reaction to access the final compound. If the natural selectivity of propynyl-organometallic reagent cannot be tuned by other means, the propargylic alcohol could potentially be accessed via an asymmetric alkynation of an aldehyde.^9–11^ The aldehyde could be accessed from usually high yielding diol cleavage of **8**. The cyclobutane featuring the methylidene group would be introduced in photochemical enone-allene-[2 + 2]-cycloaddition with head-to-head selectivity from precursor **9** accessed via the reductive cleavage of PKR-derived bicyclic ether **10**. While asymmetric rhodium-catalyzed PKR is well-established and would not require to use a chiral enyne, reported catalyst loadings are typically >5 mol % ^12,13^ and therefore it is expensive to access gram amounts of the compound. For that reason, we envisioned performing a diastereoselective cobalt-catalyzed PKR^14^ of the easily accessible chiral enyne **11** that can be synthesized via attack of a sulfur-ylide^15^ on protected (*S*)-glycidol and subsequent etherification. Most doubt for success was shown toward the desired *endo*-stereoselectivity of the intermolecular photochemical step (Scheme 2ii), since no literature precedent exists for [2 + 2]-cycloadditions for the substitution pattern of 2,3,4- yet alone 3,4-substituted monocyclic cyclopentenones, e.g., **15**. While bicyclic enones (**12**) have clear (but sometimes counterintuitive) stereochemical outcomes^16–19^ that can be empirically rationalized using a rule proposed by Wiesner,^20^ data on monocyclic enones is sparse^21,22^ and—for the known examples—not in favor of our synthetic plan. For example, an intermediate for the total synthesis of quadrone^18^ was synthesized in over 80% yield via irradiation of a bicyclic enone, selectively furnishing the *exo*-tricycle **13**. On the other hand, OTBS-substituted cyclopentenone yielded roughly 2:1 mixtures of *exo:endo*-products **14**, without room for much tunability via solvent or substitution.^21^ Unbothered by these odds and motivated by scientific curiosity, we deemed that applying Wiesner’s empirically rationalized model to our enone-system (**15**) would favor the *endo*-product due to higher interaction of the methyl-group at the assumed excited state’s (**16**) β-carbon with the residue R, an assumption that proved incorrect.

**Scheme 2:**
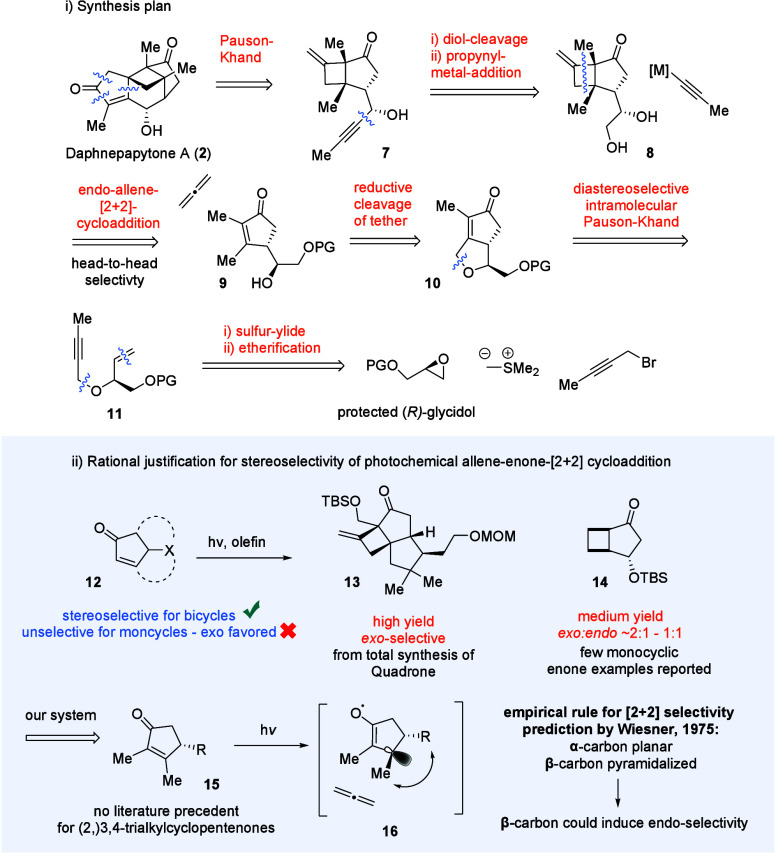
^a^

a (i) Initial synthesis plan of daphnepapytone A. (ii) Literature on the stereochemistry of [2 + 2]-photochemical cycloadditions of cyclopentenones and empirical reasoning for potential *endo*-selectivity in our synthetic plan.

The synthesis (Scheme 3, white) began with protection of (*R*)-glycidol with PMBCl and subsequent sulfur-ylide attack to convert it to known chiral allylic alcohol **18**,^15,23^ which was transformed into its corresponding propargylic ether **19**. Initial attempts with TBDPS-protected glycidol suffered from diminished yields, presumably from silyl migration^24,25^ after epoxide ring opening. The chiral enyne **19** was subjected to a catalytic PKR with only 5 mol % cobalt octacarbonyl and 20 mol % dimethoxyethane as an additive^26^ under 30 bar of CO pressure. Cyclopentenone **20** was generated in high yield and diastereoselectivity, and the minor isomer was separated via chromatography. To access the free methyl group of **21a**, the tether was cleaved quantitatively after 10 min in a suspension of zinc in glacial acetic acid.^27,28^ While the sequence of a Pauson–Khand reaction with concomitant reductive cleavage of the ethereal tether is reported,^29,30^ our sequence using zinc circumvents the need for stoichiometric use of Co_2_CO_8._ Then, a thorough photochemical screening (see Supporting Information) with different protection (**21a**–**c**) at the vicinal diol followed, and no selectivity toward the desired *endo*-product could be achieved with solvent variation. At best, a ∼1:1 mixture of *exo-* and *endo-*products was formed at −78 °C in THF for deprotected diol substrate **21b**. Higher temperatures converged toward 1:1 *exo*–*endo* mixtures with many inseparable side products. Increasing polarity while maintaining a low freezing point (e.g., THF/DMF 1:1) favored *exo*-product formation. The slightly greater steric bulk of substrate **21a** further diminished the ratio of the wanted *endo*-bicycle (THF, 2:1). With even greater bulkiness introduced by TBS protection at the secondary alcohol (**21c**), the cycloaddition proceeded toward the unwanted *exo*-product **22c** with high yield and selectivity. Capitulating on a mediocre *endo*-yield with substrate **21b**, the synthesis proceeded with diol-mixture **22/23b**, which was cleaved by using NaIO_4_. Luckily, the *exo*–*endo* mixture **24a/b** of the resulting aldehyde was separable via a lengthy chromatography, during which an epimerization was noticed that partially converted the *exo*-epimer **24a** into a mixture of the latter with the *endo*-enantiomer **ent-24b** so that full isomerization of this mixture would lead to a racemic compound **rac-24b**. This prompted us to investigate epimerization conditions, which were shown to be most effective in an NMR-tube with a small amount of basic alumina at the bottom, generating a 11:2 *exo*–*endo* mixture at equilibrium. The final relative stability of the isomeric aldehydes was qualitatively confirmed by DFT calculations, which assigned a ∼2 kcal difference in favor of the *endo*-isomer that was not quite met in reality. Since the [2 + 2]-cycloaddition toward the previously unwanted TBS-protected *exo*-isomer **22c** proceeded selectively and with high yield, a new route (Scheme 3, blue shade) was devised starting from (*S*)-glycidol; this time, however, TBS-protected alcohol **ent-21c** was used in the photochemical step. This gave 77% (90% *brsm*) yield of >90% pure *exo*-photoadduct **ent-22c**, which was deprotected to yield the first solid compound **ent-22b** of this synthesis, confirming the structure via X-ray crystallography. After diol cleavage with periodate, epimerization of **ent-24a** at 1.5 g scale proceeded significantly more slowly than the NMR-scale screening, which was fixed by stirring the mixture, accelerating the isomerization from 5 days to overnight. Aldehyde **24b** was reacted with propynyllithium to yield an inseparable diastereomeric 2:1 mixture of propargylic alcohols. Subjecting this mixture to Pauson–Khand conditions did not proceed cleanly, so the alcohols were oxidized using Dess-Martin periodinane to yield ynone **25** which, after preformation to cobalt-complex **26**, underwent carbocyclization with yields ranging from 52% to 83% for different scales. For a similar step using substrate **4** (Scheme 1), Stoltz reports only a low yield due to a novel isomerization. Finally, reduction with NaBH_4_ at −78 °C in THF/MeOH furnished 422 mg of (*+*)-daphnepapytone A (**2**), yielding 2 orders of magnitude more substance than all previously reported procedures.^3–6^

**Scheme 3:**
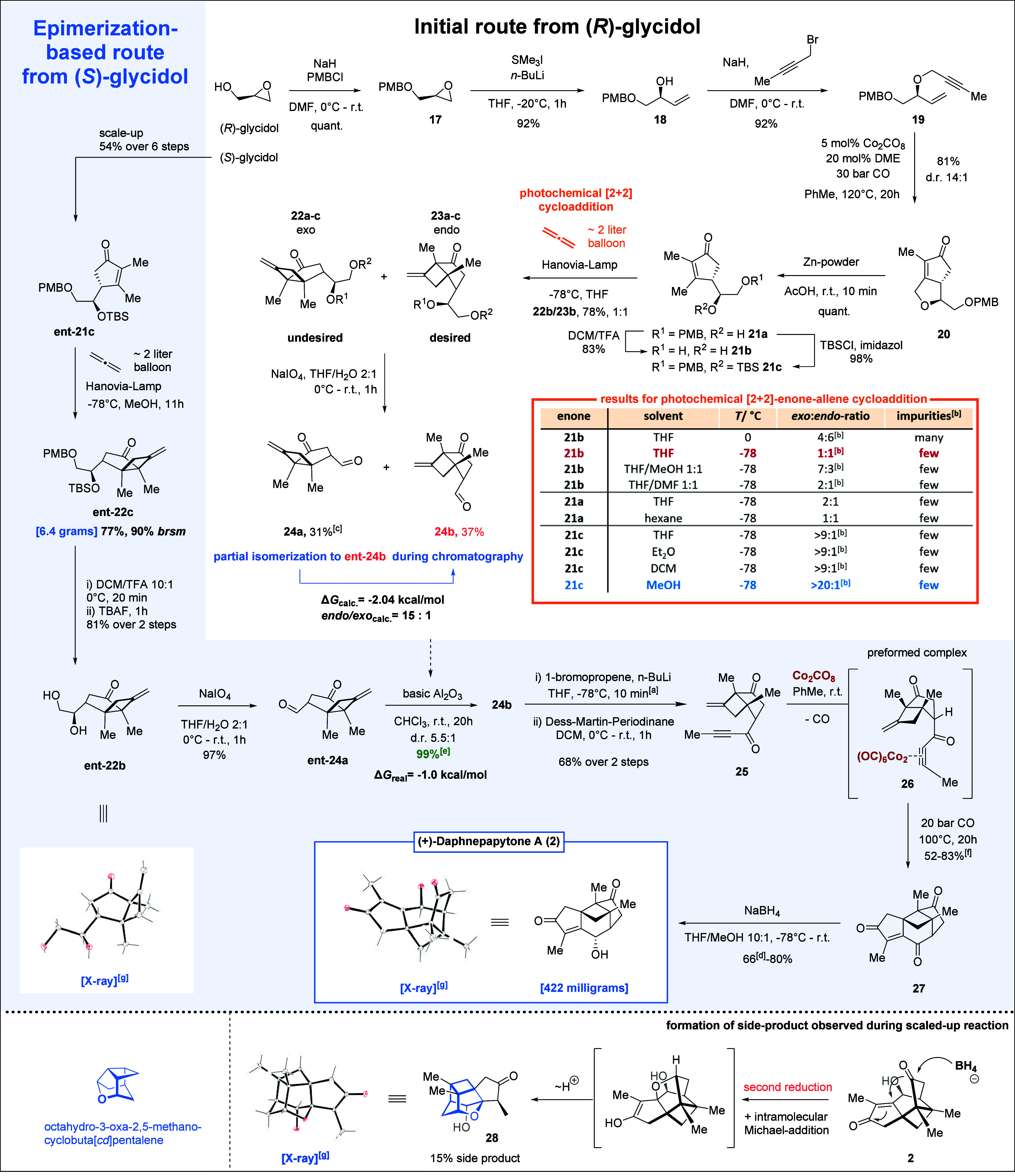
Total Synthesis of (+)-Daphnepapytone A from (*S*)-Glycidol

a Inseparable mixture, d.r. 2:1
b Inseparable via column chromatography.
c Two fractions with d.r. 7:1 and 3:1.
d +15% of side product **28** for the scaled up reaction, see bottom of Scheme 3.
e 83% of **24b** isolated.
f Average yield of 65%.
g 50% ellipsoid contour probability.

Interestingly, for the larger scale reaction, an over-reduced product **28** was isolated in 15% yield, which was previously not observed for a smaller scale. As visualized on structure **2**, borohydride can attack the ketone from the convex side of the cage, bringing the resulting alcoholate in spatial proximity to the enone’s *C*3 and forcing the intramolecular Michael-addition. This results in the formation of yet another cage more complex than before, which can be described as a cyclopentenone-annelated octahydro-3-oxa-1*H*-2,5-methanocyclobuta-[*cd*]-pentalene, a motif not yet encountered in nature. Brief attempts to access **28** selectively via direct reduction of **2** have not yet been successful.

In summary, we achieved the *de novo* total synthesis of daphnepapytone A (**2**) through a sequence that features (1) efficient and convenient access to a chiral trisubstitued cyclopentenone *via* reductive cleavage of a Pauson–Khand-derived ether; (2) novel insights into the stereochemistry of photochemical [2 + 2]-cycloaddition to monocyclic enones, achieving high *exo-* selectivity through steric bulk; (3) serendipitous circumvention of *exo*-selectivity with mild and selective epimerization of the *exo*-aldehyde **ent-24a** to the *endo*-aldehyde **24b**; and (4) a congested Pauson–Khand reaction of ynone **27** that generated a quaternary carbon at a cyclobutane with good yield. Since no data were available on the stereochemistry of [*2 + 2*] photocycloaddition to monocyclic cyclopentenones, we deem that illuminating this blind spot is of great use for future synthetic chemists. And last, being able to selectively access both *endo*- and *exo*-substituted bicyclo­[*3.2.0*]­heptanes (e.g., **24a/b**) *via* one sequence could potentially open more efficient routes to other natural products.^31,32^

## Supplementary Material



## Data Availability

The data underlying this study are available in the published article and its Supporting Information.

## Notes

Experimental details, optimization of the photochemical reaction and characterization data, X-ray crystallographic data, NMR spectra and computational details (PDF)
