# Supplementary material for: An Unexpected Isomerization for the Total Synthesis of Daphnepapytone A
Source: Org Lett. 2026 Mar 2;28(10):3255–9. doi: 10.1021/acs.orglett.6c00312 (PMC12993916; doi:10.1021/acs.orglett.6c00312)
Supplement: Supplementary file 1 [file ol6c00312_si_001.pdf]

An Unexpected Isomerization for the Total Synthesis of Daphnepapytone A

Supporting Information

Ilja Lubins and Bernhard Breit*

Albert-Ludwigs-Universität Freiburg, Albertstraße 21, 79104 Freiburg im Breisgau, Germany

E-mail: bernhard.breit@chemie.uni-freiburg.de

Table of contents

1. General methods and materials 2

2. Photochemical screening for enone-allene [2+2] cycloadditions 4

3. Experimental procedures 5

3.1 Initial route from (*R*)-glycidol 5

3.2 Isomerization-based route from (*S*)-glycidol 14

4 NMR-spectra 23

5 Crystal structure data 39

6 DFT-calculations on relative stability of 24a/ent-24b (exo/endo) 56

7 References 58

1. General methods and materials

General information

Commercial reagents were used without further purification. Air- and moisture-sensitive liquids were transferred by syringe flushed three times with Argon. Reactions involving air- and moisture-sensitive reagents were performed using Schlenk techniques in heat-gun dried and argon-flushed glassware. Evaporation of solvent under reduced pressure was performed by rotary evaporation at 40 °C on a *Heidolph Laborata 4001-efficient*.

Chromatography

FCC (Flash Column Chromatography) was carried out using MACHEREY-NAGEL silica gel 60 ® (230 - 400 mesh). TLC (Thin Layer Chromatography) was performed on aluminum plates pre-coated with silica gel (MERCK, 60F254), which were visualized by UV fluorescence (λ_{max} = 254 nm) and/or by staining with 1% w/v $KMnO_4$ in 0.5 M aqueous K_2CO_3.

NMR (Nuclear Magnetic Resonance)

Spectra were recorded at room temperature. The NMR measurements were performed on a VARIAN Mercury 300 spectrometer (^{1}H [300 MHz], ^{13}C [75.5 MHz]) and on the BRUKER devices Avance 400 (^{1}H [400 MHz], ^{13}C [100 MHz]) and Avance 500 (^{1}H [500 MHz], ^{13}C [125 MHz]). Chemical shifts are reported in parts per million (ppm) from the residual solvent peak. Chemical shifts (δ) are given in ppm and coupling constants (J) are quoted in hertz (Hz). Resonances are described as s (singlet), d (doublet), t (triplet), q (quartet) and m (multiplet). MestReNova was used forevaluation of all spectra.

HR-MS (High resolution mass)

Spectra were obtained on a THERMO SCIENTIFIC Exactive instrument (Orbitrap) and a Agilent 6545 LC/Q-TOF (APCI/MeOH: spray voltage 4-5 kV, ion transfer tube: 250 - 300°C, vaporizer: 300 – 400°C)

Optical rotation

Optical rotation of chiral compounds was determined on a A. KRÜSS OPTRONIC P8000-Tapparatus and transformed to the specific optical rotation for a given temperature according to the following formula:

$$[\alpha]_D^T = \frac{\alpha \times 100}{c \times d}$$

α = measured value for optical rotation [°] *c* = concentration [g/100 ml]

d = length of cuvette [dm] *T* = temperature [°C]

Carbon monoxide pressure reactions

Pauson-Khand-reactions were carried out under CO-pressure (supplier Air Liquide). The autoclaves were pressurized from a gas manifold equipped with precautionary sensors. The stainless-steel autoclaves of varying volumes were manufactured by the workshop of the institute. Heating of the reaction was achieved by an aluminium heating block standing on a magnetic stirrer.

Irradiation experiments

Irradiation was carried out using 150 W Hanovia TQ 150 high pressure mercury UV-lamps. If the lamps were not immersed in dry ice/acetone, active cooling was carried out with water in a quartz cooling mantle.

Hanau-Hochdrucklampe TQ 150

Wellenlänge in nm	Hg	Hg/Z1	Hg/Z2	Hg/Z3
250 – 300	6,7	8,1	8,3	3,6
300 – 350	7,9	3,7	3,4	7,0
350 – 400	8,0	8,2	14,2	7,9
400 – 450	8,3	18,5	3,2	4,5
450 – 500	–	–	–	4,6
500 – 550	7,2	3,3	13,8	5,9
550 – 600	7,0	2,4	0,8	1,3
600 – 650	–	–	–	3,8
650 – 700	–	–	–	–
700 – 750	–	–	–	6,4

Hanau-Nie

We
248
254
265
276
280
289
297
302
Faktor zur Strahlungsf

Manufacturer supplied data sheet for emission spectrum for the Hanovia TQ 150 high pressure mercury lamp.

2. Photochemical screening for enone-allene [2+2] cycloadditions

The screening was performed according to the following general procedure:

To an NMR-tube with a septum screw cap equipped with an Ar-balloon, 20-25 µmol of enone dissolved in degassed solvent (2 mL), that has been previously saturated with propadiene (~1 M) were transferred. The tube was immersed into an acetone-dry ice bath next to a Hanovia mercury UV-lamp with a pyrex filter, such that the NMR-tube touches the walls of the filter. Irradiation was carried out for 30 – 60 minutes. If the solvent was not deuterated, the solvent was removed under reduced pressure and dissolved in deutrated solvent for NMR analysis.

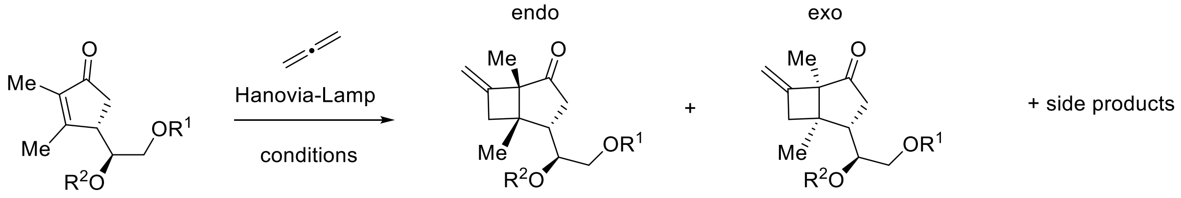

21b, R^1 = H, R^2 = H
21a, R^1 = PMB, R^2 = H
21c, R^1 = PMB, R^2 = TBS

23b, R^1 = H, R^2 = H
23a, R^1 = PMB, R^2 = H
23c, R^1 = PMB, R^2 = TBS

22b, R^1 = H, R^2 = H
22a, R^1 = PMB, R^2 = H
22c, R^1 = PMB, R^2 = TBS

enone	solvent	temperature/ °C	Time/ h	conversion	endo:exo-ratio	Unidentified Impurities*
**21b**	THF	0	1	>95	4:6*	many
**21b**	THF	-78	1	85	1:1*	few
**21b**	THF/MeOH 1:1	-78	1	>95	7:3*	few
**21b**	THF/DMF 1:1	-78	1	>95	2:1*	few
**21a**	THF	-78	1	>95	2:1	few
**21a**	hexane	-78	1	25	1:1	few
**21c**	THF	-78	0.5	67	>9:1*	few
**21c**	Et_2O	-78	0.5	20	>9:1*	few
**21c**	DCM	-78	0.5	13	>9:1*	few
**21c**	**MeOH**	**-78**	**0.5**	**54**	**>20:1***	**few**

*inseparable via chromatography

Notes:

Many experiments were performed at higher temperatures (-40°C, 0°C, r.t. and higher). The general trend was that deviation from -78°C in any solvent significantly increased unidentified inseparable impurities and the exo:endo ratio tended towards 1:1. Irradiation with an Ultravitalux sun-lamp showed similar results.

3. Experimental procedures

3.1 Initial route from (*R*)-glycidol

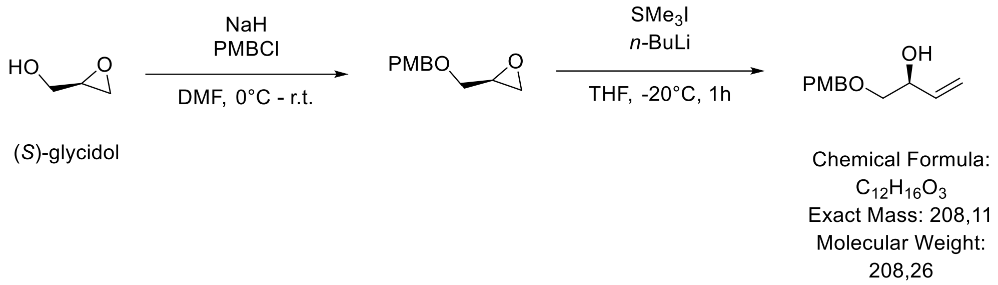

(*S*)-1-((4-methoxybenzyl)oxy)but-3-en-2-ol (18)

Benzylic ether[1] and allylic alcohol[2] were synthesized according to known procedures in different scales.

In a heat-gun dried three-necked round-bottom flask equipped with a stir bar NaH (3.0 g, 74.5 mmol, 60% in paraffin oil, 1.1 eq.) was washed 3 times with hexane, filled with DMF (130 mL) and cooled to 0°C. PMBCl (11.6 g, 74.5 mmol, 1.1 eq.) was added dropwise and stirred for 20 minutes. Then, glycidol (5.0 g, 67.5 mmol) was added dropwise under visible hydrogen evolution. The reaction was left to warm to room temperature and stirred overnight. The reaction was quenched with sat. NH_4Cl solution (~10 mL), water (200 mL) and extracted with EtOAc (300 mL). The organic phase was dried with Na_2SO_4 and the solvent was removed under reduced pressure. Brief column chromatography (pentane/ether 5:1) yielded the epoxy-ether **17** in quantitative yield (13.1g, 67.5 mmol) and used in the next step.

To a solution of SMe_3I (37 g, 3.0 eq.) in dry THF (500 mL) under argon, *n*-BuLi (64 mL, 2.5 M in hexanes, 2.7 eq.) was added dropwise at -10°C. The mixture was stirred at this temperature for 30 minutes before PMB-glycidol **17** (11.60 g, 59.7 mmol) in 50 mL of THF was added dropwise. The mixture was stirred at r.t. for 2 h. The reaction was quenched with water (500 mL) and extracted with Et_2O (500 mL). The organic phase was dried with Na_2SO_4 and the solvent removed under reduced pressure. The crude product was purified via short column chromatography (silica gel, pentane/EtOAc – 2:1) to yield **18** as a clear beige oil (11.46 g, 55.03 mmol, 92%).

The analytic data matched reported literature.[1,2]

(*S*)-1-(((2-(but-2-yn-1-yloxy)but-3-en-1-yl)oxy)methyl)-4-methoxybenzene (19)

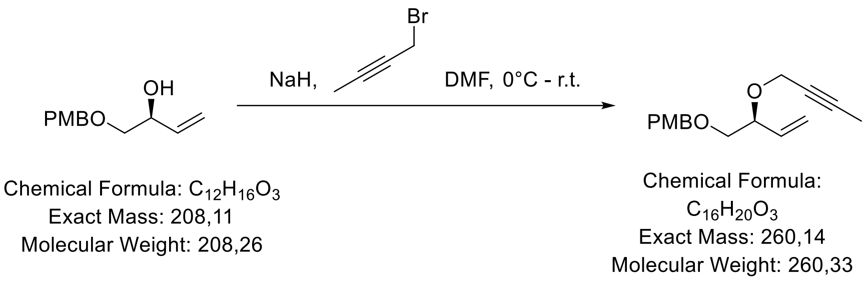

In a heat-gun dried three-necked flask under argon equipped with a stir bar, NaH (2.64 g, 66.0 mmol, 60% in paraffin oil) was washed three times with hexane and suspended in dry DMF (120 mL), cooled to 0°C and allylic alcohol **18** (11.46 g, 55.0 mmol) was added dropwise. The reaction was slowly warmed to r.t. under visible hydrogen evolution. Once H_2-bubbling stopped (~30 min), 1-butynylbromide (10.93 g, 7.2 mL, 1.5 eq.) was added and the reaction was stirred for 2 h. The reaction was quenched with sat. NH_4Cl-solution and diluted with water (100 mL). The mixture was extracted with Et_2O (200 mL), the organic phases was dried with Na_2SO_4 and the solvent removed under reduced pressure. The crude mixture was purified via column chromatography (silica gel, pentane/EtOAc 10:1) to yield the title compound **19** (13.16 g, 50.6 mmol, 92%) as a clear yellowish oil.

^{1}H NMR (500 MHz, $CDCl_3$) δ = 7.30 – 7.23 (m, 2H), 6.90 – 6.84 (m, 2H), 5.78 – 5.68 (ddd, *J* = 17.3, 10.3, 7.4 Hz, 1H), 5.33 (ddd, *J* = 17.3, 1.7, 1.0 Hz, 1H), 5.28 (ddd, *J* = 10.3, 1.7, 0.9 Hz, 1H), 4.57 – 4.44 (m, 2H), 4.20 (dq, *J* = 15.1, 2.3 Hz, 1H), 4.13 (dddt, *J* = 7.4, 6.3, 4.6, 1.0 Hz, 1H), 4.07 (dq, *J* = 15.1, 2.3 Hz, 1H), 3.80 (s, 3H), 3.54 (dd, *J* = 10.3, 6.3 Hz, 1H), 3.48 (dd, *J* = 10.3, 4.6 Hz, 1H), 1.84 (t, *J* = 2.3 Hz, 3H).

^{13}C NMR (126 MHz, $CDCl_3$) δ = 159.3, 135.3, 130.4, 129.5, 119.0, 113.9, 82.3, 78.8, 75.4, 73.1, 72.5, 56.5, 55.4, 3.7.

HR-MS (APCI+): m/z calc. for $C_{16}H_{20}O_3^+$ [M^+]: 260.1407, found 260.1406.

$[a]^D_{25.6}$ = +45.2° ($CHCl_3$, c = 1.0). **$[a]^D_{22.0}$** (**ent-19**) = - 46.0° ($CHCl_3$, c = 1.0).

(3*S*,3a*S*)-3-(((4-methoxybenzyl)oxy)methyl)-6-methyl-3a,4-dihydro-1H-cyclopenta[c]furan-5(3H)-one (major) (20)

(3*S*,3a*R*)-3-(((4-methoxybenzyl)oxy)methyl)-6-methyl-3a,4-dihydro-1H-cyclopenta[c]furan-5(3H)-one (minor)

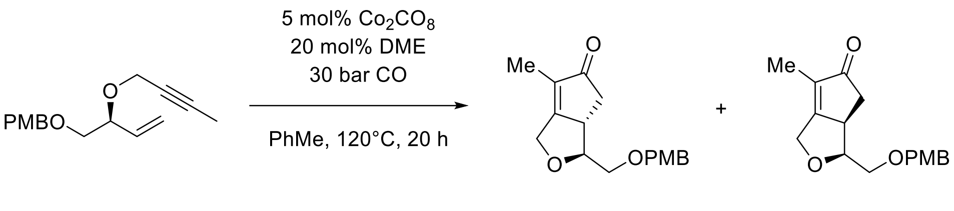

Chemical Formula: $C_{17}H_{20}O_4$
Exact Mass: 288,14
Molecular Weight: 288,34

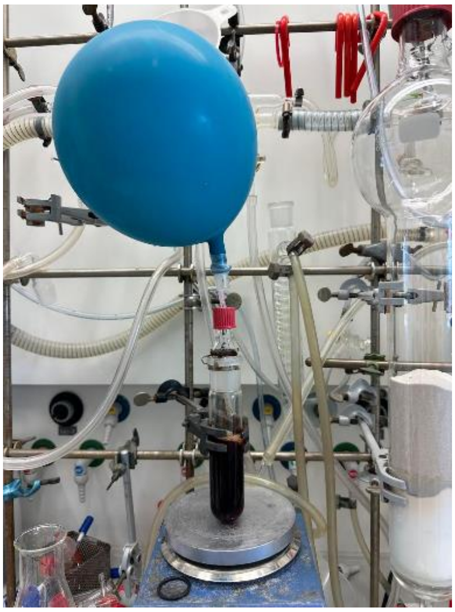

The reaction was performed using a modified procedure by Sugihara.[3]

In a dried tube with a septum cap purged with argon and equipped with a cross-shaped stir bar, enyne (7.0 g, 33.6 mmol) was dissolved in dry toluene (55 mL) and dimethoxymethane (0.56 mL, 0.2 eq.). Then, Co_2CO_8 (458 mg, 1.7 mmol, 5 mol%, weighed in glovebox) was added and stirred until CO evolution ceased (~10 min). The tube was placed in a steel autoclave with the balloon detached from the needle to ensure gas permeability. The autoclave was purged with Ar (3 x 5 bar) and CO (3 x 5 bar), pressurized with 30 bars of CO and placed in a heating block heated to 120 °C overnight (20 h). The pressure was released and the solvent was removed under reduced pressure. A second run was performed with 4.0 grams of enyne (19.2 mmol). The combined crude products were purified by column chromatography (silica gel, Et_2O/petroleum ether 4:1) to yield main isomer **20** (9.48 g, 32.9 mmol, 78%) and diastereomer (353 mg, 1.22 mmol, 3%) as amber, honey-like oils.

Notes:

A sufficient vortex needs to form inside the reaction tube in the autoclave and a cross shaped stir bar was found to be the most effective. Lower yields due to lower conversion were obtained for some smaller scale reactions where vigorous stirring was not controlled for before setup.

major (20)

^{1}H NMR (500 MHz, $CDCl_3$) δ = 7.18 – 7.12 (m, 2H), 6.80 – 6.75 (m, 2H), 4.56 (br. d, *J* = 15.3, 1H), 4.44 (br. d, *J* = 15.3, 1H), 4.43 (s, 2H), 3.69 (s, 3H), 3.58 (dd, *J* = 10.4, 4.2 Hz, 1H), 3.54 (dd, *J* = 10.4, 5.2 Hz, 1H), 3.45 (ddd, *J* = 10.3, 5.2, 4.2 Hz, 1H), 2.86 (m, 1H), 2.49 (dd, *J* = 17.9, 6.4 Hz, 1H), 2.01 (dd, *J* = 17.9, 3.1 Hz, 1H), 1.64 (dt, *J* = 2.5, 1.3 Hz, 3H).

^{13}C NMR (126 MHz, $CDCl_3$) δ = 208.9, 176.0, 159.4, 132.9, 130.1, 129.4, 114.0, 82.3, 73.4, 70.7, 65.6, 55.4, 45.4, 38.6, 9.0.

HR-MS (APCI+): m/z calc. for $C_{17}H_{21}O_4^+$ $[M+H]^+$: 289.1434, found 289.1437.

[a]^{D}$_{25.6}$ = -38.4° ($CHCl_3$, c = 1.0). **[a]^{D}$_{22.0}$** (**ent-20**) = + 41.7° ($CHCl_3$, c = 1.0).

R_f (hexanes/Et_2O 1:1) = 0.4.

minor

^{1}H NMR (500 MHz, $CDCl_3$) δ 7.16 – 7.10 (m, 2H), 6.86 – 6.83 (m, 2H), 4.54 (br. s, 2H), 4.38 (ddd, *J* = 8.3, 6.0, 3.9 Hz, 1H), 4.35 (d, *J* = 11.5 Hz, 1H), 4.31 (d, *J* = 11.5 Hz, 1H), 3.79 (s, 3H), 3.37 (dd, *J* = 10.1, 3.9 Hz, 1H), 3.35 (m, 1H), 3.33 (dd, *J* = 10.1, 6.0 Hz, 1H), 2.60 (dd, *J* = 18.0, 6.5 Hz, 1H), 2.39 (dd, *J* = 18.0, 3.6 Hz, 1H), 1.69 (dt, *J* = 2.5, 1.3 Hz, 3H).

^{13}C NMR (126 MHz, $CDCl_3$) δ = 209.9, 175.7, 159.4, 129.8, 129.3, 113.9, 77.3, 76.7, 73.3, 69.5, 65.1, 55.3, 44.9, 37.5, 9.0.

[a]^{D}$_{25.6}$ = + 111.4° ($CHCl_3$, c = 1.0).

R_f (hexanes/Et_2O 1:1) = 0.3.

(*S*)-4-((*S*)-1-hydroxy-2-((4-methoxybenzyl)oxy)ethyl)-2,3-dimethylcyclopent-2-en-1-one (21a)

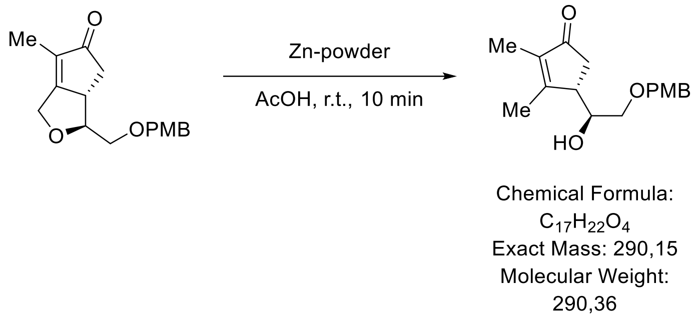

In an Erlenmeyer flask equipped with a sufficiently powerful stir bar Zinc powder (18.4 g, 281 mmol) was suspended in glacial acetic acid (450 mL) and cyclopentenone **20** (9.45 g, 32.8 mmol) was added. After 10 minutes, TLC showed complete and clean conversion. The suspension was diluted with Et_2O and the unreacted zinc and $Zn(OAc)_2$ was fritted off. The solvent was removed under reduced pressure and the crude product - still containing some acetic acid and $Zn(OAc)_2$ - was filtered through a short silica plug using Et_2O/MeOH 10:1. After evaporation of the solvent under reduced pressure the title compound **21a** (9.49 g, 32.7 mmol, quant.) was obtained as a clear yellowish oil.

Notes:

For TLC control, an aliquot was given onto water/Et_2O to separate out the acetic acid which influences the observed R_f.

Without using methanol for the silica filtration, significant amounts of product stick to the silica even with considerable elution with ether or ethyl acetate.

^{1}H NMR (400 MHz, $CDCl_3$) δ 7.24 – 7.20 (m, 2H), 6.91 – 6.86 (m, 2H), 4.48 – 4.40 (m, 2H), 3.95 (td, *J* = 7.3, 3.2 Hz, 1H), 3.81 (s, 3H), 3.37 (dd, *J* = 9.5, 3.2 Hz, 1H), 3.29 (dd, *J* = 9.5, 7.7 Hz, 1H), 3.02 – 2.94 (m, 1H), 2.62 (s, OH), 2.41 (dd, *J* = 18.8, 6.8 Hz 1H), 2.23(dd, *J* = 18.8, 2.3 Hz, 1H), 2.08 (s, 3H), 1.68 (m, 3H).

^{13}C NMR (101 MHz, $CDCl_3$) δ 208.1, 170.0, 159.6, 138.0, 129.6, 129.5, 114.1, 73.3, 72.0, 71.4, 55.4, 45.8, 37.1, 16.9, 8.3.

HR-MS (APCI+): m/z calc. for $C_{17}H_{22}O_4$ $[M]^+$: 290.1513, found: 290. 1514.

$[a]^D_{27.0}$ = -28.0° ($CHCl_3$, c = 1.0). **$[a]^D_{22.0}$** (enantiomer) = + 24.7° ($CHCl_3$, c = 1.0).

R_f (Et_2O) = 0.2.

(*S*)-4-((*S*)-1,2-dihydroxyethyl)-2,3-dimethylcyclopent-2-en-1-one (21b)

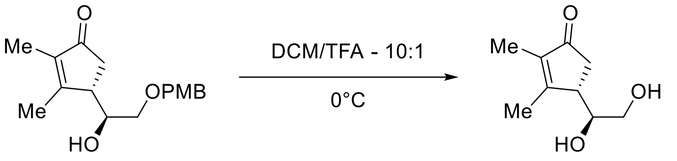

Chemical Formula: $C_9H_{14}O_3$
Exact Mass: 170,09
Molecular Weight: 170,21

The deprotection was performed along a modified literature procedure substitution TfOH for TFA.[4]

A mixture of DCM and TFA (10:1, 500 mL) was cooled to 0°C and alcohol **21a** (8.74 g, 30.1 mmol) was added swiftly. After 10 minutes, TLC conversion was complete and the solvent was removed under reduced pressure. The crude mixture was purified via column chromatography (silica gel, EtOAc/MeOH – 10:0 to 9:1) to yield diol **21b** (4.252 g, 25.0 mmol, 83%) as a yellow viscous oil.

^{1}H NMR (400 MHz, $CDCl_3$ δ 3.91 (ddd, *J* = 8.0, 6.7, 3.1 Hz, 1H), 3.56 (dd, *J* = 10.9, 3.0 Hz, 1H), 3.46 (dd, *J* = 11.0, 8.0 Hz, 1H), 3.01 (m_c, 3H), 2.45 (dd, *J* = 18.9, 6.8 Hz, 1H), 2.27 (dd, *J* = 18.9, 2.1 Hz, 1H), 2.11 (m_c, 3H), 1.68 (m_c, 3H).

^{13}C NMR (101 MHz, $CDCl_3$) δ 208.5, 170.3, 138.2, 73.4, 64.0, 46.0, 37.2, 17.0, 8.1.

HR-MS (APCI+): m/z calc. for $C_9H_{15}O_3$ $[M+H]^+$: 171.1016, found: 171.1020.

$[a]^D_{27.0}$ = -31.2° ($CHCl_3$, c = 1.0).

R_f (Et_2O) = 0.2.

(*S*)-4-((*S*)-1-((tert-butyldimethylsilyl)oxy)-2-((4-methoxybenzyl)oxy)ethyl)-2,3-dimethylcyclopent-2-en-1-one (21c)

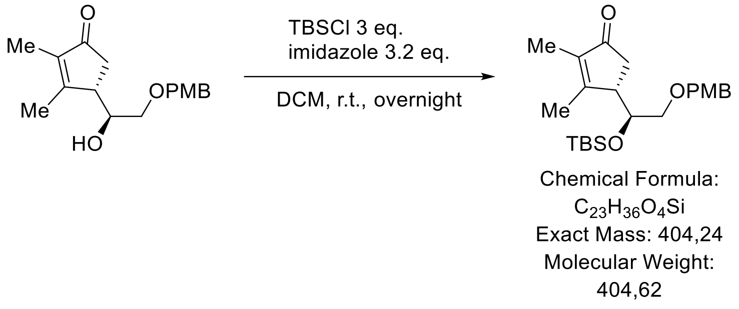

In an oven-dried round bottom flask equipped with a stir bar, alcohol **21a** (690 mg, 2.34 mmol) was dissolved in dry DCM (10 mL) and imidazole (510 mg, 7.49 mmol, 3.2 eq.) and TBSCl (1.05 g, 7.03 mmol, 3.0 eq.) were added. The reaction was stirred overnight. The mixture was diluted with Et_2O (20 mL), the precipitate was fritted off and the solvent was removed under reduced pressure. The crude product was purified via short column chromatography (silica gel, hexanes/Et_2O – 4:1) to yield the title compound **21c** (927 mg, 2.29 mmol, 98%) as a viscous yellow oil.

^{1}H NMR (400 MHz, $CDCl_3$) δ 7.20 – 7.14 (m, 2H), 6.89 – 6.82 (m, 2H), 4.37 – 4.27 (m, 2H), 4.17 (td, *J* = 5.7, 4.2 Hz, 1H), 3.80 (s, 3H), 3.32 (dd, *J* = 9.8, 5.7 Hz, 1H), 3.25 (dd, *J* = 9.8, 5.8 Hz, 1H), 3.06 – 2.99 (m, 1H), 2.39 – 2.35 (m, 2H), 1.99 (m_c, 3H), 1.65 (m_c, 3H), 0.85 (s, 9H), 0.06 (s, 6H).

^{13}C NMR (101 MHz, $CDCl_3$) δ 208.9, 169.6, 159.3, 137.8, 130.2, 129.3, 113.8, 73.1, 72.1, 71.8, 55.4, 47.7, 37.4, 25.8, 18.1, 16.6, 8.0, -4.3, -4.9.

HR-MS (APCI+): m/z calc. for $C_{23}H_{37}O_4Si^+$ $[M+H]^+$: 405.2456, found: 405.2460.

$[a]^{D}_{22.0}$ = -40.6° ($CHCl_3$, c = 1.0).

R_f (PE/Et_2O - 4:1) = 0.8.

(1*S*,2*S*,5*S*)-1,5-dimethyl-6-methylene-4-oxobicyclo[3.2.0]heptane-2-carbaldehyde (endo, 24b)

and

(1*R*,2*S*,5*R*)-1,5-dimethyl-6-methylene-4-oxobicyclo[3.2.0]heptane-2-carbaldehyde (exo, 24a)

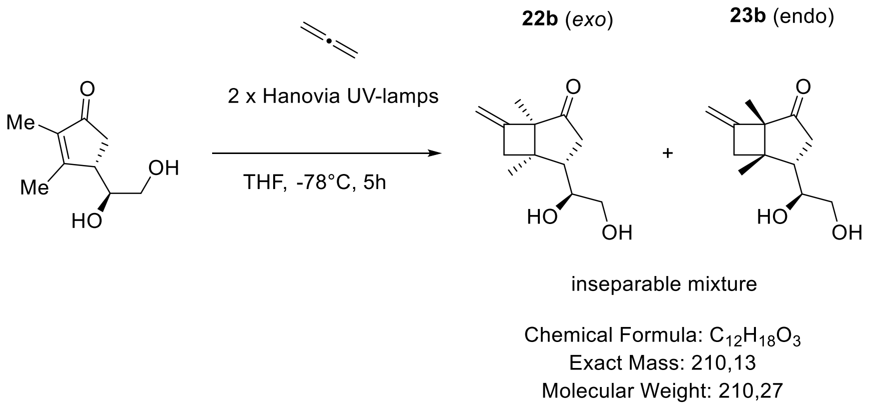

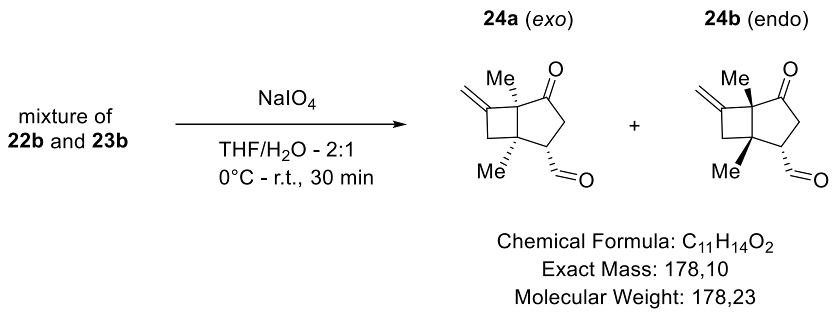

In the apparative setup described for **ent-22c**, compound **21b** (1.65 g, 9.69 mmol) was dissolved in degassed THF (400 mL) under argon, cooled to -78°C and propadiene gas (~2 L, ~0.1 mol, ~10 eq.) were bubbled into the solution. Under continuous addition of dry ice, the reaction was irradiated with two Hanovia mercury lamps, immersed parallel to the reactor. After 5 h of irradiation conversion significantly slowed and was stopped at 93% (determined via NMR). Using cold Et_2O, some the *exo* isomer could be slightly enriched by trituration. The crude mixture was purified via column chromatography (silica gel, EtOAc) to yield an inseparable mixture of isomers **22b** and **23b** (1.577 g, 7.50 mmol, 78%). The procedure was repeated in similar scale.

Sodium periodate (3.96 g, 18.4 mmol, 1.3 eq.) was dissolved in water (55 ml) and THF (110 mL) was added. The mixture was cooled to 0°C and a solution of diol mixture **22/23b** (2.99 g, 14.2 mmol, exo/endo 1:1) was swiftly added. The mixture was warmed to r.t. and stirred for 30 minutes. The mixture was diluted with water (100 mL) and extracted with Et_2O (200 mL). The solvent was removed under reduced pressure and the mixture was purified via column chromatography (silica gel, hexanes/EtOAc – 9:2) to yield *endo*-product **24b** (1.209 g, 6.78 mmol, 37%, >90% pure) as a yellowish oil that solidified in the freezer and two fractions of partially epimerized *exo*-product **24a** (fraction 2: 802 mg, *exo/endo* 3:1, fraction 2: 204 mg, *exo/endo* 7:1, 31%) as yellowish oils.

Notes:

During chromatography of 24a/24b, a gap between pure fractions of endo-aldehyde and endo/exo-fractions was noticed. This implies that the epimerization is slow enough for the isomers to be separated without reducing the enantiomeric excess of the endo-aldehyde (the epimer of the exo-aldehyde is the enantiomer of the endo-aldehyde in the first fractions).

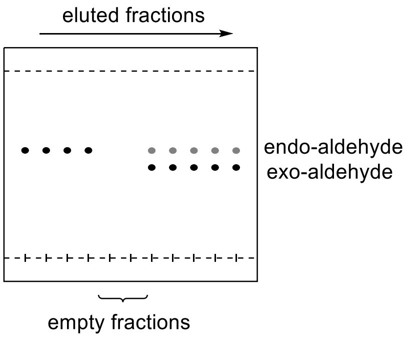

***endo*-Isomer (24b)**

^{1}H NMR (700 MHz, C_6D_6) δ 9.22 (d, *J* = 1.3 Hz, 1H), 4.87 (td, *J* = 2.8, 0.9 Hz, 1H), 4.60 (td, *J* = 2.3, 0.9 Hz, 1H), 2.72 (dd, *J* = 17.7, 12.2 Hz, 1H), 2.46 (dt, *J* = 16.9, 2.7 Hz, 1H), 1.99 – 1.94 (m, 2H), 1.90 (ddd, *J* = 12.1, 8.1, 1.4 Hz, 1H), 0.97 (s, 3H), 0.85 (s, 3H).

^{13}C NMR (176 MHz, C_6D_6) δ 212.4, 199.7, 146.5, 108.0, 63.3, 55.3, 43.4, 36.6, 36.0, 20.9, 13.6.

$[a]^D_{22.0}$ = +156.7° ($CHCl_3$, c = 1.0).

HR-MS (APCI+): m/z calc. for $C_{11}H_{15}O_2^+$ $[M+H]^+$: 179.1067, found: 179.1064.

R_f (PE/EE - 4:1) = 0.5 (*endo*), 0.4 (*exo*).

Exo-isomer **24a** contained in the second fraction matched NMR-data of pure exo-isomer **ent-24a** described in the synthesis via (*S*)-glycidol.

3.2 Isomerization-based route from (*S*)-glycidol

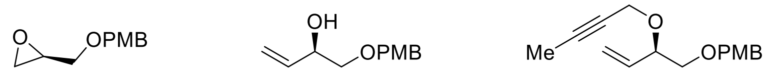

ent-17 quant. | **ent-18** 87% | **ent-19** 78%

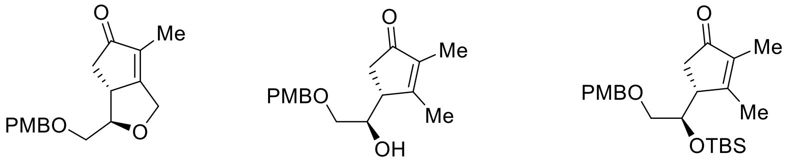

ent-20 82% | **ent-21a** quant. | **ent-21c** 97%

54% over 6 steps
from (S)-gylcidol

For the isomerization-based route the enantiomeric cyclopentenone **ent-21c** was synthesized in analogous fashion with slightly lower yield in bigger scale with matching analytical data (yields given for each step).

(1S,4R,5S)-4-((R)-1-((tert-butyldimethylsilyl)oxy)-2-((4-methoxybenzyl)oxy)ethyl)-1,5-dimethyl-7-methylenebicyclo[3.2.0]heptan-2-one (exo-isomer) (ent-22c)

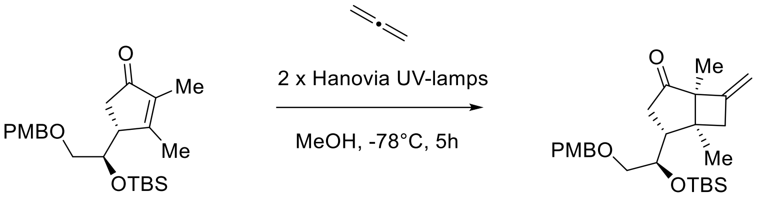

CAUTION: The UV-radiation emitted by the UV-lamps in this experiment is very intense. Proper eye protection must be worn when handling these lamps during operation.

In a photochemical quartz reactor equipped with an immersion thermometer, enone **ent-21c** (7.56 g, 18.7 mmol) was dissolved in degassed MeOH (350 mL), cooled to -78 °C and a balloon filled with propadiene gas (~ 2 liters, ~ 0.1 mol, 35 eq.) was bubbled through and the reactor was equipped with an argon balloon. Two Hanovia UV-lamps were immersed into the dry ice bath alongside the reactor on opposite sides and irradiation was started. Considerable heat generation by the UV-lamps required shoveling dry ice into the bath every ~20 minutes to keep the internal temperature at roughly -75 to -78°C. After 11 h conversion slowed significantly and the reaction was stopped at ~83% NMR-conversion. The reactor was opened and the mixture transferred to a round bottom flask and warmed to room temperature under stirring to avoid spontaneous degassing of the excess propadiene. After evaporation of the solvent, the crude product was purified via column chromatography to yield the starting material (1.15 g, 2.8 mmol) and the title compound **ent-22c** (6.37 g, 14.3 mmol, 77%, 90% *brsm*) as a clear viscous oil.

Notes:

The purified product is roughly 90% pure with signals indicating other isomers, that are inseparable via chromatography.

^{1}H NMR (700 MHz, $CDCl_3$)f δ 7.23 – 7.20 (m, 2H), 6.89 – 6.84 (m, 2H), 4.92 (td, *J* = 2.7, 0.8 Hz, 1H), 4.80 (td, *J* = 2.2, 0.7 Hz, 1H), 4.41 (d, *J* = 11.7 Hz, 1H), 4.34 (d, *J* = 11.7 Hz, 1H), 3.94 (dt, *J* = 6.0, 4.2 Hz, 1H), 3.80 (s, 3H), 3.26 (dd, *J* = 9.7, 4.1 Hz, 1H), 3.23 (dd, *J* = 9.7, 6.0 Hz, 1H), 2.70 (dt, *J* = 16.0, 2.3 Hz, 1H), 2.66 (q, *J* = 9.7 Hz, 2H), 2.58 (dt, *J* = 16.0, 2.5 Hz, 1H), 2.38 – 2.32 (m, 2H), 1.17 (s, 3H), 1.05 (s, 3H), 0.86 (s, 9H), 0.05 (s, 3H), 0.04 (s, 3H).

^{13}C NMR (176 MHz, $CDCl_3$) δ 217.4, 159.3, 147.4, 130.2, 129.5, 113.9, 108.1, 73.4, 73.0, 72.7, 72.4, 62.0, 48.3, 44.4, 43.5, 40.4, 26.0, 18.2, 17.5, 14.8, -4.1, -4.7.

HR-MS (APCI+): m/z calc. for $C_{26}H_{41}O_4Si^+$ $[M+H]^+$: 445.2769, found: 445.2776.

$[a]^{D}_{22.0}$ = -21.3 ($CHCl_3$, c = 1.0).

R_f (PE/EtOAc – 10:1) = 0.5.

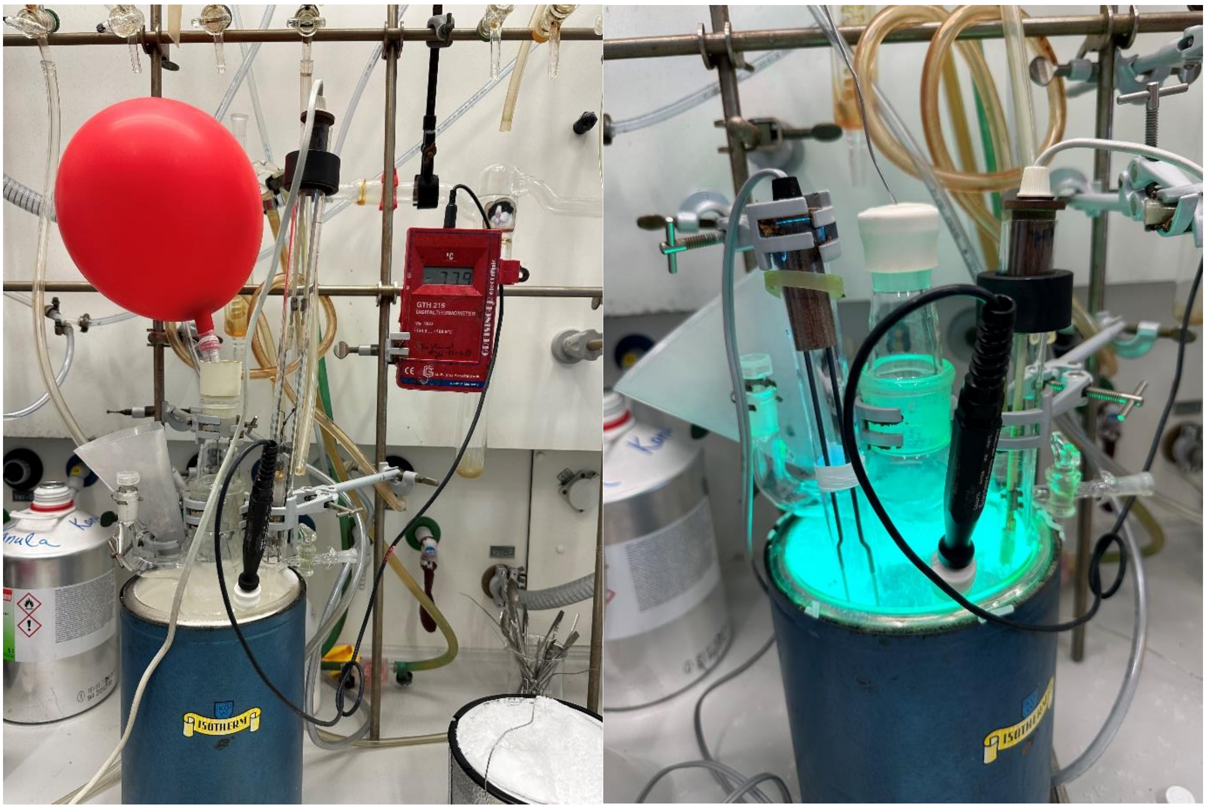

Left: Photoreactor setup featuring an immersion thermometer, *Hanovia* UV lamp, dry-ice bath, and funnel for dry ice addition. *Right*: Photoreactor during irradiation.
Important: *The reactor is very bright. For that reason, it is covered with aluminum foil in such a way that the funnel is open for dry ice addition. During an irradiation of 11 h, roughly 12 kg of dry ice are consumed.*

(1*S*,4*R*,5*S*)-4-((*R*)-1,2-dihydroxyethyl)-1,5-dimethyl-7-methylenebicyclo[3.2.0]heptan-2-one (ent-22b)

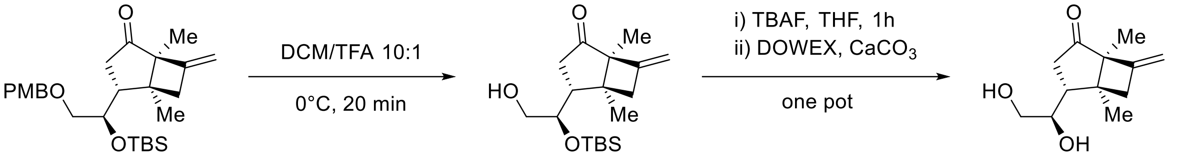

Chemical Formula: $C_{12}H_{18}O_3$
Exact Mass: 210,13
Molecular Weight: 210,27

Ether **ent-22c** (6.0 g, 13.5 mmol) was dissolved in dry DCM (200 mL) and cooled to 0°C. TFA (20 mL) was swiftly added and stirred under argon for 20 minutes. The mixture was poured onto water (200 mL) and extracted two times with DCM (~200 mL). The solvent was removed under reduced pressure and the crude mixture was purified via column chromatography (silica gel, hexanes/EtOAc 4:1) to yield alcohol (4.18 g, 95%) as a colorless oil that crystallized to a waxy solid in the freezer.

The workup in the following step, to remove excess TBAF, was performed following a modified literature procedure.[5]

All of the product above was dissolved in THF (25 mL), cooled to 0°C and TBAF (40 mmol, 3.1 eq., 1M in THF, 5wt% water) was added swiftly, warmed to r.t. and stirred for 1 h. Then $CaCO_3$ (6g) and DOWEX-resin (18g, acidic form) were added and immediate bubbling was observed. The mixture was stirred vigorously for 1h. The solids were filtered over a frit and the solvent was removed under reduced pressure. The residue was taken up in EtOAc and filtered through a silica plug to remove residual traces of TBAF. The solvent was removed under reduced pressure and the residue was triturated with Et_2O/hexanes to yield isomerically pure diol **ent-22b** (2.19 g, 12.8 mmol, 81%) as a fluffy white powder.

Notes:

The diol is prone to bumping in the rotovap when the evaporated solvent is EtOAc, so a sufficiently large flask should be used (250 mL in this case). When left to crystallize from residual solvent it forms circle-shaped crystals. The yield of the second step is near quantitative, however it still contains roughly 10% isomers from the photochemical step. Trituration yields a product devoid of these isomers albeit with some loss of the main product.

^{1}H NMR (700 MHz, $CDCl_3$) δ 4.97 (td, *J* = 2.7, 0.8 Hz, 1H), 4.86 (td, *J* = 2.1, 0.8 Hz, 1H), 3.80 (tdd, *J* = 7.7, 3.8, 2.8 Hz, 1H), 3.63 (ddd, *J* = 10.8, 5.1, 2.9 Hz, 1H), 3.48 (ddd, *J* = 10.8, 7.9, 5.0 Hz, 1H), 2.66 (m, 2H), 2.57 (dd, *J* = 17.4, 7.6 Hz, 1H), 2.33 (dt, *J* = 8.8, 7.7 Hz, 1H), 2.26 (br. d, *J* = 3.8 Hz, 1H), 2.18 (dd, *J* = 17.4, 8.7 Hz, 1H), 1.77 (br. t, *J* = 5.2 Hz, 1H), 1.25 (s, 3H), 1.16 (s, 3H).

^{13}C NMR (176 MHz, $CDCl_3$) δ 216.5, 147.0, 108.5, 73.5, 65.4, 62.2, 46.2, 44.0, 43.4, 41.2, 16.5, 15.1.

HR-MS (APCI+): m/z calc. for $C_{12}H_{19}O_3^+$ $[M+H]^+$: 211.1329, found: 211.1334.

$[a]^D_{22.0}$ = +51.9° ($CHCl_3$, c = 1.0). ***T_m*** = 109 °C.

R_f (EtOAc) = 0.5.

(1*S*,2*R*,5*S*)-1,5-dimethyl-6-methylene-4-oxobicyclo[3.2.0]heptane-2-carbaldehyde (*exo,* ent-24a)

and

(1*S*,2*S*,5*S*)-1,5-dimethyl-6-methylene-4-oxobicyclo[3.2.0]heptane-2-carbaldehyde (*endo,* 24b)

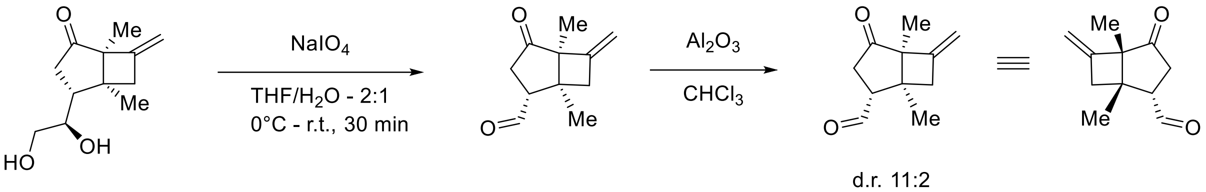

d.r. 11:2

Chemical Formula: $C_{11}H_{14}O_2$
Exact Mass: 178,10
Molecular Weight: 178,23

Sodium periodate (2.64 g, 18.4 mmol, 1.3 eq.) was dissolved in water (35 ml) and THF (75 mL) was added. The mixture was cooled to 0°C and a solution of diol mixture (1.50 g, 9.51 mmol) was swiftly added. The mixture was warmed to r.t. and stirred for 1h. The mixture was diluted with water (100 mL) and extracted with Et_2O (200 mL). The solvent was removed under reduced pressure and the turbid oil was filtrated through a silica frit with Et_2O yielding the *exo*-aldehyde **ent-24a** (1.640 g, 9.20 mmol, 97%) as a clear oil.

The aldehyde (1.50 g, 8.42 mmol) was dissolved in $CHCl_3$ (10 mL) and added to a degassed suspension of basic alumina (3 g) in $CHCl_3$ (130 mL) under argon. The solution was stirred gently overnight (16 h) and filtrated through a frit. After removal of the solvent under reduced pressure, the isomeric mixture of aldehydes (1.498 g, 8.40 mmol, d.r. 11:2, 99%) as a mobile oil with a green tinge. The mixture was purified via column chromatography (silica gel, hexanes/EtOAc 9:2) to yield aldehyde **24b** (1.249 g, 7.00 mmol, 83%) as a clear oil with a yellow tinge.

ent-24a (*exo*)

^{1}H NMR (400 MHz, C_6D_6) δ 9.21 – 9.20 (m, 1H), 4.93 (td, *J* = 2.8, 0.8 Hz, 1H), 4.65 (td, *J* = 2.2, 0.8 Hz, 1H), 2.34 (m, 1H), 2.24 – 2.15 (m, 3H), 2.04 (ddd, *J* = 16.3, 2.7, 2.1 Hz, 1H), 1.01 (s, 3H), 0.77 (s, 3H).

^{13}C NMR (101 MHz, C_6D_6) δ 213.1, 202.1, 146.0, 108.2, 61.5, 54.6, 45.0, 43.0, 37.0, 17.7, 14.3.

HR-MS (APCI+): m/z calc. for $C_{11}H_{15}O_2^+$ $[M+H]^+$: 179.1067, found: 179.1064.

$[a]^D_{22.0}$ = +25.2° ($CHCl_3$, c = 1.0).

24b (*endo*) matched analytical data with 24b from the procedure via the (*R*)-glycidol route.

Notes:

Some volatility of the compounds was assumed. Combined with the tendency to epimerize, they were not left in the rotovap at <30mbar/40°C for more than 5 minutes.

(1*S*,4*S*,5*S*)-4-(but-2-ynoyl)-1,5-dimethyl-7-methylenebicyclo[3.2.0]heptan-2-one (25)

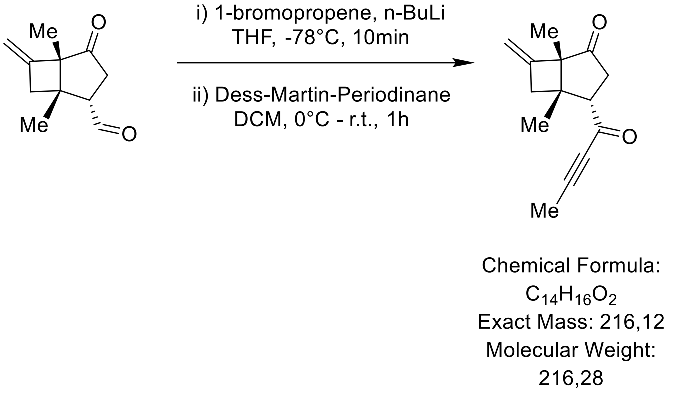

The following generation of propynyllithium was performed after a modified procedure described in literature.[6]

To a heatgun-dried cone-bottom flask equipped with a football-shaped stir-bar 1-bromopropene (1.27 g, 10.5 mmol, 1.5 eq., 892 µL) was dissolved in dry THF (8.4 mL). The solution was cooled to -78°C and *n*-BuLi (15.4 mmol, 6.4 mL, 2.4 M in hexanes) was added dropwise under vigorous stirring. The solution was stirred at -78°C for 2h. In a separate heatgun-dried three-neck round bottom flask aldehyde **24b** (1.259 g, 7.0 mmol) was dissolved in THF under argon (16 mL) and cooled to -78°C. Working swiftly, the prepared propynyl-lithium solution was pulled into a syringe and added dropwise to the solution of aldehyde under vigorous stirring. The reaction was stirred for 10 minutes until an aliquot quenched with aq. NH_4Cl showed full TLC-conversion. When complete, the reaction was quenched with sat. aq. NH_4Cl (20 mL) and water (20 mL) and extracted with Et_2O (200 mL). The organic phase was dried with Na_2SO_4 and the solvent removed under reduced pressure. The crude mixture of alcohols (d.r. ~2:1) was redissolved in dry DCM (35 mL) and cooled to 0°C. Dess-Martin-periodinane (3.71 g, 8.25 mmol, 1.25 eq.) was added in one portion and the mixture was stirred for 1h. The mixture was poured on sat. aq. $NaHCO_3$ (100 mL), extracted with Et_2O (3 x 50 mL) and the solvent was removed under reduced pressure. The crude mixture was purified via column chromatography (SiO_2, hexanes/EtOAc 4:1) to yield the title compound **25** (1.027 g, 4.75 mmol, 68% over 2 steps) as a clear oil.

^{1}H NMR (700 MHz, $CDCl_3$) δ 4.95 (td, *J* = 2.8, 0.9 Hz, 1H), 4.84 (td, *J* = 2.3, 1.0 Hz, 1H), 3.25 (dd, *J* = 18.3, 12.5 Hz 1H), 3.04 (dd, *J* = 12.5, 7.9 Hz, 1H), 2.95 (dt, *J* = 17.2, 2.7 Hz, 1H), 2.46 (dd, *J* = 18.3, 7.9 Hz, 1H), 2.40 (dt, *J* = 17.2, 2.5 Hz, 1H), 2.07 (s, 3H), 1.46 (s, 3H), 1.13 (s, 3H).

^{13}C NMR (176 MHz, $CDCl_3$) δ 215.0, 186.5, 146.0, 108.3, 92.9, 81.3, 63.7, 57.3, 45.0, 38.6, 37.0, 21.4, 13.8, 4.3.

HR-MS (APCI+): m/z calc. for $C_{14}H_{17}O_2^+$ [M+H]$^+$: 217.1223, found: 217.1224.

$[a]^D_{22.0}$ = 55.5 ($CHCl_3$, c = 1.0).

R_f (PE/Et_2O – 7:3) = 0.25.

(3aS,9S)-1,4,9-trimethyl-6,7-dihydro-3H-4,7,3a-(epiethane[1,1,2]triyl)azulene-2,5,8(4H)-trione (27)

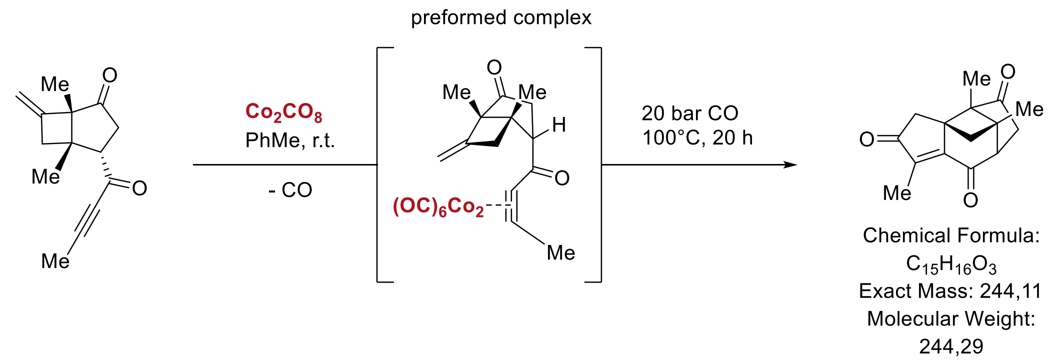

Small scale:

In a heat-gun dried Schlenk-tube under argon, alkyne **25** (39.5 mg, 0.183 mmol) was dissolved in toluene (4 mL) and cobalt octacarbonyl (65.8 mg, 1.05 eq.). The mixture was stirred until no gas evolution was observed and the TLC indicated full conversion to the complex **26**. The mixture was transferred to a septum capped glass inlet equipped with an argon balloon via syringe and the inlet was placed into a steel autoclave. The balloon was removed and the autoclave was sealed and flushed with argon (3 x 5 bar) and CO (3x 5 bar) and put under 20 bars of CO-pressure. The autoclave was heated in a heating block at 120 °C for 20 h. The pressure was released and the solvent removed under reduced pressure. The crude mixture was purified via column chromatography (silica gel, hexanes then Et_2O) to yield the title compound **27** (37.0 mg, 83%) as a white solid.

Larger scale:

Large scale reactions were performed at higher concentrations in parallel fashion:

Following the procedure above, three scale up experiments using 250 mg, 350 mg and 350 mg* of alkyne **25** dissolved in 25 mL of toluene yielded 216.4 mg (77%), 268.5 (68%) and 203.9 (52%) respectively giving a total yield of 725.8 mg (65% weighted *avg*. for 4 reactions).

^{1}H NMR (700 MHz, C_6D_6) δ 2.51 (dd, *J* = 7.6, 0.9 Hz, 1H), 2.20 (d, *J* = 19.7 Hz, 1H), 2.05 (dd, *J* = 18.2, 7.6 Hz, 1H), 2.01 (dd, *J* = 18.2, 0.9 Hz, 1H), 1.95 (s, 3H), 1.63 (d, *J* = 19.7 Hz, 1H), 1.54 (d, *J* = 10.3 Hz, 1H), 1.21 (d, *J* = 10.3 Hz, 1H), 0.73 (s, 3H), 0.37 (s, 3H).

^{13}C NMR (176 MHz, C_6D_6) δ 213.3, 204.8, 198.3, 159.6, 142.3, 61.3, 55.9, 52.6, 44.7, 43.9, 39.4, 38.6, 16.8, 10.5, 8.9.

HR-MS (APCI+): m/z calc. for $C_{15}H_{17}O_3^+$ [M+H]$^+$: 245.1172, found: 245.1170.

$[a]^D_{22.0}$ = + 248.5° ($CHCl_3$, c = 1.0). ***T_m*** = 144 °C.

R_f (Et_2O) = 0.5. **Co-Alkyne-Complex: R_f** (hexanes/EtOAc – 4:1) = 0.6.

Notes:

For one of the larger scale reactions, a taller autoclave was used, yield was low in an initial attempt. Reasons for that could be that the internal temperature might be significantly lower, since a bigger part of the autoclave is not covered by the heating block.*

(3a*S*,4*R*,7*S*,8*S*,9*S*)-8-hydroxy-1,4,7,9-tetramethyl-7,8-dihydro-*3H*-4,7,3a-(epiethane[1,1,2]triyl)azulene-2,5(*4H,6H*)-dione

Daphnepapytone A (2)

and

(1*S*,2a*R*,2a^1*R*,3a*R*,4*S*,6a*R*,7*aS*,8*S*)-8-hydroxy-2a^1,4,7a-trimethylhexahydro-4*H*-3-oxa-1,3a-methanocyclobuta[*gh*]cyclopenta[*a*]pentalen-5(*6H*)-one (28)

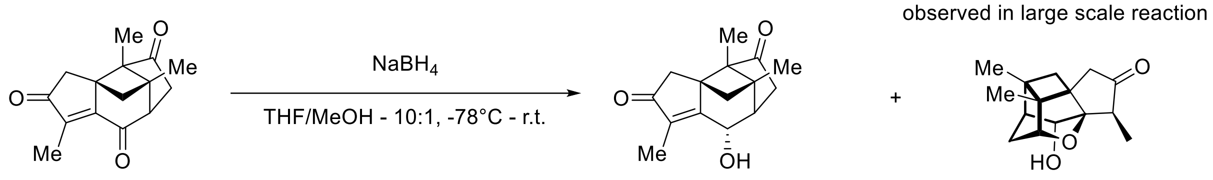

2

Chemical Formula: $C_{15}H_{18}O_3$
Exact Mass: 246,13
Molecular Weight: 246,31

28

Chemical Formula: $C_{15}H_{20}O_3$
Exact Mass: 248,14
Molecular Weight: 248,32

Small scale:

In a heat-gun dried Schlenk tube flushed with argon triketone (sourced from initial route from (*R*)-glycidol) (50.0 mg, 0.205 mmol) was dissolved in a 10:1 mixture of dry THF/MeOH (3 mL) and cooled to -78°C. Then ground $NaBH_4$ (15.8 mg. 0.418 mmol, 2.04 eq.) was added and stirred for 2h at the same temperature with TLC indicating incomplete conversion. The cooling bath was removed and during warming to r.t. bubbling was observed. At r.t. conversion was determined to be complete and the reaction was quenched with aq. sat. NH_4Cl (2 mL). The mixture was extracted with Et_2O and the solvent was removed under reduced pressure. The crude product was purified by column chromatography (silica gel, Et_2O) to yield the final natural compound (40.4 mg, 0.165 mmol, 80%) as white powder.

Large scale:

In a heat-gun dried three-necked round bottom flask flushed with argon triketone (638.2 mg, 2.59 mmol) was dissolved in a 10:1 mixture of dry THF/MeOH (32 mL) and cooled to -78°C. Then ground $NaBH_4$ (107.8 mg. 2.85 mmol, 1.1 eq.) was added and stirred for 2h at the same temperature with TLC indicating incomplete conversion. The cooling bath was removed and during warming to r.t. bubbling was observed. At r.t. conversion was determined to be complete and the reaction was quenched with aq. sat. NH_4Cl (10 mL). The mixture was extracted with Et_2O and the solvent was removed under reduced pressure. The crude product was purified by column chromatography (silica gel, Et_2O) to yield the final natural compound **2** (422.0 mg, 1.71 mmol, 66%) as a colorless crystalline solid and compound **28** (95.7 mg, 0.39 mmol, 15%) as a white powder.

(+)-Daphnepapytone A (2)

^{1}H NMR (700 MHz, $CDCl_3$) δ 5.35 – 5.29 (m, 1H), 2.80 (d, *J* = 18.3 Hz, 1H), 2.62 (t, *J* = 6.8 Hz, 1H), 2.43 (d, *J* = 19.3 Hz, 1H), 2.31 (ddd, *J* = 18.3, 7.2, 1.4 Hz, 1H), 2.17 (d, *J* = 10.0 Hz, 1H), 2.14 (d, *J* = 5.6 Hz, 1H), 2.13 (d, *J* = 10.0 Hz, 1H), 2.09 (dd, *J* = 19.3, 1.4 Hz, 1H), 1.86 (d, *J* = 1.9 Hz, 3H), 1.14 (s, 3H), 1.13 (s, 3H).

^{13}C NMR (176 MHz, $CDCl_3$) δ 217.8, 206.5, 171.1, 136.6, 68.9, 61.2, 54.4, 45.5, 44.8, 40.1, 39.5, 35.8, 18.6, 10.6, 8.2.

HR-MS (APCI+): m/z calc. for $C_{15}H_{19}O_3^+$ [M+H]$^+$: 247.1329, found: 247.1329.

[a]$^D_{22.0}$ = + 52.2 ° ($CHCl_3$, c = 1.0). ***T_m*** = 166 - 171 °C.

R_f (Et_2O) = 0.3.

The structure was confirmed via X-Ray and the analytical data is in accordance with the literature.[7–11]

Compound 28

^{1}H NMR (700 MHz, $CDCl_3$) δ 4.03 (d, *J* = 5.0 Hz, 1H), 3.97 – 3.95 (m, 1H), 2.37 (d, *J* = 19.9 Hz, 1H), 2.34 (qd, *J* = 7.0, 1.5 Hz, 1H), 2.21 (dddd, *J* = 4.9, 4.0, 2.1, 0.8 Hz, 1H), 2.15 (br.s, 1H), 2.13 (dd, *J* = 19.9, 1.4, 1H), 2.07 (d, *J* = 13.1 Hz, 1H), 1.94 (d, *J* = 10.5 Hz, 1H), 1.52 (d, *J* = 10.5 Hz, 1H), 1.48 (dddd, *J* = 13.1, 4.0, 3.0, 1.0 Hz, 1H), 1.19 (d, *J* = 7.0 Hz, 3H), 1.14 (s, 3H), 1.00 (s, 3H).

^{13}C NMR (176 MHz, $CDCl_3$) δ 216.7, 92.0, 86.2, 70.6, 63.6, 52.9, 51.1, 47.6, 47.2, 39.6, 33.4, 28.2, 18.5, 10.8, 9.1.

HR-MS (APCI+): m/z calc. for $C_{15}H_{21}O_3^+$ [M+H]$^+$: 249.1485, found: 249.1416.

[a]$^D_{22.0}$ = + 125.5° ($CHCl_3$, c = 1.0). ***T_m*** = 123 °C.

R_f (Et_2O) = 0.8.

The structure was confirmed via X-Ray.

4 NMR-spectra

^{1}H NMR (500 MHz, CDCl$_3$) (**19**)

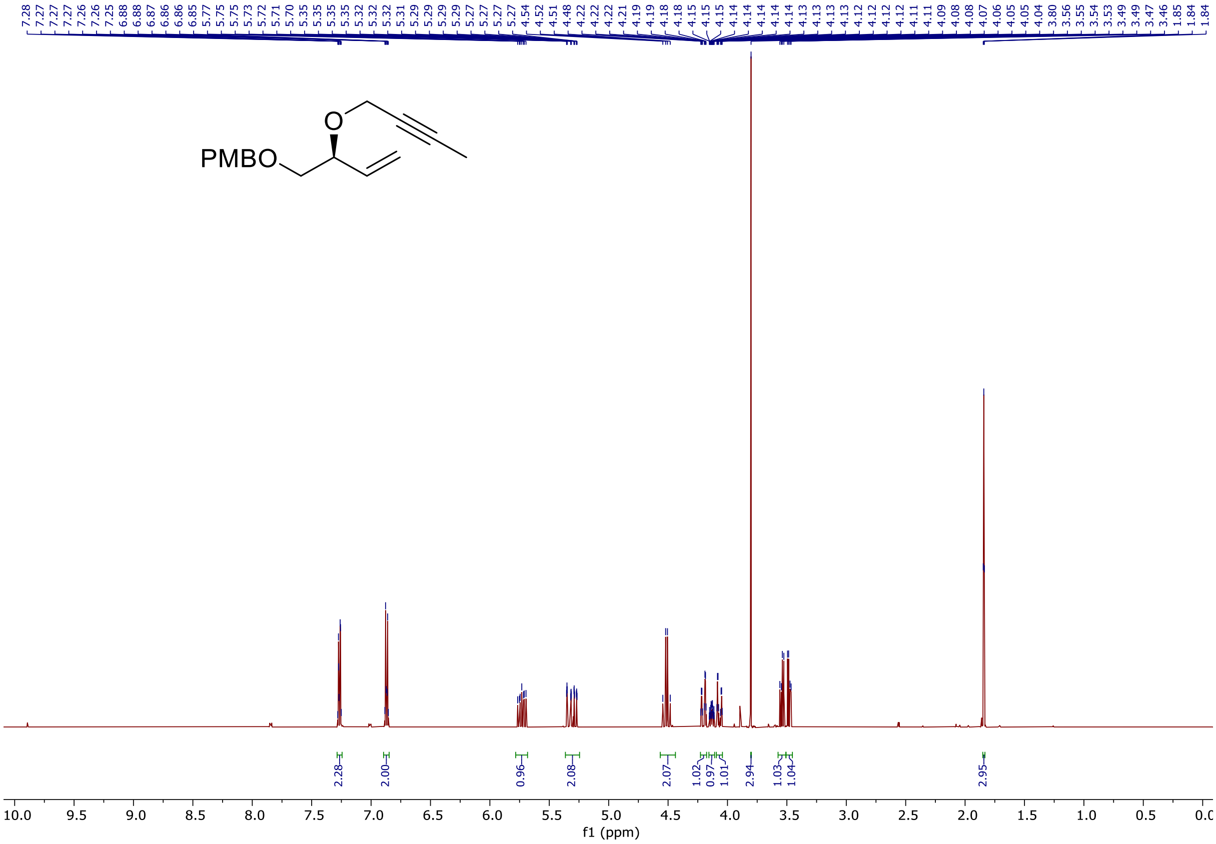

^{13}C NMR (126 MHz, CDCl$_3$)

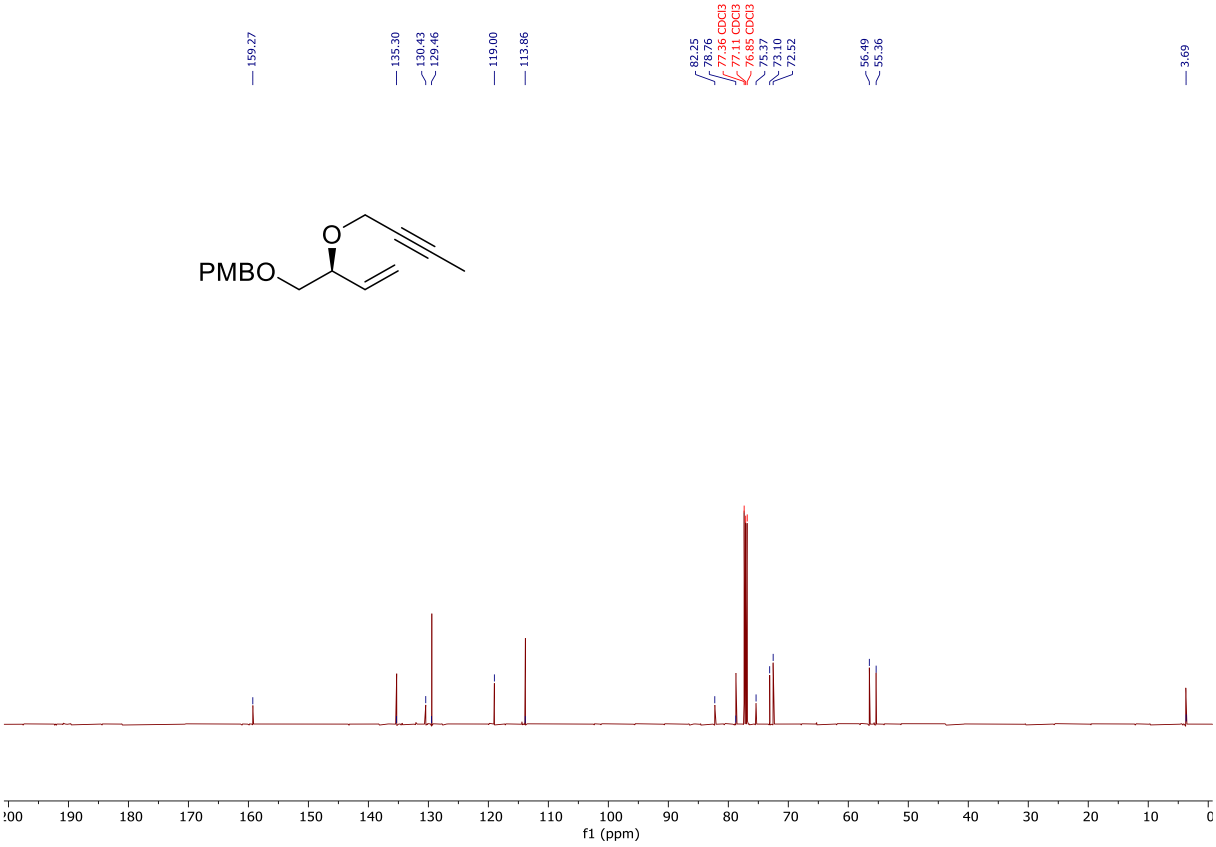

^{1}H NMR (500 MHz, $CDCl_3$) (**20**)

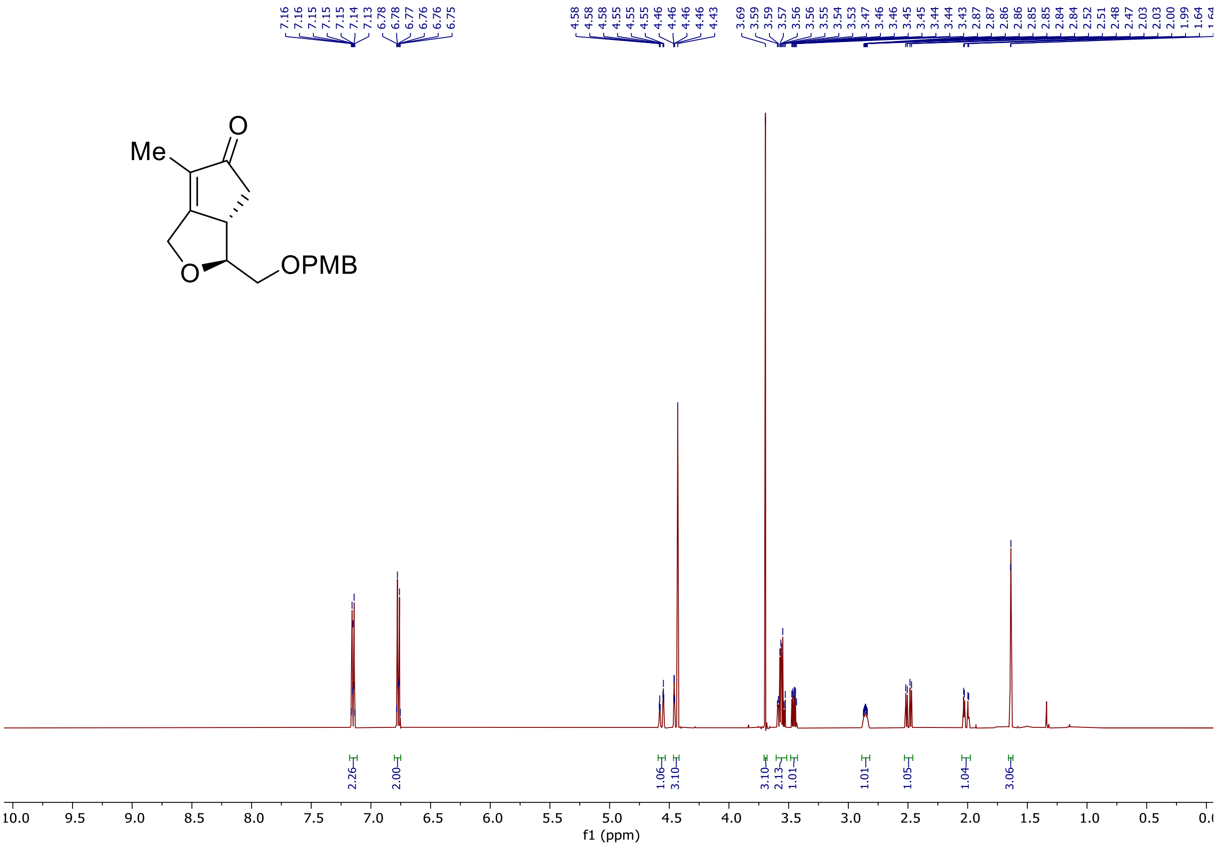

^{13}C NMR (126 MHz, $CDCl_3$)

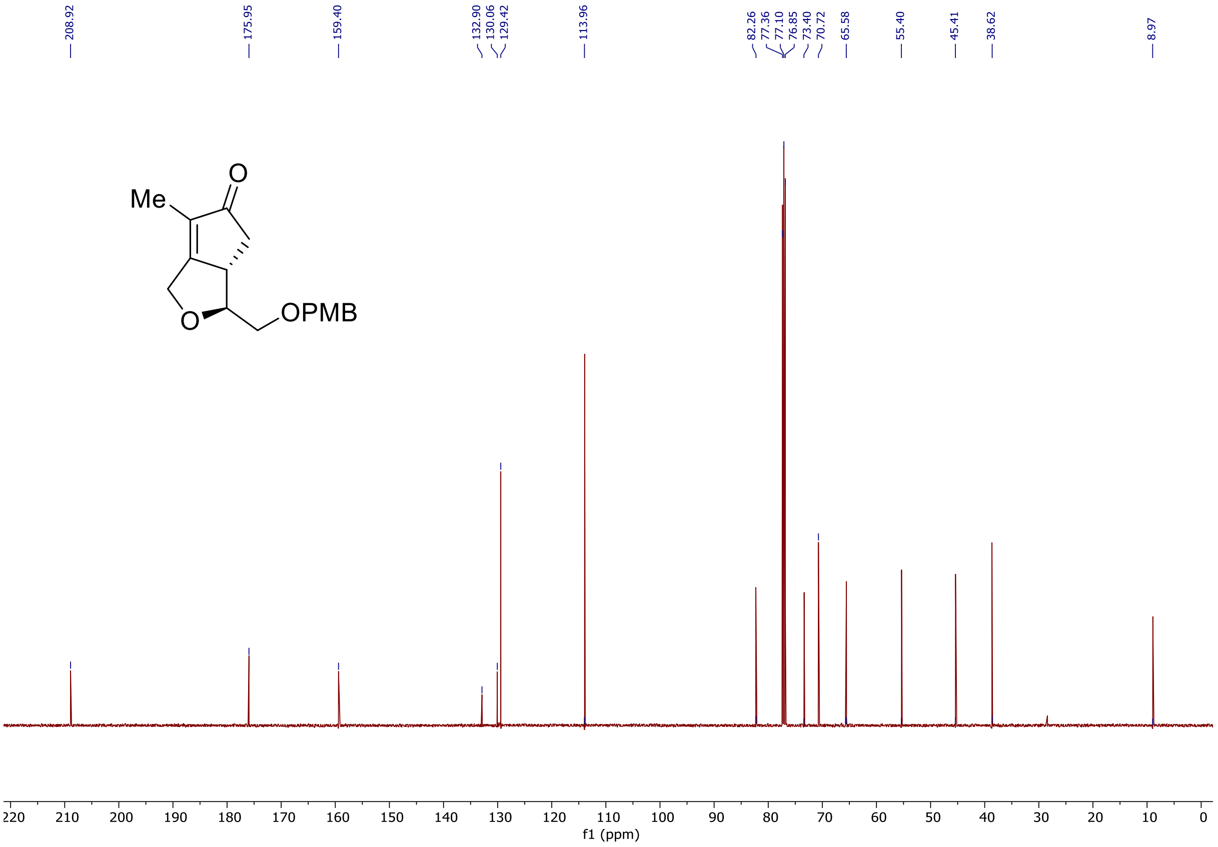

^{1}H NMR (500 MHz, $CDCl_3$) (**20** minor)

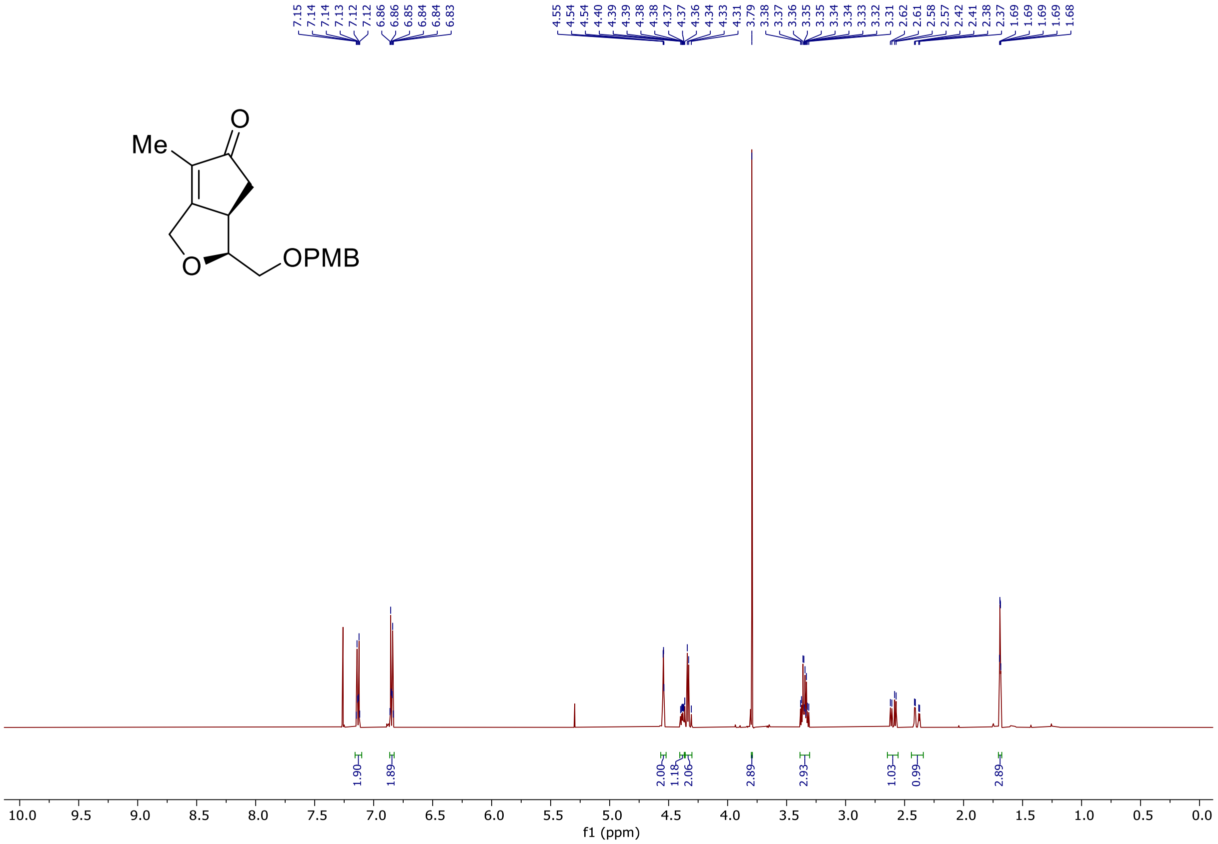

^{13}C NMR (126 MHz, $CDCl_3$)

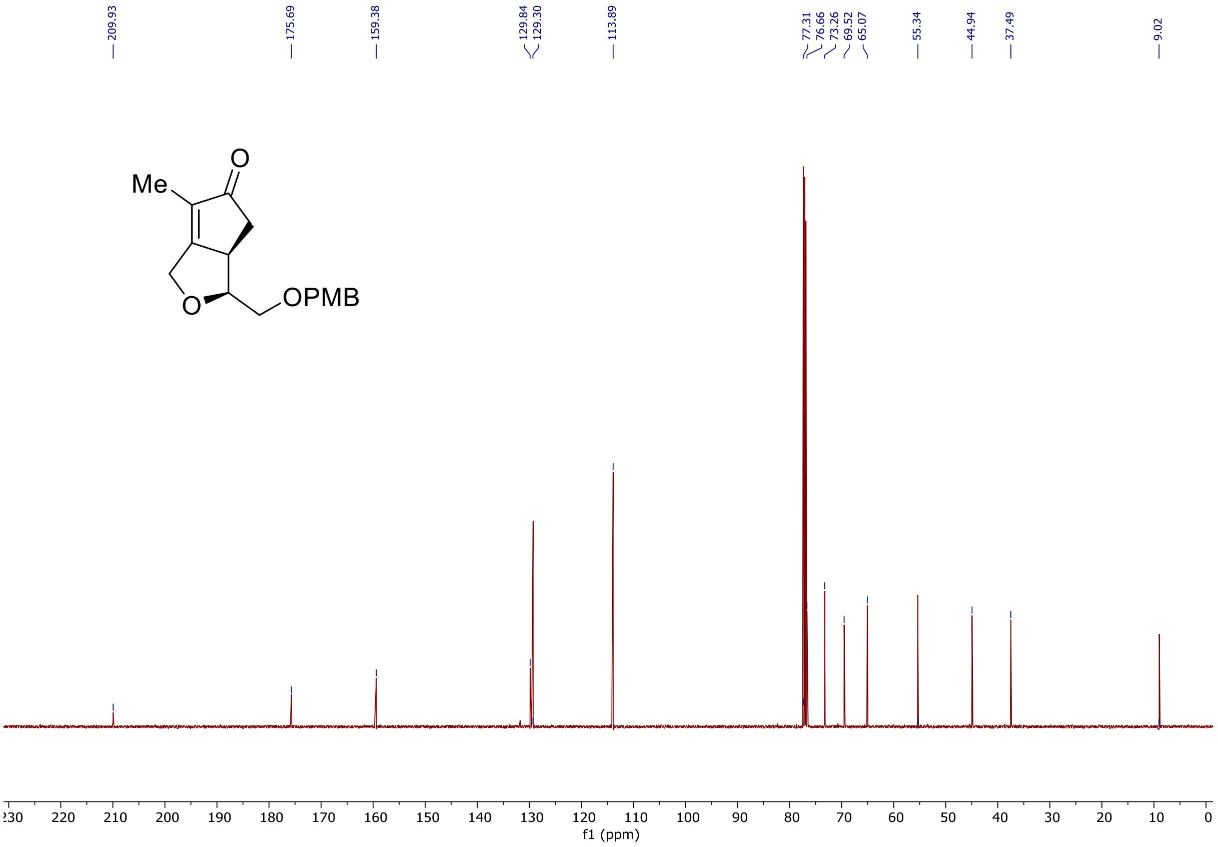

^{1}H NMR (500 MHz, $CDCl_3$) (**21a**)

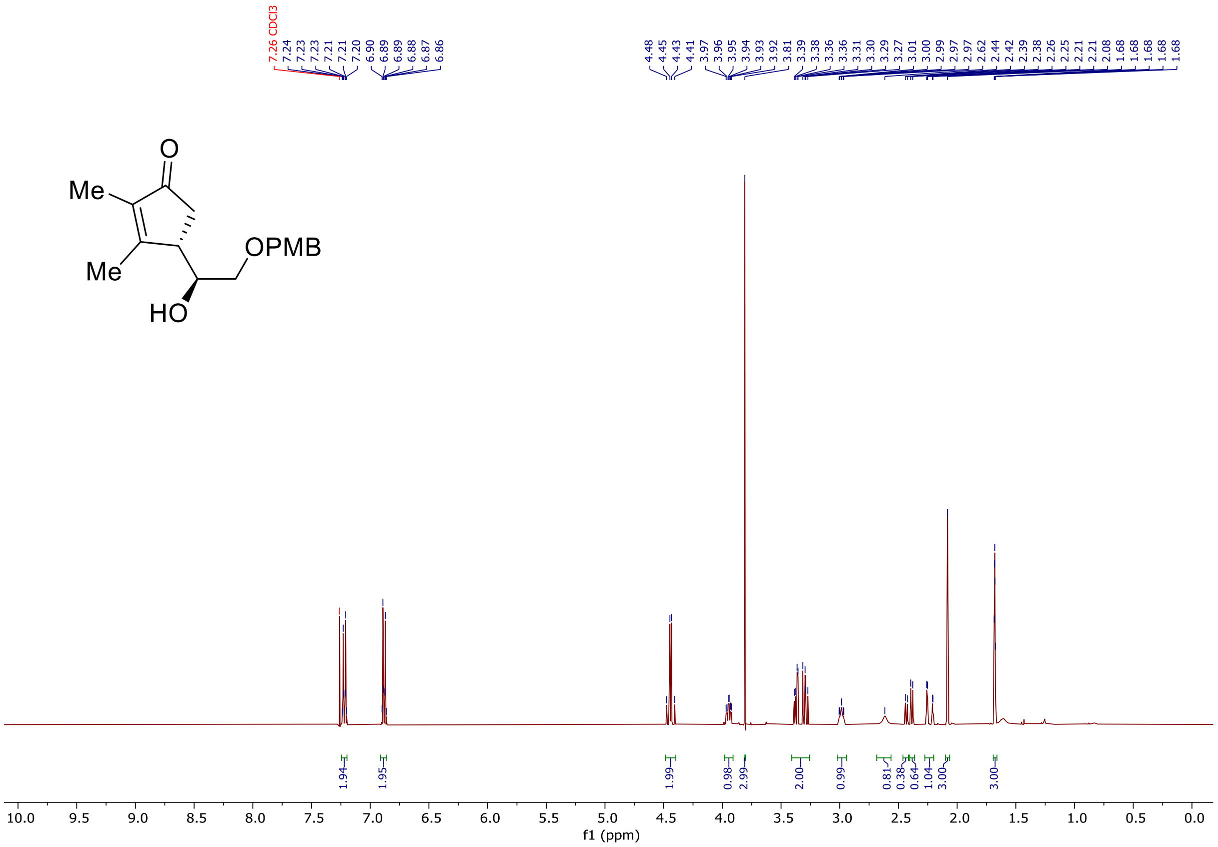

^{13}C NMR (126 MHz, $CDCl_3$)

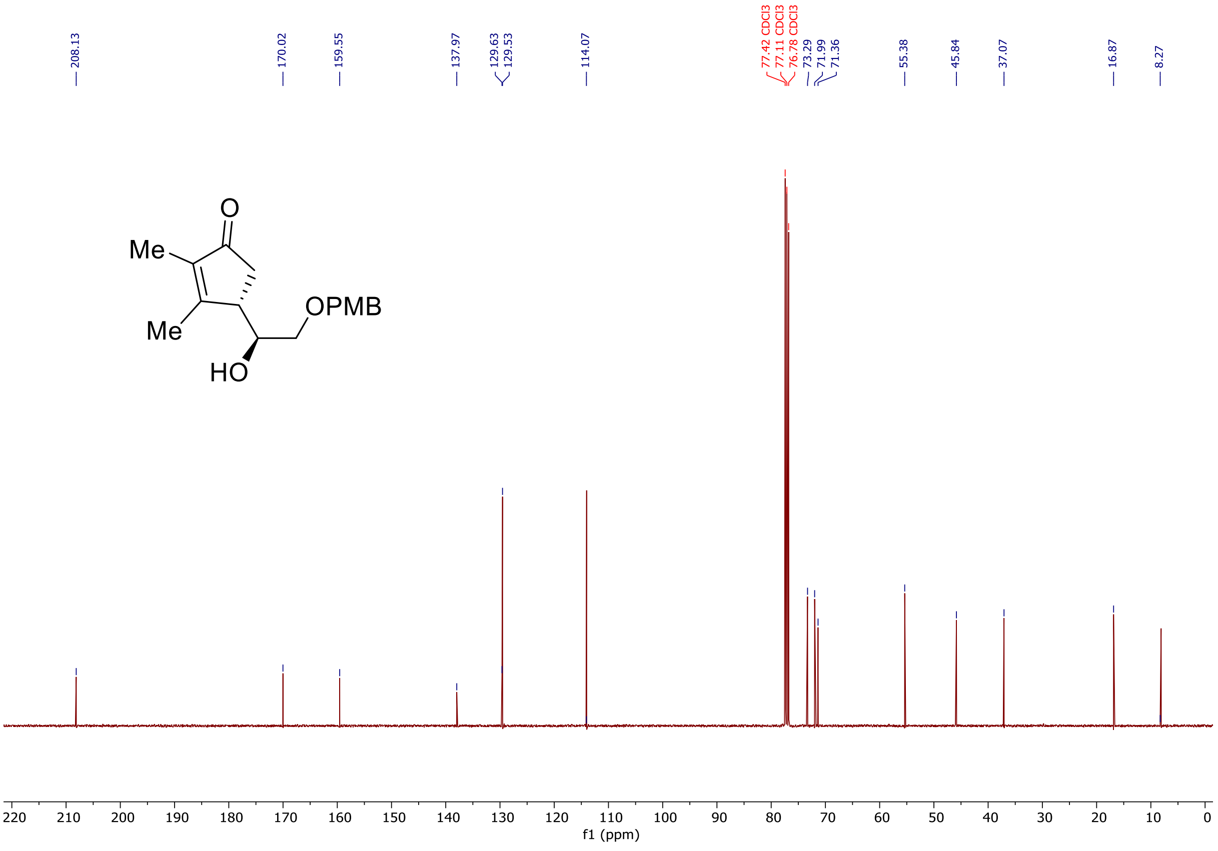

^{1}H NMR (400 MHz, CDCl$_3$) (**21b**)

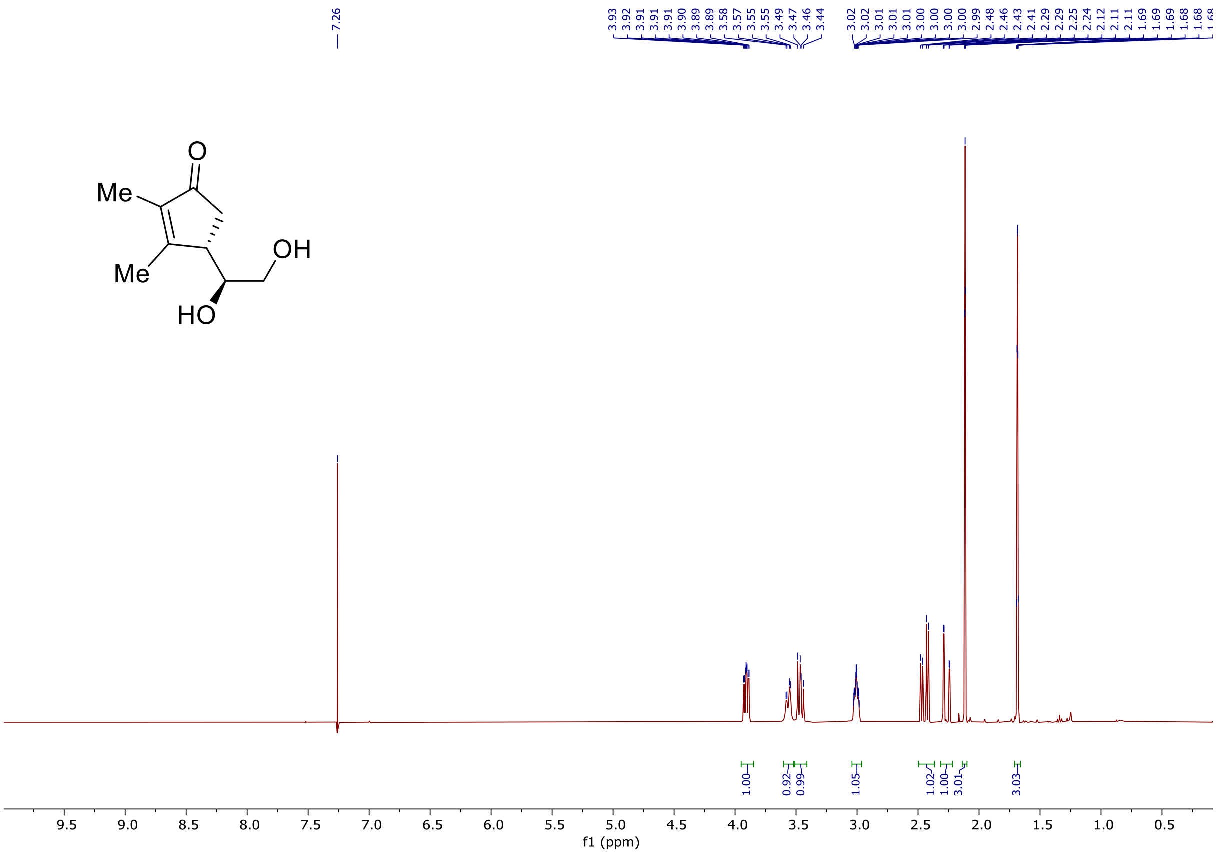

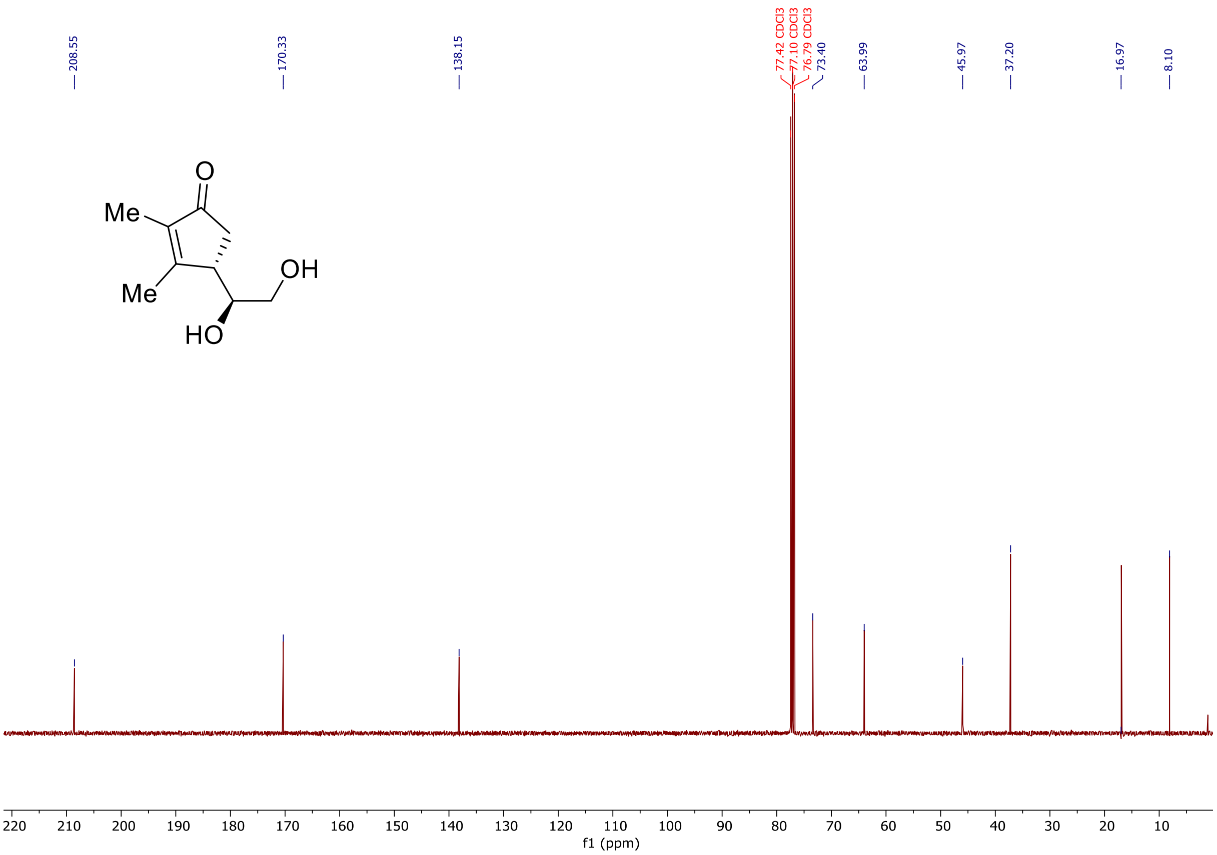

^{1}H NMR (500 MHz, $CDCl_3$) (**21c**)

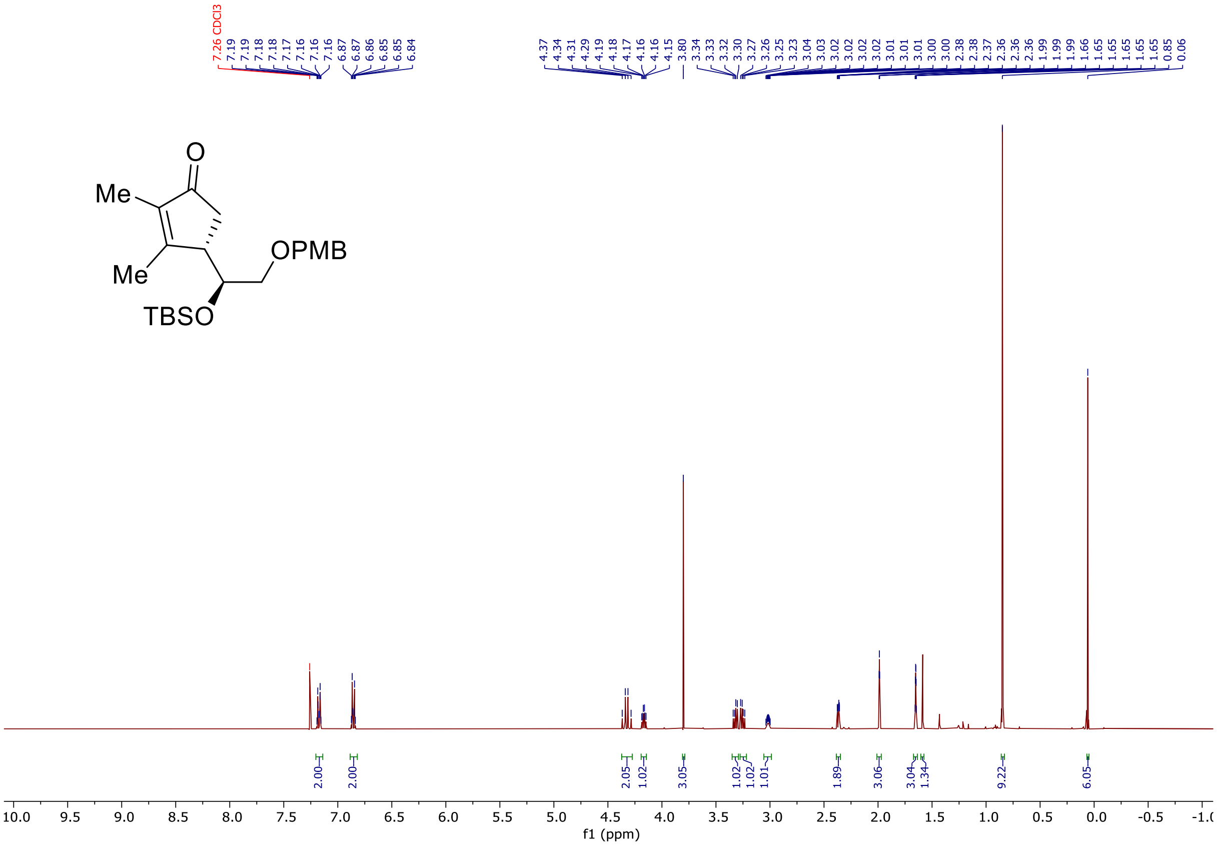

^{13}C NMR (126 MHz, $CDCl_3$)

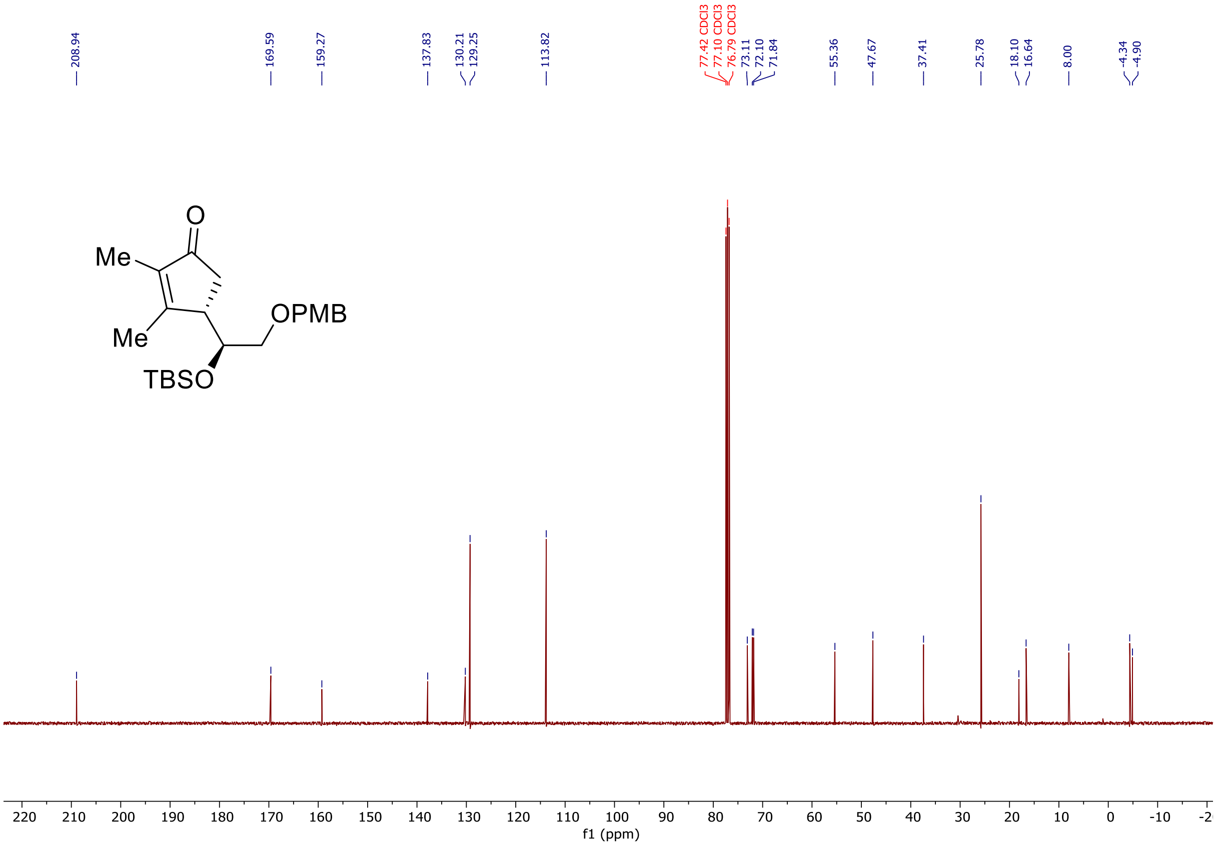

^1H NMR (700 MHz, $CDCl_3$) (**ent-22c**)

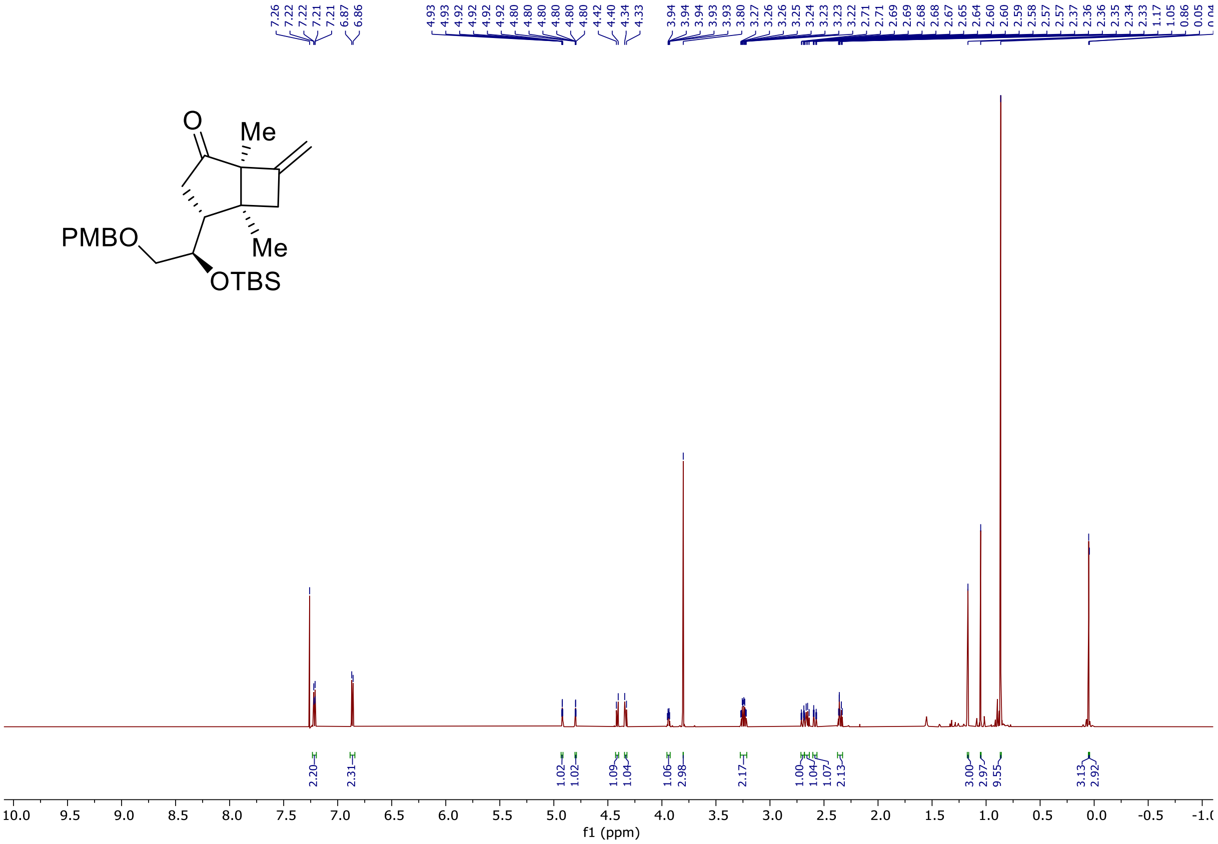

^{13}C NMR (176 MHz, $CDCl_3$)

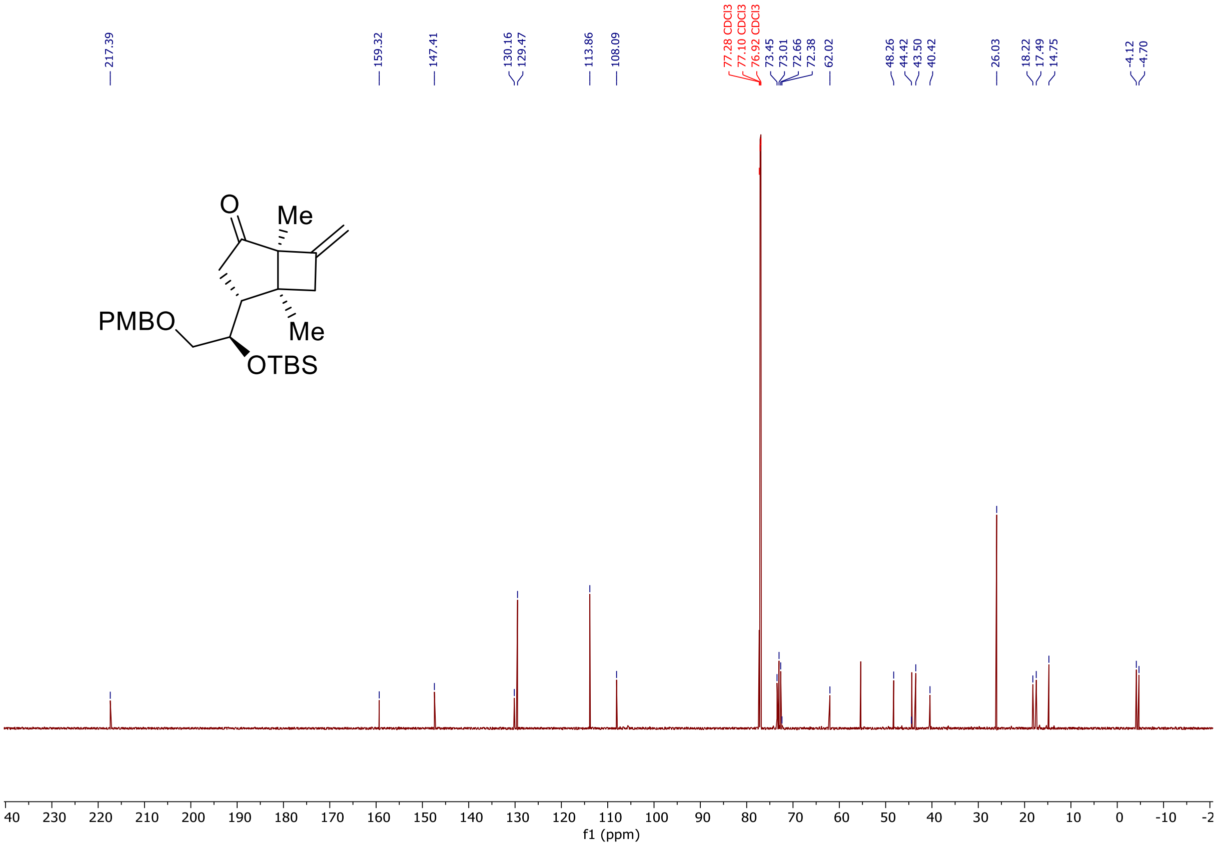

^{1}H NMR (700 MHz, $CDCl_3$) (**ent-22b**)

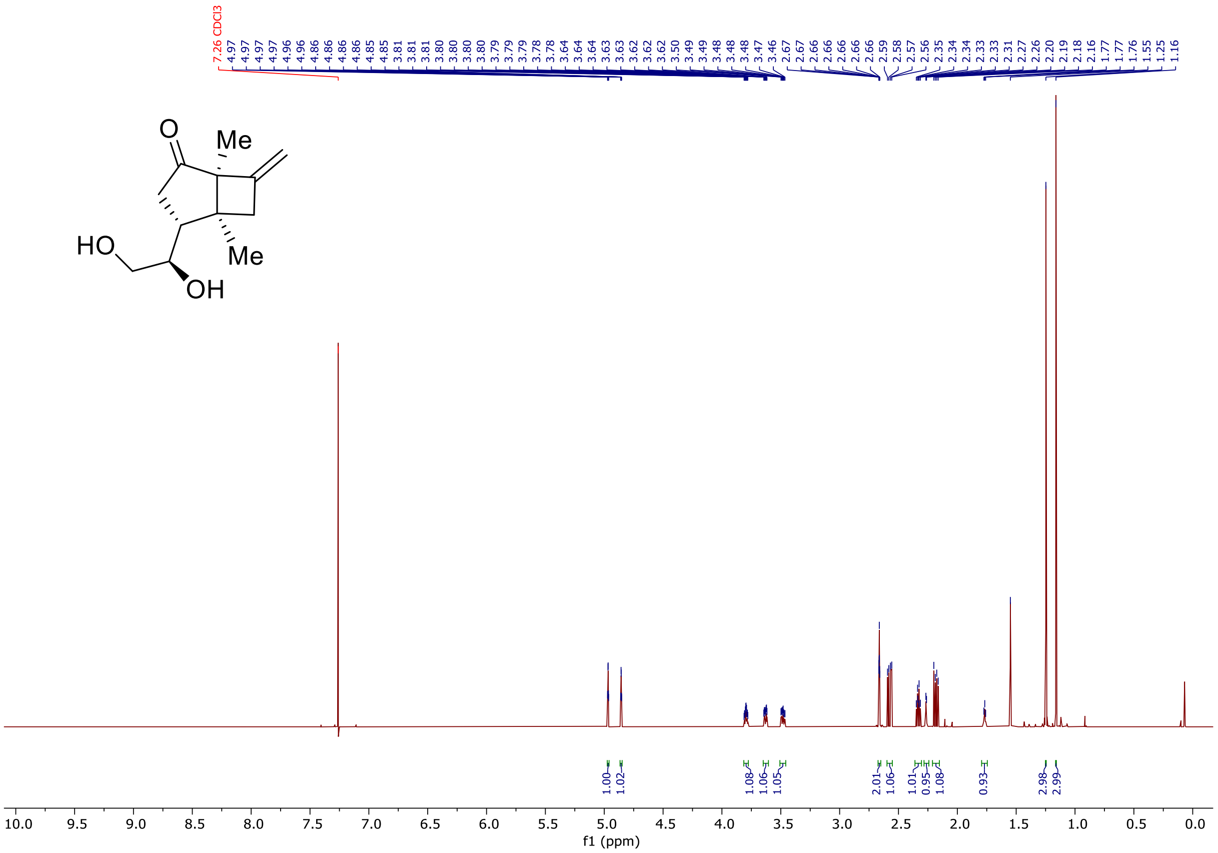

^{13}C NMR (176 MHz, $CDCl_3$)

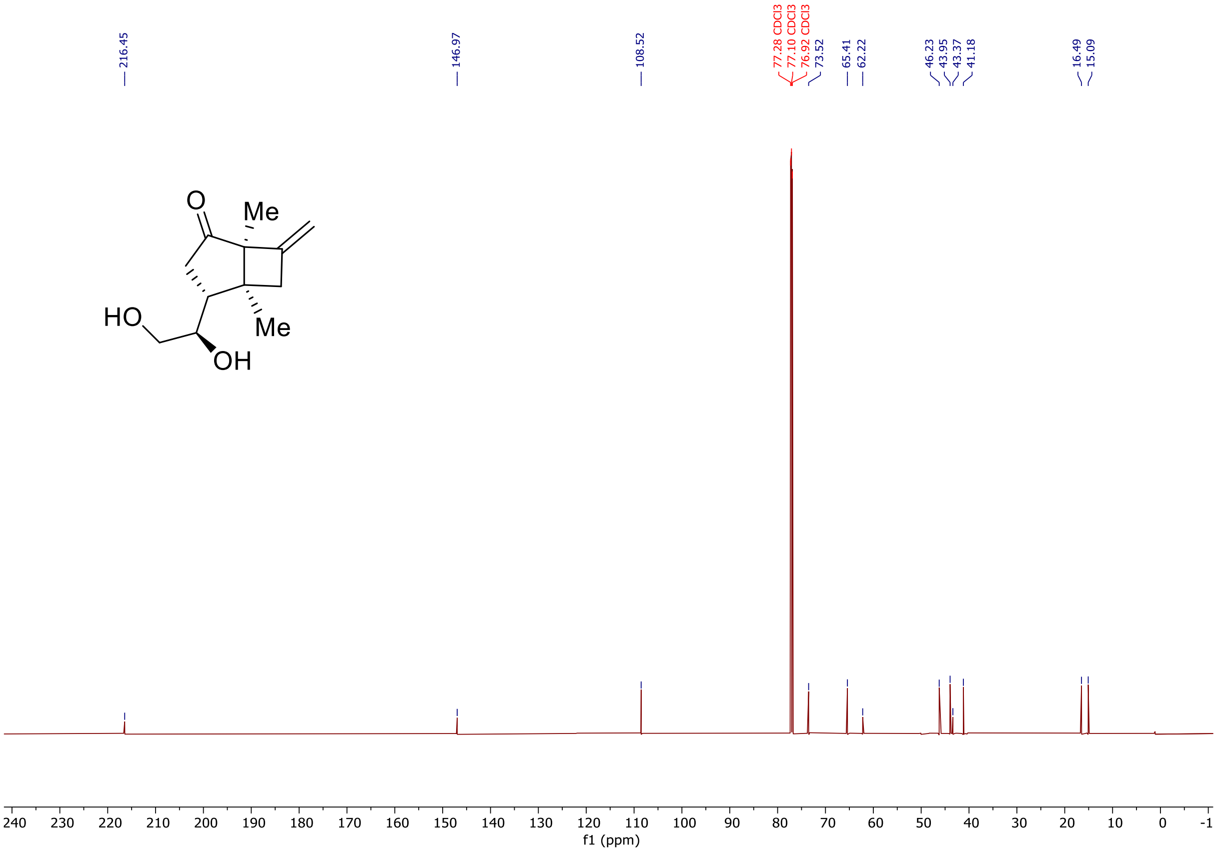

^{1}H NMR (700 MHz, C_6D_6) (**24b**)

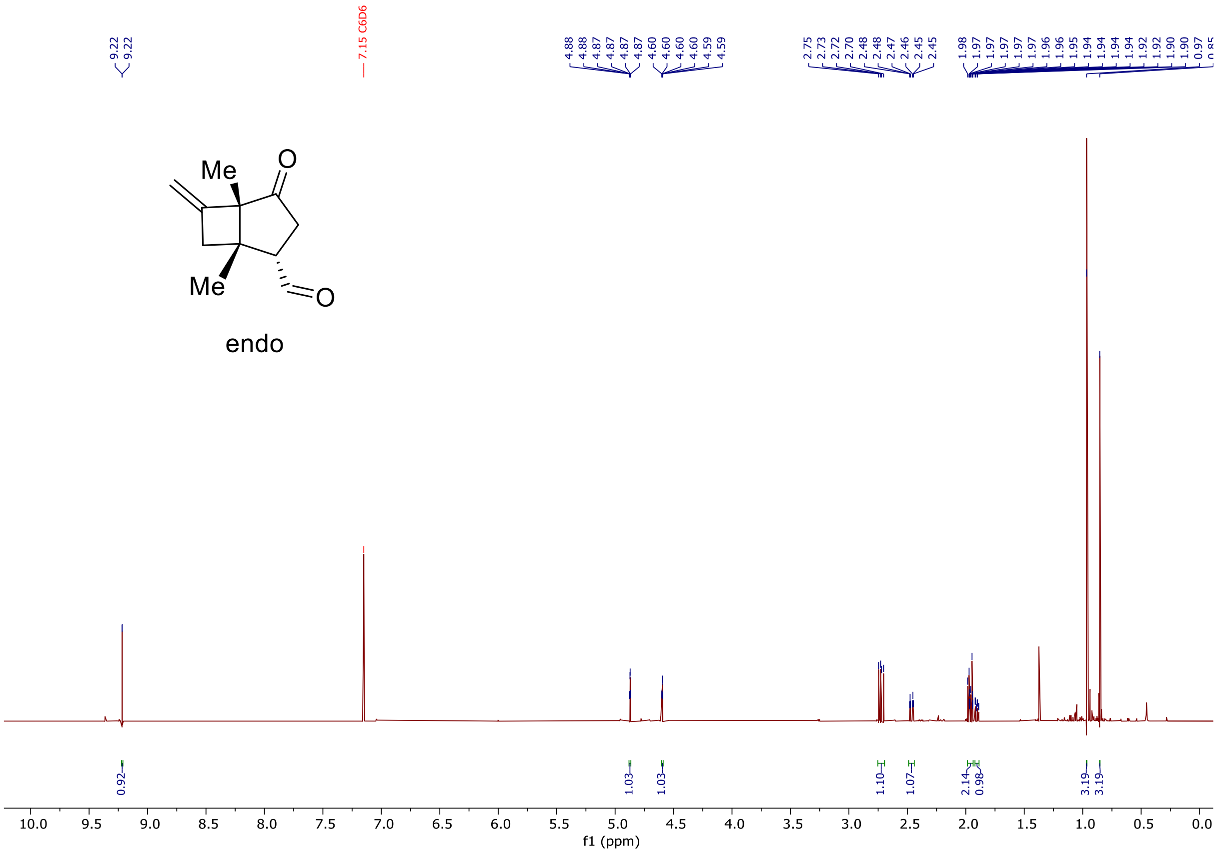

^{13}C NMR (101 MHz, C_6D_6)

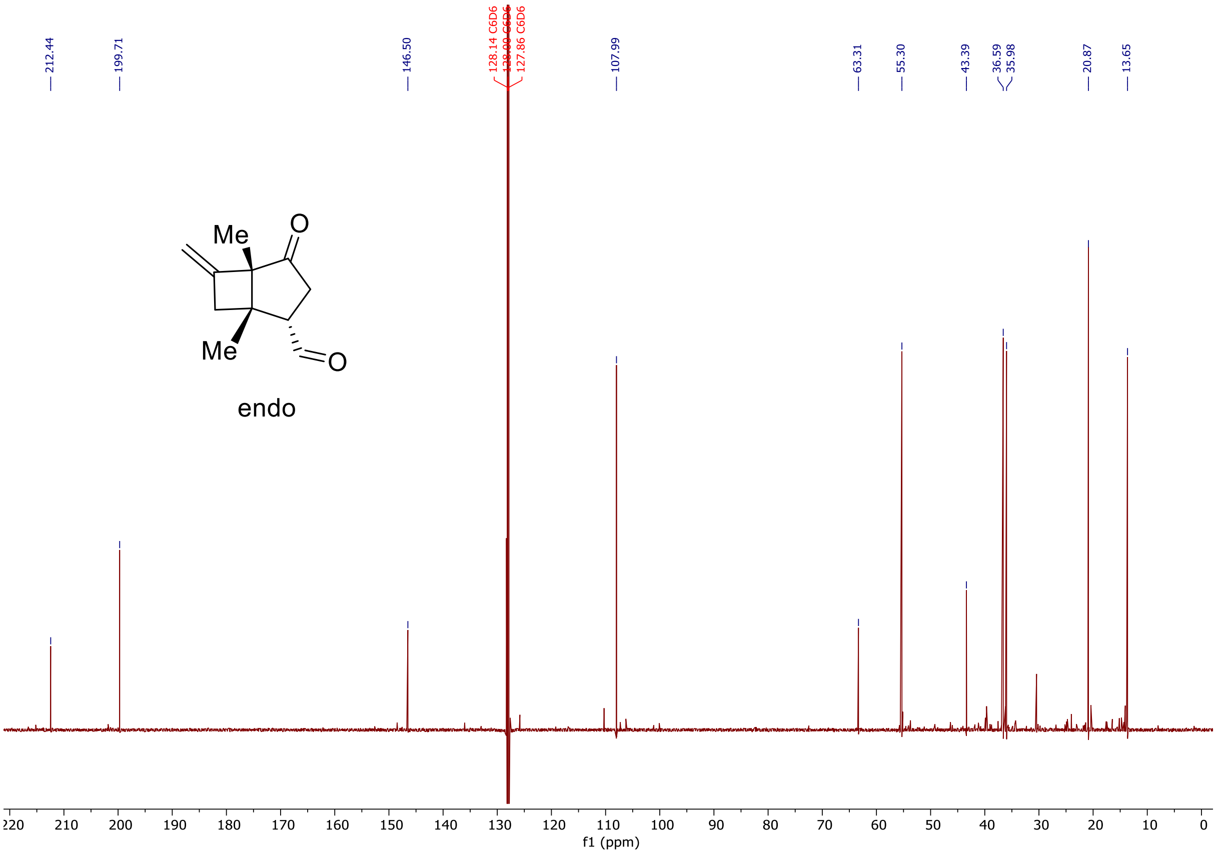

^{1}H NMR (700 MHz, C_6D_6) **(ent-24a)**

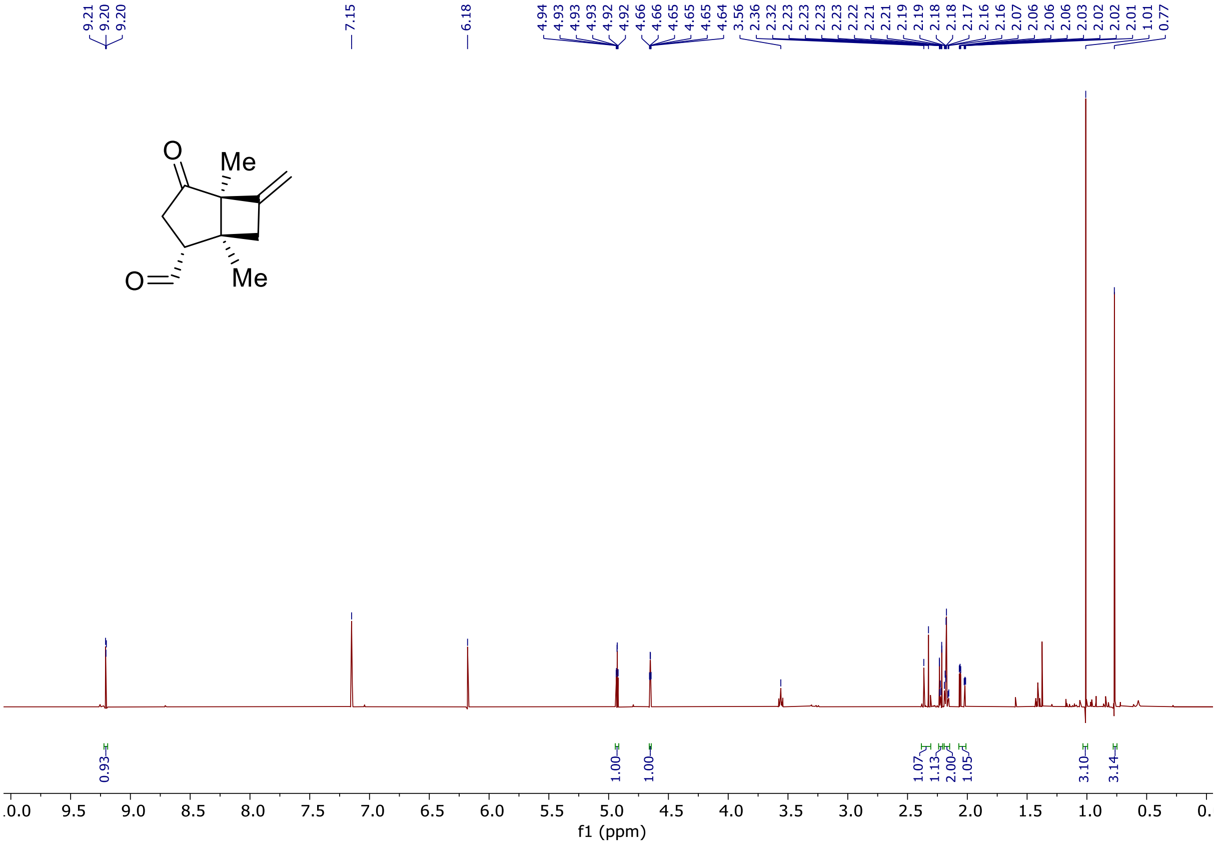

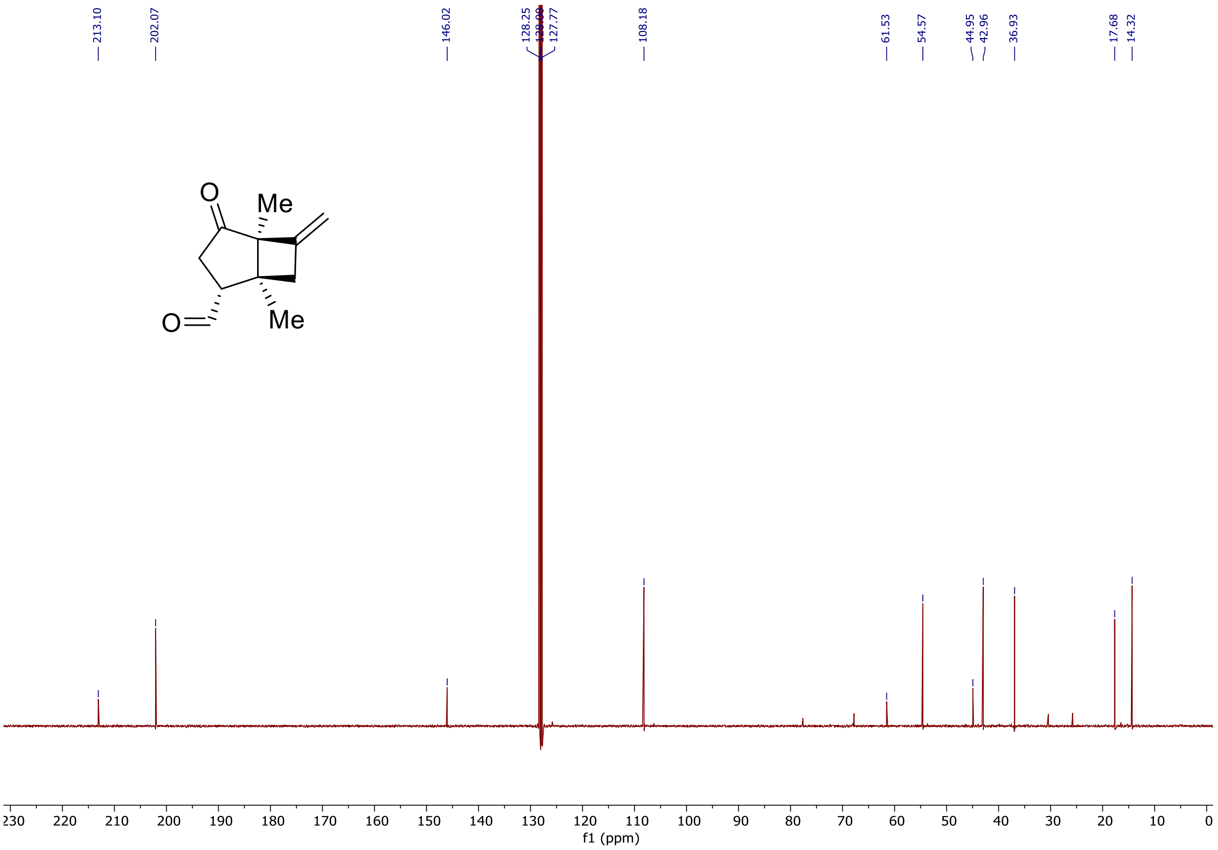

^{1}H NMR (700 MHz, $CDCl_3$) **(25)**

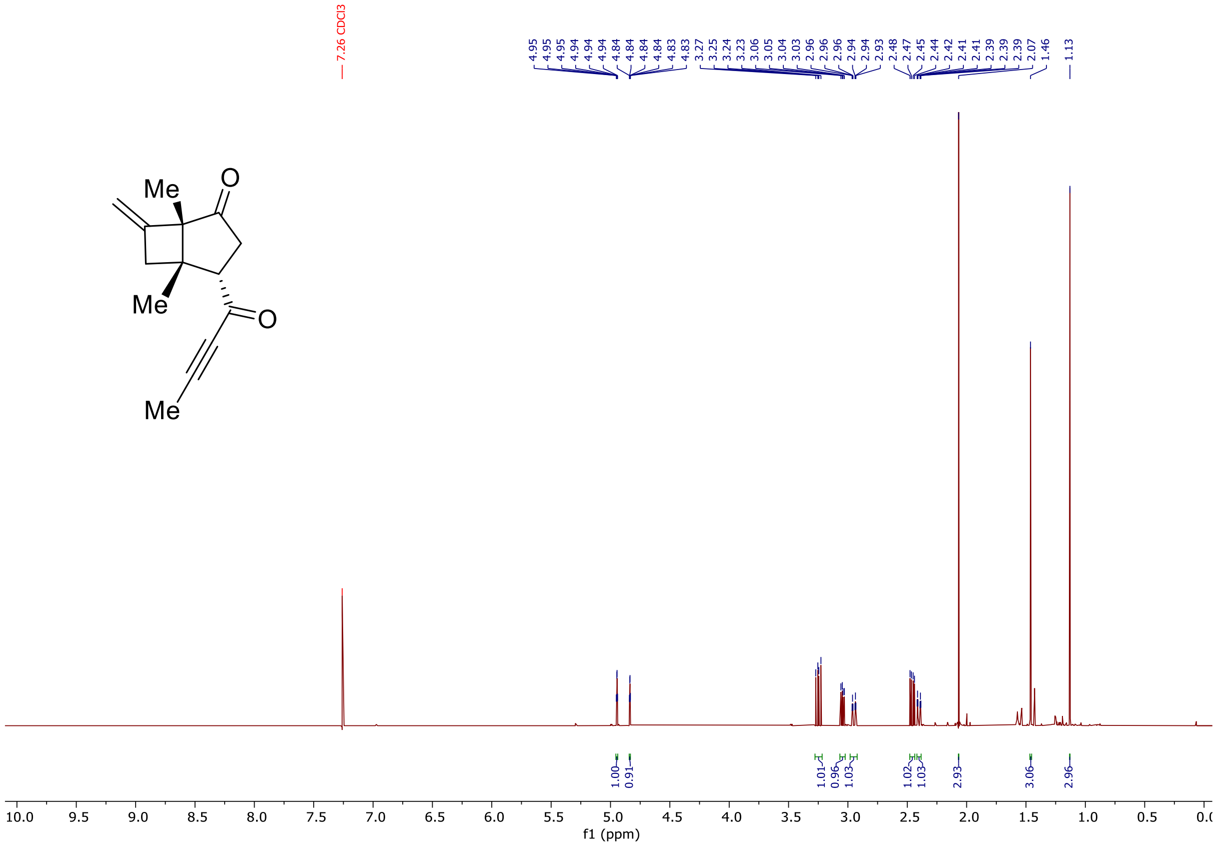

^{13}C NMR (176 MHz, $CDCl_3$) **(25)**

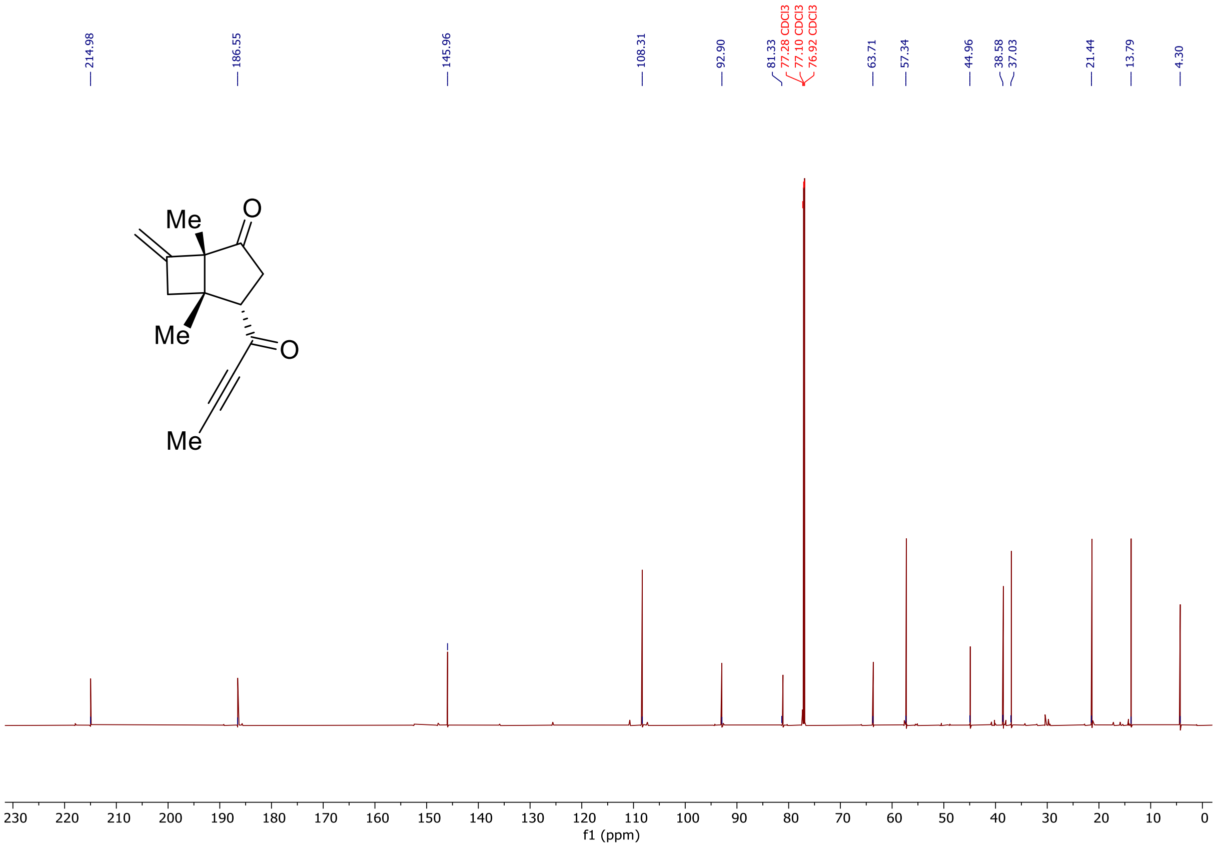

^{1}H NMR (700 MHz, C_6D_6) **(27)**

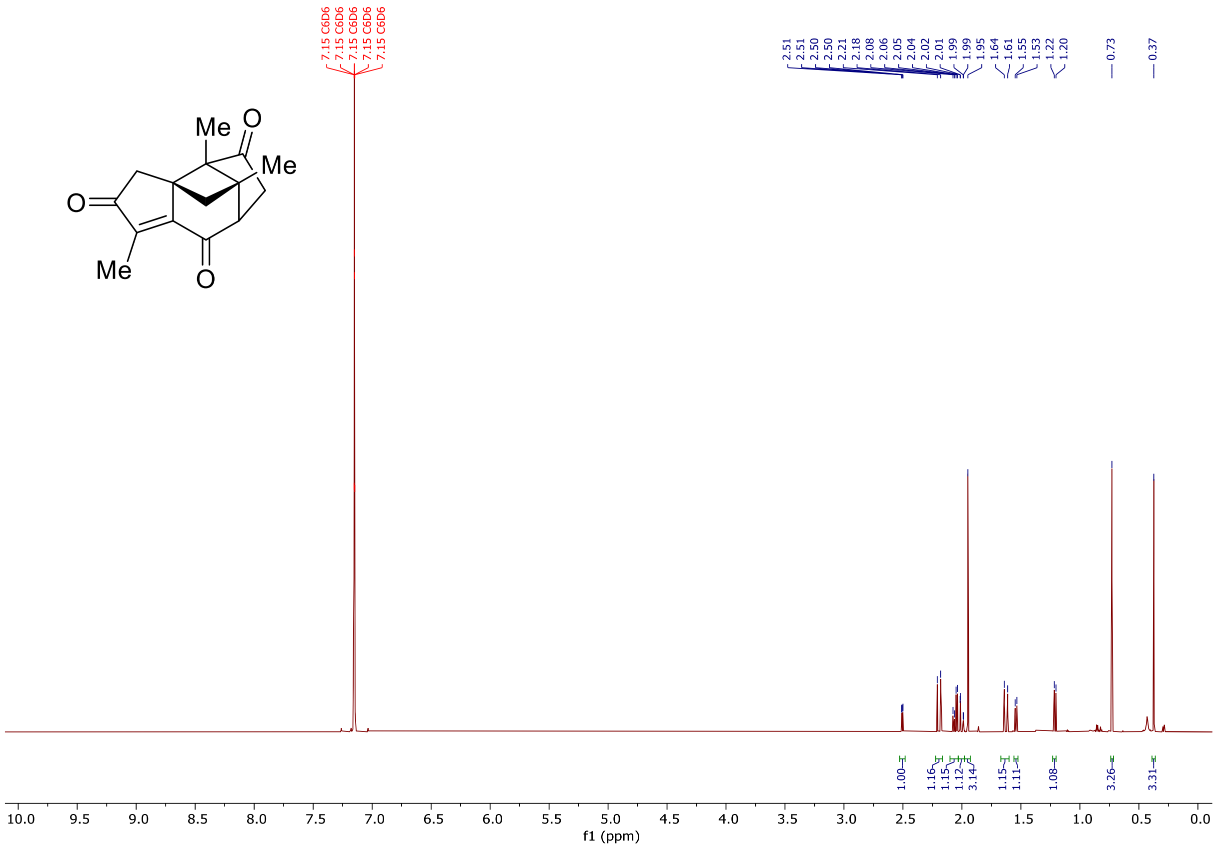

^{1}H NMR (700 MHz, C_6D_6) zoomed **(27)**

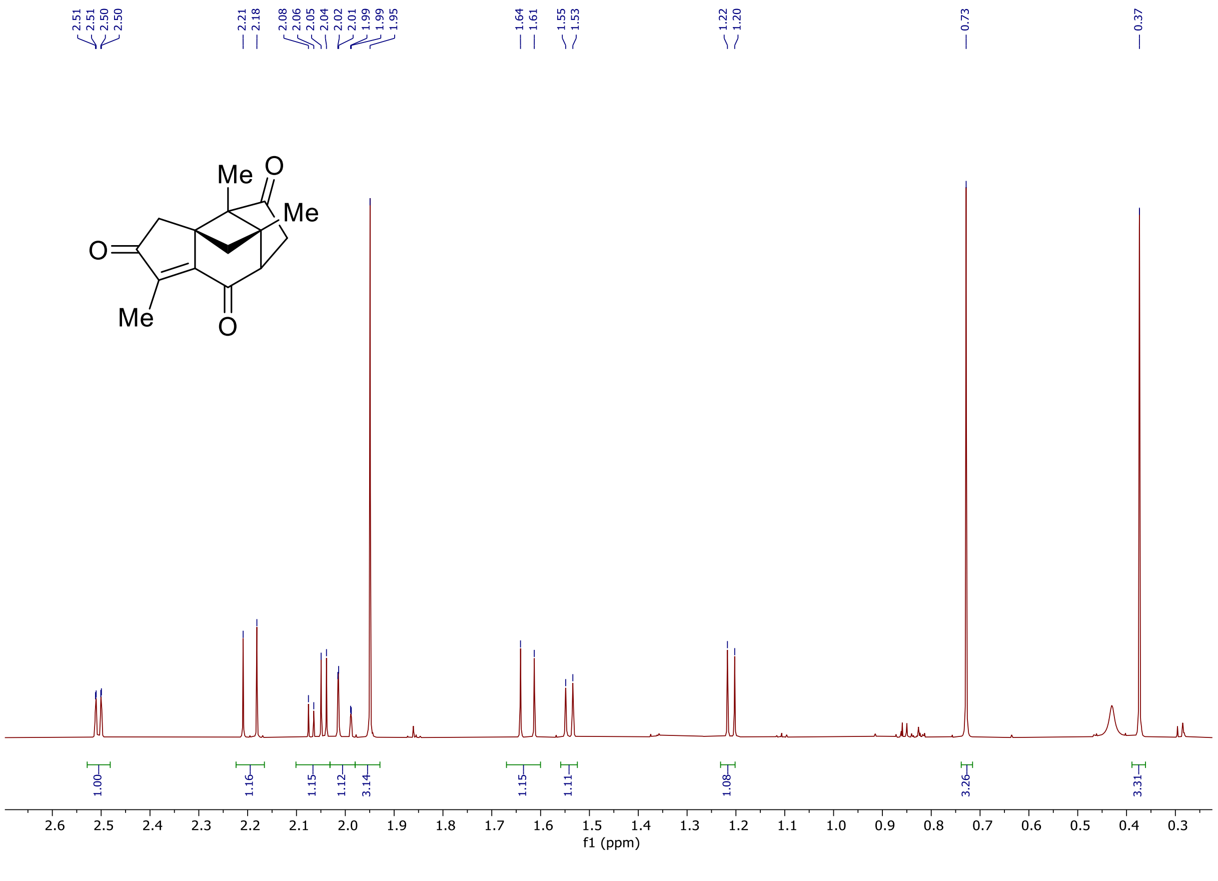

^{13}C NMR (176 MHz, C_6D_6) **(27)**

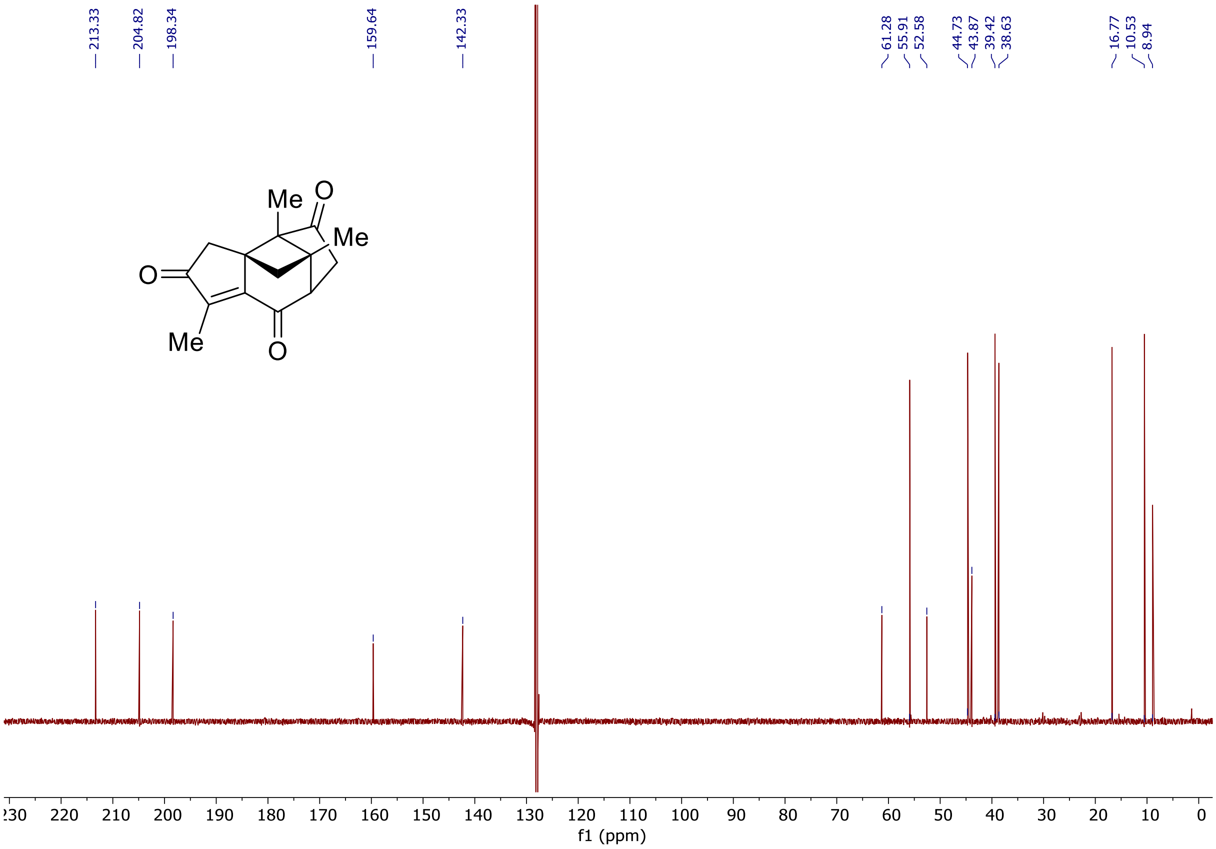

^{1}H NMR (700 MHz, $CDCl_3$) **(2)**

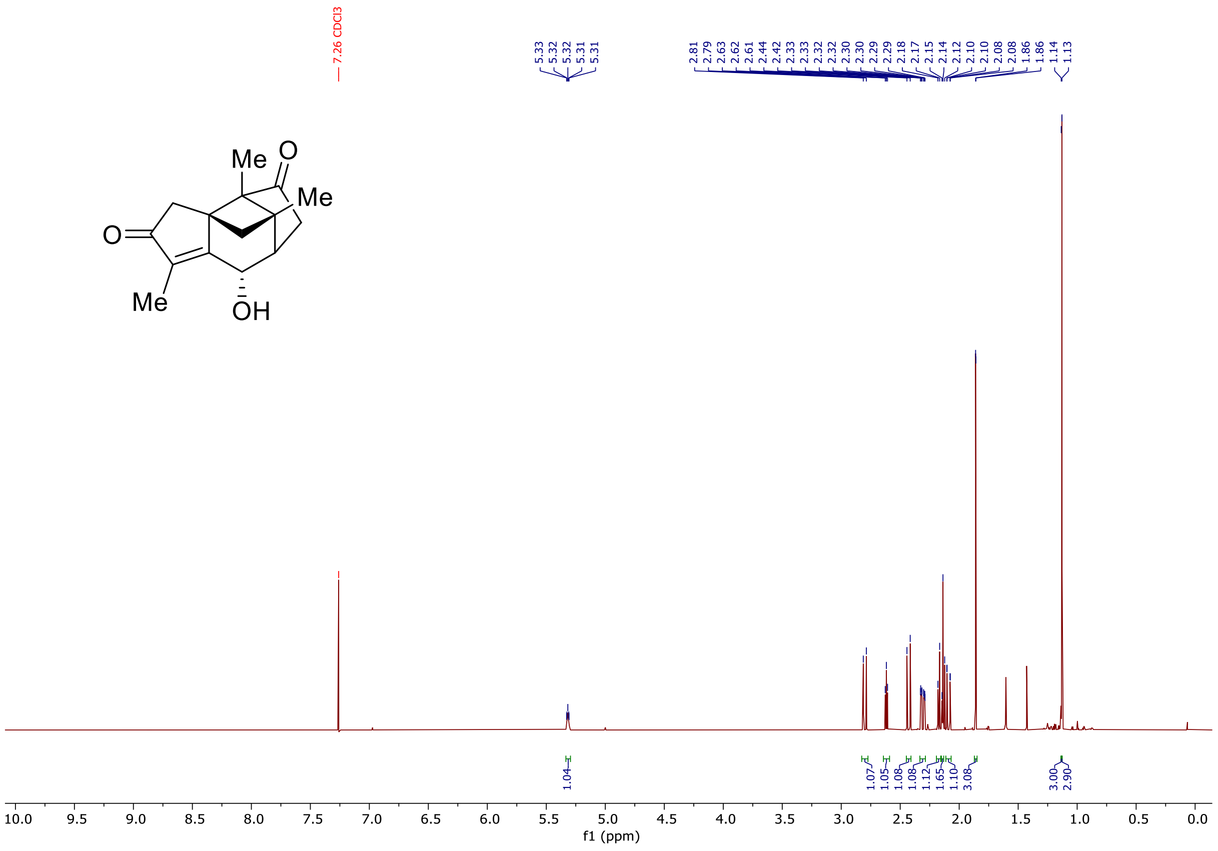

^{1}H NMR (700 MHz, $CDCl_3$) **(2)** zoomed

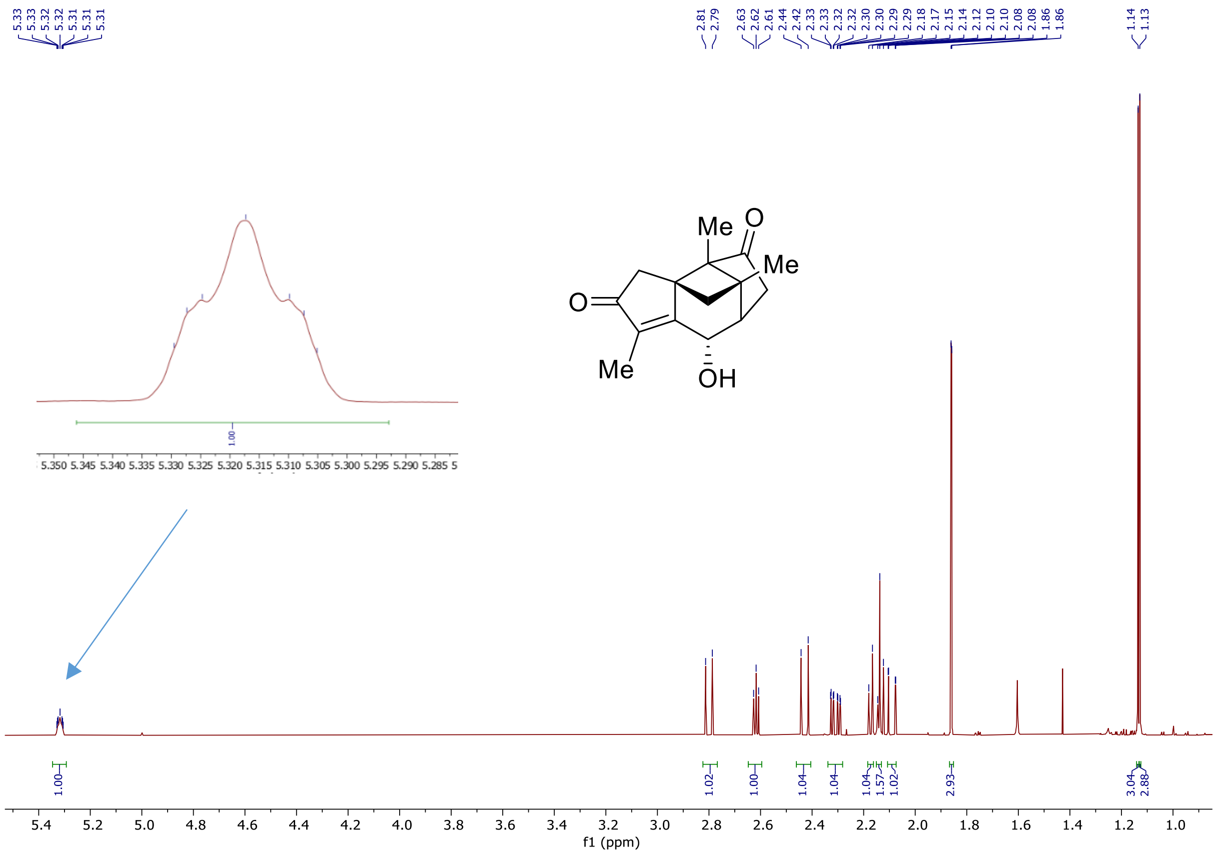

^{13}C NMR (176 MHz, $CDCl_3$) **(2)**

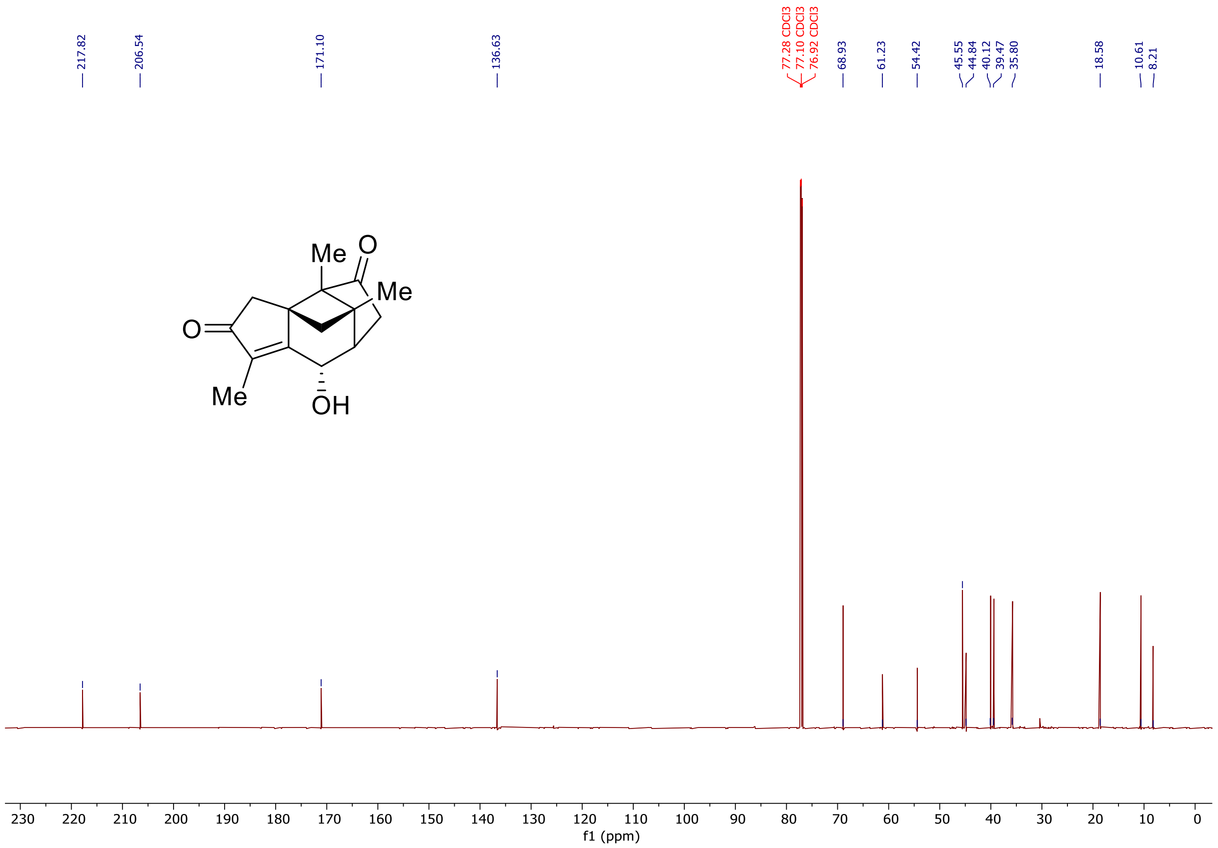

Comparison with data from first isolation[11]

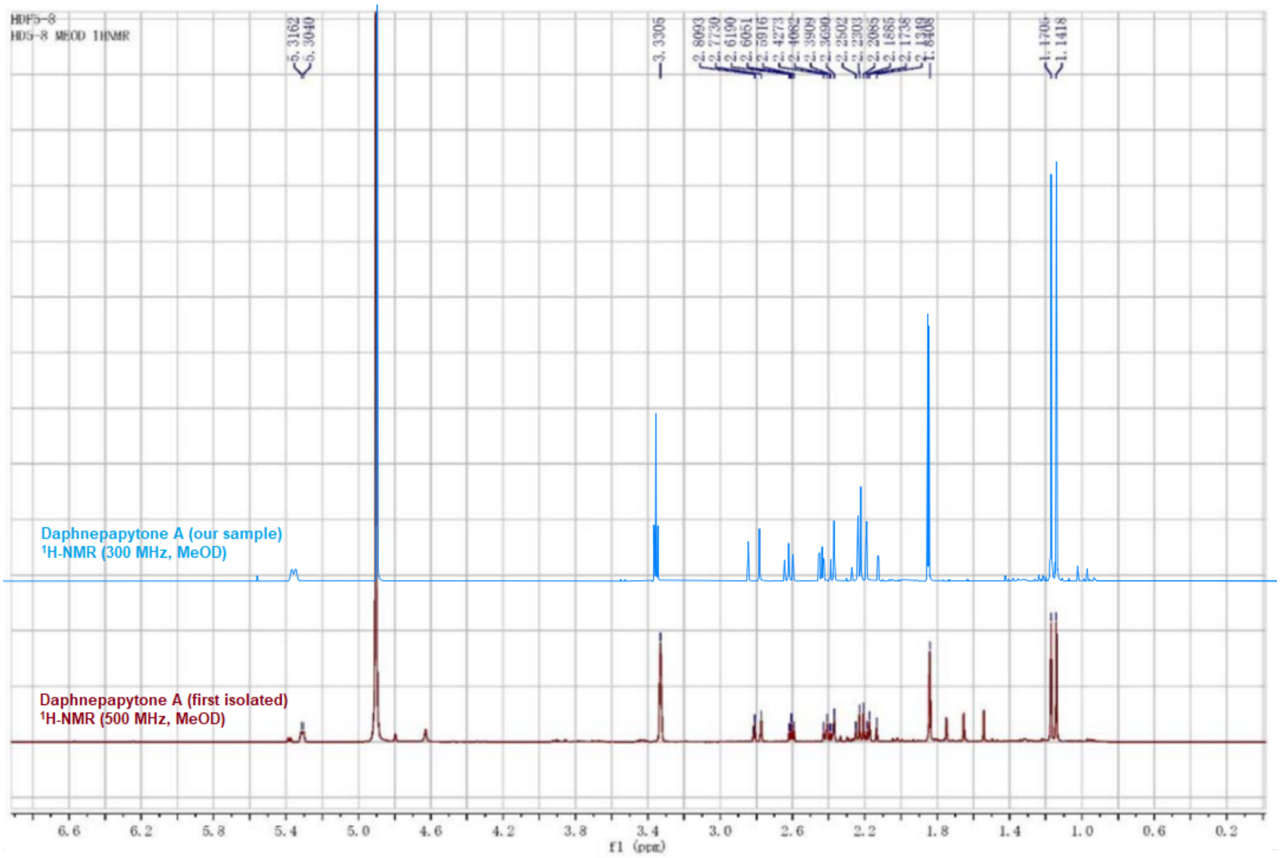

^{1}H NMR (700 MHz, $CDCl_3$) **(28)**

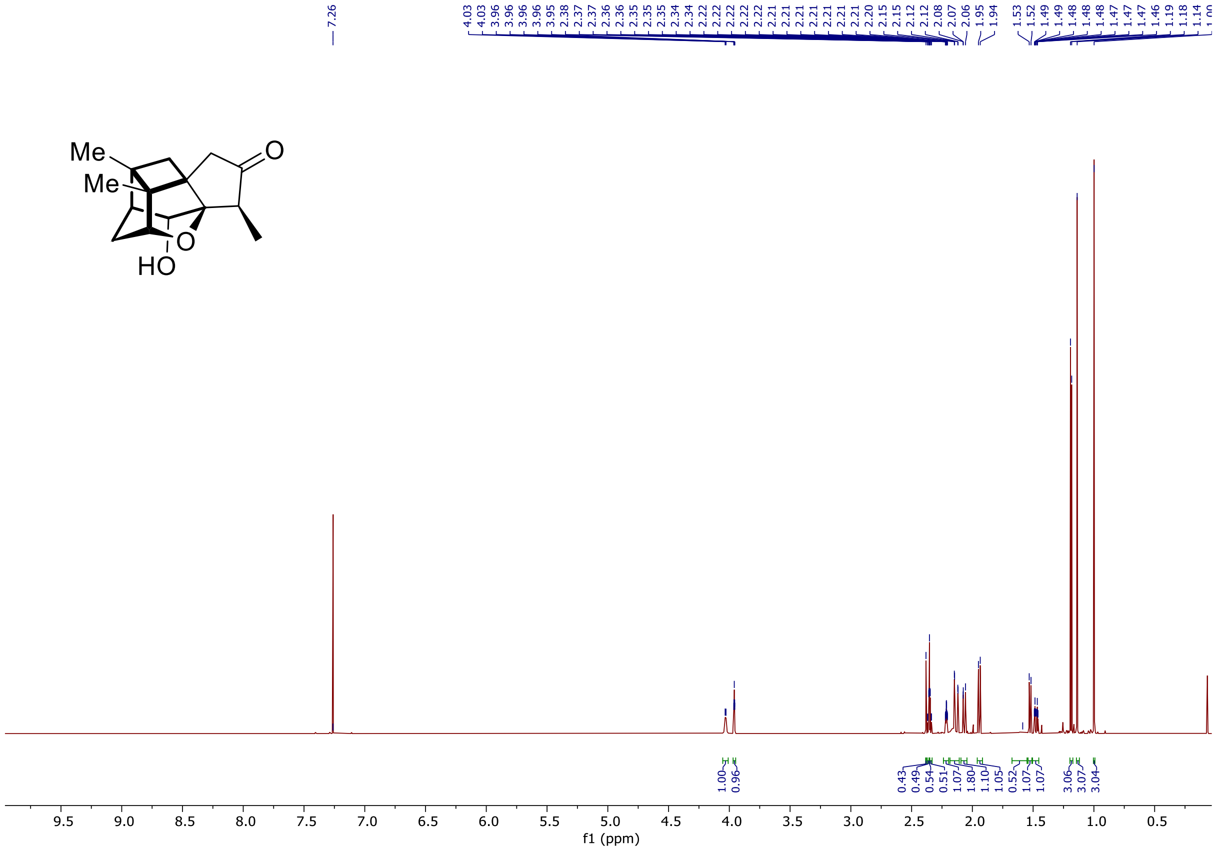

^{13}C NMR (176 MHz, $CDCl_3$) **(28)**

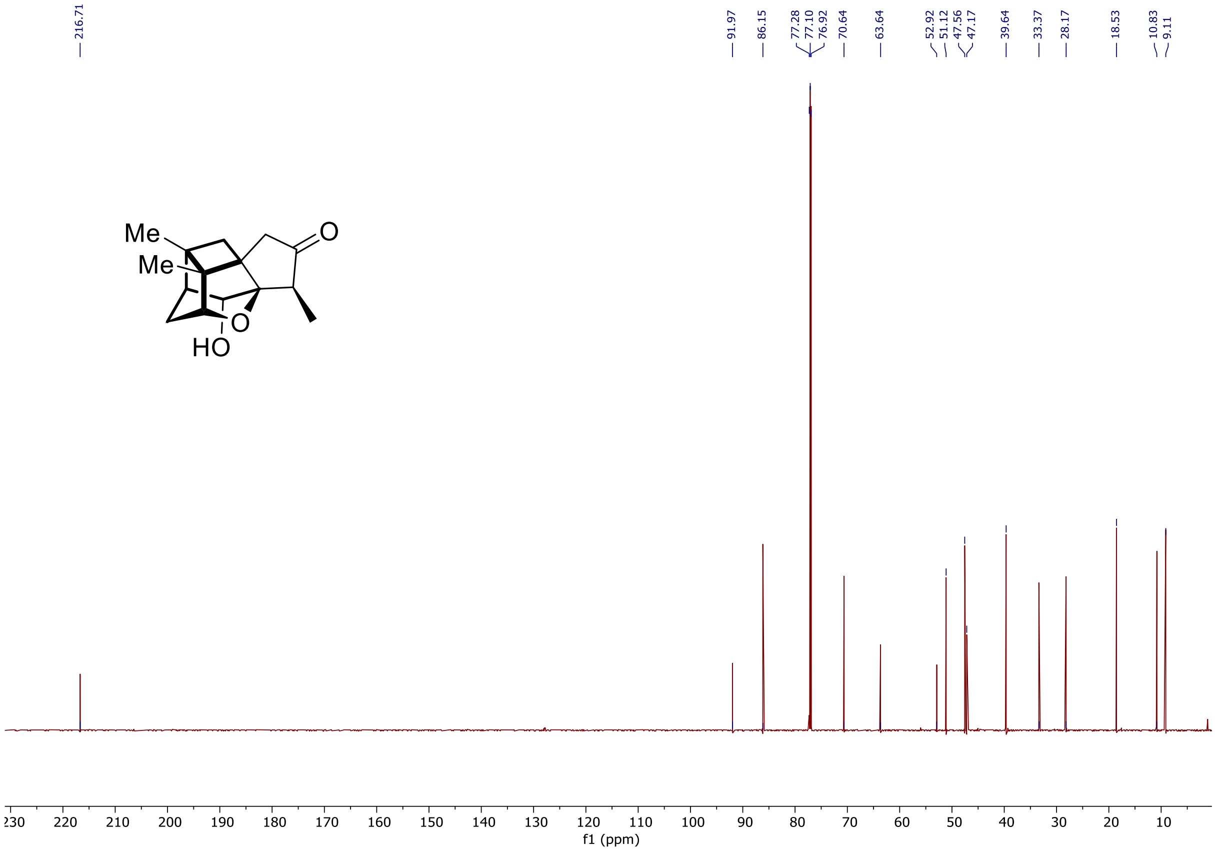

5 Crystal structure data

Compound **ent-22b**

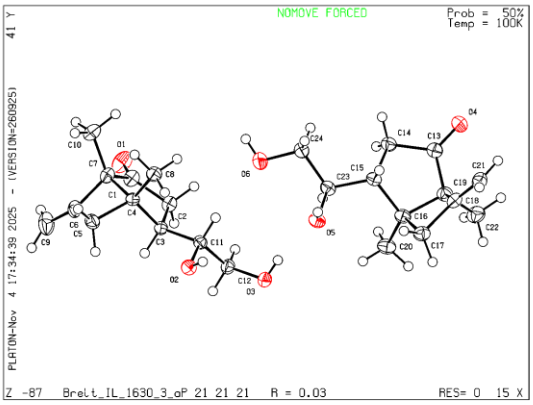

Crystals were obtained at room temperature by slow solvent evaporation from asolution of the compound dissolved in ethylacetate. A colourless, needle-shaped crystal was mounted on a MiTeGen micromount with perfluoroether oil. Data for Breit_IL_1630_3_a were collected from a shock-cooled single crystal at 100(2) K on a Bruker D8 VENTURE dual wavelength Mo/Cu three-circle diffractometer with a microfocus sealed X-ray tube using a mirror optics as monochromator and a Bruker PHOTON III detector. The diffractometer was equipped with an Oxford Cryostream 800 low temperature device and used CuK_α radiation (λ = 1.54178 Å). All data were integrated with SAINT V8.42 and a multi-scan absorption correction using SADABS 2016/2 was applied.[12,13] The structure was solved by direct methods with SHELXT and refined by full-matrix least-squares methods against F^2 using SHELXL-2019/2.[14,15] All non-hydrogen atoms were refined with anisotropic displacement parameters. All C-bound hydrogen atoms were refined isotropic on calculated positions using a riding model with their U_{iso} values constrained to 1.5 times the U_{eq} of their pivot atoms for terminal sp^3 carbon atoms and 1.2 times for all other carbon atoms. Crystallographic data for the structures reported in this paper have been deposited with the Cambridge Crystallographic Data Centre.[16] CCDC 2500424 contain the supplementary crystallographic data for this paper. These data can be obtained free of charge from The Cambridge Crystallographic Data Centre via www.ccdc.cam.ac.uk/structures. This report and the CIF file were generated using FinalCif.[17]

Table 1. Crystal data and structure refinement for Breit_IL_1630_3_a

CCDC number	2500424
Empirical formula	$C_{12}H_{18}O_3$
Formula weight	210.26
Temperature [K]	100(2)
Crystal system	orthorhombic
Space group (number)	$P2_12_12_1$ (19)
a [Å]	6.3216(4)
b [Å]	16.1014(13)
c [Å]	22.707(2)
α [°]	90
β [°]	90
γ [°]	90
Volume [Å^3]	2311.3(3)
Z	8
ρ_{calc} [gcm^{-3}]	1.208
μ [mm^{-1}]	0.693
F(000)	912
Crystal size [mm^3]	0.029×0.048×0.282
Crystal colour	colourless
Crystal shape	needle
Radiation	CuK_α (λ=1.54178 Å)
2θ range [°]	6.73 to 149.22 (0.80 Å)
Index ranges	−7 ≤ h ≤ 7<br>−20 ≤ k ≤ 20<br>−28 ≤ l ≤ 28
Reflections collected	125682
Independent reflections	4727<br>R_{int} = 0.0950<br>R_{sigma} = 0.0232
Completeness to θ = 67.679°	100.0 %
Data / Restraints / Parameters	4727 / 0 / 283
Absorption correction T_{min}/T_{max} (method)	0.6446 / 0.7391 (multi-scan)
Goodness-of-fit on F^2	1.055
Final R indexes [I≥2σ(I)]	R_1 = 0.0323<br>wR_2 = 0.0854
Final R indexes [all data]	R_1 = 0.0340<br>wR_2 = 0.0870
Largest peak/hole [eÅ^{-3}]	0.17/−0.17
Flack X parameter	-0.03(6)

Table 2. Atomic coordinates and U_{eq} [Å^2] for Breit_IL_1630_3_a

**Atom**	***x***	***y***	***z***	U_{eq}
O1	0.5698(3)	0.59983(9)	0.31996(6)	0.0400(4)
O2	0.6343(2)	0.37131(8)	0.52751(5)	0.0242(3)
H2	0.723(4)	0.3397(16)	0.5365(3)	0.036
O3	0.4555(2)	0.23619(7)	0.46150(6)	0.0278(3)
H3A	0.555(5)	0.2172(8)	0.4454(10)	0.042
O4	0.9539(2)	−0.05428(8)	0.19804(6)	0.0328(3)
O5	0.7694(2)	0.15274(8)	0.41026(5)	0.0260(3)
H5	0.880(4)	0.1461(16)	0.4265(6)	0.039
O6	0.8916(3)	0.30036(9)	0.35117(8)	0.0453(4)
H6	0.964(5)	0.3390(16)	0.3346(13)	0.068
C1	0.6179(3)	0.56176(11)	0.36419(8)	0.0261(3)
C2	0.5508(3)	0.47430(10)	0.37914(7)	0.0236(3)
H2A	0.634889	0.433455	0.356480	0.028
H2B	0.399105	0.465952	0.370014	0.028
C3	0.5911(3)	0.46441(9)	0.44560(7)	0.0193(3)
H3	0.465985	0.487623	0.466923	0.023
C4	0.7853(2)	0.52170(10)	0.45915(7)	0.0195(3)
C5	0.7332(3)	0.58490(10)	0.50881(8)	0.0253(3)
H5A	0.629562	0.564291	0.538058	0.030
H5B	0.859398	0.608662	0.528351	0.030
C6	0.6379(3)	0.64046(11)	0.46173(9)	0.0277(4)
C7	0.7581(3)	0.59409(10)	0.41318(7)	0.0236(3)
C8	0.9976(3)	0.47866(11)	0.46198(8)	0.0243(3)
H8A	1.019230	0.446121	0.426002	0.036
H8B	1.001309	0.441664	0.496230	0.036
H8C	1.109967	0.520300	0.465526	0.036
C9	0.4906(4)	0.69858(12)	0.46008(12)	0.0430(5)
H9A	0.417242	0.713528	0.495021	0.052
H9B	0.457987	0.725428	0.423950	0.052
C10	0.9543(3)	0.63922(12)	0.39121(8)	0.0313(4)
H10A	1.045560	0.600076	0.370079	0.047
H10B	1.031526	0.662461	0.424804	0.047
H10C	0.912134	0.684187	0.364598	0.047
C11	0.6165(3)	0.37399(10)	0.46436(7)	0.0208(3)
H11	0.749359	0.351318	0.446641	0.025
C12	0.4302(3)	0.32098(10)	0.44433(7)	0.0234(3)
H12A	0.298163	0.343185	0.461675	0.028
H12B	0.417473	0.324197	0.400933	0.028
C13	0.8818(3)	−0.02201(11)	0.24222(7)	0.0251(3)
C14	0.9032(3)	0.06842(11)	0.25811(7)	0.0249(3)
H14A	0.798416	0.102485	0.236502	0.030
H14B	1.046996	0.089074	0.248986	0.030
C15	0.8609(3)	0.07093(10)	0.32475(7)	0.0220(3)
H15	0.993500	0.053027	0.345122	0.026
C16	0.6901(3)	0.00239(11)	0.33570(7)	0.0236(3)
C17	0.7624(3)	−0.05778(11)	0.38526(8)	0.0266(4)
H17A	0.851977	−0.031425	0.415756	0.032
H17B	0.645915	−0.090103	0.403236	0.032

C18	0.8861(3)	−0.10493(10)	0.33857(8)	0.0251(3)
C19	0.7541(3)	−0.06551(11)	0.28911(8)	0.0248(3)
C20	0.4624(3)	0.03249(12)	0.33632(8)	0.0298(4)
H20A	0.434483	0.064991	0.300666	0.045
H20B	0.438942	0.067309	0.371112	0.045
H20C	0.366844	−0.015403	0.337581	0.045
C21	1.0521(3)	−0.15455(11)	0.33726(9)	0.0301(4)
H21A	1.123955	−0.168047	0.372729	0.036
H21B	1.099682	−0.176796	0.300849	0.036
C22	0.5852(3)	−0.12370(13)	0.26492(8)	0.0331(4)
H22A	0.478478	−0.091334	0.243647	0.050
H22B	0.517809	−0.153492	0.297540	0.050
H22C	0.650754	−0.163724	0.238005	0.050
C23	0.8032(3)	0.15694(11)	0.34798(7)	0.0236(3)
H23	0.667049	0.174020	0.329128	0.028
C24	0.9686(3)	0.22185(11)	0.33292(8)	0.0296(4)
H24A	1.103111	0.209099	0.353354	0.036
H24B	0.995461	0.222131	0.289962	0.036

U_{eq} is defined as 1/3 of the trace of the orthogonalized U_{ij} tensor.

Table 3. Anisotropic displacement parameters [Å^2] for Breit_IL_1630_3_a. The anisotropic displacement factor exponent takes the form: $-2\pi^2[\, h^2(a^*)^2U_{11} + k^2(b^*)^2U_{22} + \ldots + 2hka^*b^*U_{12}\,]$

**Atom**	U_{11}	U_{22}	U_{33}	U_{23}	U_{13}	U_{12}
O1	0.0485(9)	0.0361(7)	0.0355(7)	0.0127(6)	−0.0149(7)	−0.0067(7)
O2	0.0262(6)	0.0246(5)	0.0218(5)	0.0024(4)	−0.0007(5)	0.0023(5)
O3	0.0283(6)	0.0195(5)	0.0355(7)	0.0009(5)	0.0067(5)	0.0003(5)
O4	0.0391(7)	0.0312(6)	0.0280(6)	−0.0055(5)	0.0076(6)	−0.0011(6)
O5	0.0253(6)	0.0321(6)	0.0208(5)	−0.0001(5)	0.0015(5)	0.0012(5)
O6	0.0614(10)	0.0239(6)	0.0505(9)	−0.0025(6)	0.0258(8)	0.0011(7)
C1	0.0261(8)	0.0259(8)	0.0265(8)	0.0024(7)	−0.0043(7)	−0.0008(7)
C2	0.0238(8)	0.0232(8)	0.0236(7)	0.0008(6)	−0.0037(6)	−0.0009(6)
C3	0.0165(7)	0.0194(7)	0.0222(7)	−0.0005(6)	−0.0006(6)	0.0003(5)
C4	0.0170(7)	0.0211(7)	0.0204(7)	0.0002(6)	0.0006(6)	−0.0002(6)
C5	0.0226(8)	0.0267(8)	0.0268(8)	−0.0065(7)	0.0013(7)	−0.0010(6)
C6	0.0223(8)	0.0222(8)	0.0386(9)	−0.0064(7)	−0.0033(7)	−0.0012(6)
C7	0.0232(8)	0.0208(7)	0.0266(8)	0.0022(7)	−0.0027(7)	−0.0013(6)
C8	0.0172(8)	0.0254(7)	0.0303(8)	0.0011(7)	−0.0009(6)	0.0022(6)
C9	0.0332(10)	0.0296(9)	0.0662(14)	−0.0110(10)	−0.0092(10)	0.0066(8)
C10	0.0326(9)	0.0287(9)	0.0328(9)	0.0053(7)	−0.0002(7)	−0.0083(8)
C11	0.0209(7)	0.0204(7)	0.0210(7)	0.0000(6)	0.0016(6)	0.0018(6)
C12	0.0218(8)	0.0196(7)	0.0288(8)	0.0003(6)	0.0000(6)	0.0002(6)
C13	0.0237(8)	0.0286(8)	0.0232(7)	−0.0006(7)	−0.0006(7)	0.0001(7)
C14	0.0263(8)	0.0265(8)	0.0218(7)	0.0000(6)	0.0031(7)	0.0003(7)
C15	0.0195(7)	0.0255(8)	0.0209(7)	0.0003(6)	0.0010(6)	0.0018(6)
C16	0.0196(8)	0.0293(8)	0.0220(7)	−0.0001(7)	−0.0001(6)	0.0013(6)
C17	0.0254(8)	0.0290(8)	0.0253(8)	0.0041(7)	0.0012(7)	0.0003(7)
C18	0.0248(8)	0.0225(7)	0.0281(8)	0.0020(6)	−0.0002(7)	−0.0050(6)

C19	0.0223(8)	0.0273(8)	0.0248(8)	−0.0006(7)	0.0003(7)	−0.0017(7)
C20	0.0197(8)	0.0375(9)	0.0320(8)	0.0016(7)	0.0005(7)	0.0024(7)
C21	0.0282(8)	0.0238(8)	0.0382(9)	−0.0017(7)	−0.0018(8)	−0.0020(7)
C22	0.0300(9)	0.0378(9)	0.0316(9)	−0.0038(7)	−0.0016(8)	−0.0092(8)
C23	0.0243(8)	0.0256(8)	0.0208(7)	0.0009(6)	0.0010(6)	0.0041(7)
C24	0.0349(9)	0.0241(8)	0.0299(8)	−0.0003(7)	0.0076(8)	0.0016(7)

Table 4. Bond lengths and angles for Breit_IL_1630_3_a

**Atom–Atom**	**Length [Å]**
O1–C1	1.215(2)
O2–C11	1.4390(19)
O2–H2	0.78(3)
O3–C12	1.4289(19)
O3–H3A	0.79(3)
O4–C13	1.218(2)
O5–C23	1.4320(19)
O5–H5	0.80(3)
O6–C24	1.417(2)
O6–H6	0.86(3)
C1–C2	1.509(2)
C1–C7	1.514(2)
C2–C3	1.539(2)
C2–H2A	0.9900
C2–H2B	0.9900
C3–C11	1.525(2)
C3–C4	1.566(2)
C3–H3	1.0000
C4–C8	1.512(2)
C4–C5	1.554(2)
C4–C7	1.574(2)
C5–C6	1.518(3)
C5–H5A	0.9900
C5–H5B	0.9900
C6–C9	1.321(3)
C6–C7	1.533(2)
C7–C10	1.522(2)
C8–H8A	0.9800
C8–H8B	0.9800
C8–H8C	0.9800
C9–H9A	0.9500
C9–H9B	0.9500
C10–H10A	0.9800
C10–H10B	0.9800
C10–H10C	0.9800
C11–C12	1.524(2)
C11–H11	1.0000
C12–H12A	0.9900
C12–H12B	0.9900
C13–C14	1.506(2)
C13–C19	1.509(2)
C14–C15	1.537(2)
C14–H14A	0.9900
C14–H14B	0.9900
C15–C23	1.526(2)
C15–C16	1.563(2)
C15–H15	1.0000
C16–C20	1.519(2)
C16–C17	1.554(2)
C16–C19	1.574(2)
C17–C18	1.521(2)
C17–H17A	0.9900
C17–H17B	0.9900
C18–C21	1.319(3)
C18–C19	1.536(2)
C19–C22	1.523(2)
C20–H20A	0.9800
C20–H20B	0.9800
C20–H20C	0.9800
C21–H21A	0.9500
C21–H21B	0.9500
C22–H22A	0.9800
C22–H22B	0.9800
C22–H22C	0.9800
C23–C24	1.518(3)
C23–H23	1.0000
C24–H24A	0.9900
C24–H24B	0.9900
**Atom–Atom–Atom**	**Angle [°]**
C11–O2–H2	109.5
C12–O3–H3A	109.5
C23–O5–H5	109.5
C24–O6–H6	109.5
O1–C1–C2	125.88(17)
O1–C1–C7	125.45(17)
C2–C1–C7	108.67(14)
C1–C2–C3	105.69(13)
C1–C2–H2A	110.6

C3–C2–H2A	110.6
C1–C2–H2B	110.6
C3–C2–H2B	110.6
H2A–C2–H2B	108.7
C11–C3–C2	112.95(13)
C11–C3–C4	115.15(13)
C2–C3–C4	105.17(12)
C11–C3–H3	107.7
C2–C3–H3	107.7
C4–C3–H3	107.7
C8–C4–C5	117.22(14)
C8–C4–C3	115.74(13)
C5–C4–C3	111.23(13)
C8–C4–C7	117.67(14)
C5–C4–C7	88.46(12)
C3–C4–C7	102.74(12)
C6–C5–C4	87.65(12)
C6–C5–H5A	114.0
C4–C5–H5A	114.0
C6–C5–H5B	114.0
C4–C5–H5B	114.0
H5A–C5–H5B	111.2
C9–C6–C5	135.8(2)
C9–C6–C7	132.4(2)
C5–C6–C7	91.31(13)
C1–C7–C10	113.60(15)
C1–C7–C6	113.93(15)
C10–C7–C6	114.02(15)
C1–C7–C4	107.24(13)
C10–C7–C4	118.82(14)
C6–C7–C4	86.43(12)
C4–C8–H8A	109.5
C4–C8–H8B	109.5
H8A–C8–H8B	109.5
C4–C8–H8C	109.5
H8A–C8–H8C	109.5
H8B–C8–H8C	109.5
C6–C9–H9A	120.0
C6–C9–H9B	120.0
H9A–C9–H9B	120.0
C7–C10–H10A	109.5
C7–C10–H10B	109.5
H10A–C10–H10B	109.5
C7–C10–H10C	109.5
H10A–C10–H10C	109.5
H10B–C10–H10C	109.5
O2–C11–C12	109.93(13)
O2–C11–C3	108.36(13)
C12–C11–C3	111.71(13)
O2–C11–H11	108.9

C12–C11–H11	108.9
C3–C11–H11	108.9
O3–C12–C11	111.54(14)
O3–C12–H12A	109.3
C11–C12–H12A	109.3
O3–C12–H12B	109.3
C11–C12–H12B	109.3
H12A–C12–H12B	108.0
O4–C13–C14	125.18(16)
O4–C13–C19	125.69(16)
C14–C13–C19	109.12(14)
C13–C14–C15	104.22(14)
C13–C14–H14A	110.9
C15–C14–H14A	110.9
C13–C14–H14B	110.9
C15–C14–H14B	110.9
H14A–C14–H14B	108.9
C23–C15–C14	113.92(14)
C23–C15–C16	114.88(13)
C14–C15–C16	104.96(13)
C23–C15–H15	107.6
C14–C15–H15	107.6
C16–C15–H15	107.6
C20–C16–C17	118.10(15)
C20–C16–C15	115.50(14)
C17–C16–C15	110.62(14)
C20–C16–C19	118.11(15)
C17–C16–C19	88.74(13)
C15–C16–C19	101.89(13)
C18–C17–C16	87.57(13)
C18–C17–H17A	114.1
C16–C17–H17A	114.1
C18–C17–H17B	114.1
C16–C17–H17B	114.1
H17A–C17–H17B	111.2
C21–C18–C17	136.65(18)
C21–C18–C19	131.75(17)
C17–C18–C19	91.37(13)
C13–C19–C22	113.96(15)
C13–C19–C18	114.63(15)
C22–C19–C18	112.98(15)
C13–C19–C16	106.82(14)
C22–C19–C16	119.32(15)
C18–C19–C16	86.29(12)
C16–C20–H20A	109.5
C16–C20–H20B	109.5
H20A–C20–H20B	109.5
C16–C20–H20C	109.5
H20A–C20–H20C	109.5
H20B–C20–H20C	109.5

C18–C21–H21A	120.0
C18–C21–H21B	120.0
H21A–C21–H21B	120.0
C19–C22–H22A	109.5
C19–C22–H22B	109.5
H22A–C22–H22B	109.5
C19–C22–H22C	109.5
H22A–C22–H22C	109.5
H22B–C22–H22C	109.5
O5–C23–C24	110.97(14)
O5–C23–C15	109.52(13)
C24–C23–C15	112.50(14)
O5–C23–H23	107.9
C24–C23–H23	107.9
C15–C23–H23	107.9
O6–C24–C23	108.16(15)
O6–C24–H24A	110.1
C23–C24–H24A	110.1
O6–C24–H24B	110.1
C23–C24–H24B	110.1
H24A–C24–H24B	108.4

Table 5. Torsion angles for Breit_IL_1630_3_a

**Atom–Atom–Atom–Atom**	**Torsion Angle [°]**
O1–C1–C2–C3	−162.94(19)
C7–C1–C2–C3	17.82(18)
C1–C2–C3–C11	−157.36(14)
C1–C2–C3–C4	−30.99(17)
C11–C3–C4–C8	26.89(19)
C2–C3–C4–C8	−98.11(15)
C11–C3–C4–C5	−110.16(15)
C2–C3–C4–C5	124.84(14)
C11–C3–C4–C7	156.50(13)
C2–C3–C4–C7	31.49(15)
C8–C4–C5–C6	139.01(15)
C3–C4–C5–C6	−84.63(14)
C7–C4–C5–C6	18.44(12)
C4–C5–C6–C9	153.3(2)
C4–C5–C6–C7	−18.95(12)
O1–C1–C7–C10	−43.6(3)
C2–C1–C7–C10	135.60(16)
O1–C1–C7–C6	89.2(2)
C2–C1–C7–C6	−91.55(17)
O1–C1–C7–C4	−176.99(19)
C2–C1–C7–C4	2.25(18)
C9–C6–C7–C1	−46.7(3)
C5–C6–C7–C1	126.01(15)
C9–C6–C7–C10	86.0(3)
C5–C6–C7–C10	−101.34(16)
C9–C6–C7–C4	−153.9(2)
C5–C6–C7–C4	18.72(12)
C8–C4–C7–C1	107.59(16)
C5–C4–C7–C1	−132.25(14)
C3–C4–C7–C1	−20.82(16)
C8–C4–C7–C10	−22.9(2)
C5–C4–C7–C10	97.27(16)
C3–C4–C7–C10	−151.30(15)
C8–C4–C7–C6	−138.45(15)
C5–C4–C7–C6	−18.28(12)
C3–C4–C7–C6	93.15(13)
C2–C3–C11–O2	−174.89(13)
C4–C3–C11–O2	64.26(17)
C2–C3–C11–C12	−53.64(18)
C4–C3–C11–C12	−174.49(13)
O2–C11–C12–O3	−60.20(17)
C3–C11–C12–O3	179.47(13)
O4–C13–C14–C15	−160.17(18)
C19–C13–C14–C15	20.80(18)
C13–C14–C15–C23	−161.26(14)
C13–C14–C15–C16	−34.75(17)
C23–C15–C16–C20	31.2(2)
C14–C15–C16–C20	−94.69(17)
C23–C15–C16–C17	−106.26(16)
C14–C15–C16–C17	127.83(14)
C23–C15–C16–C19	160.58(14)
C14–C15–C16–C19	34.66(16)
C20–C16–C17–C18	139.80(16)
C15–C16–C17–C18	−83.95(15)
C19–C16–C17–C18	18.29(12)
C16–C17–C18–C21	155.9(2)
C16–C17–C18–C19	−18.76(13)
O4–C13–C19–C22	−44.0(3)
C14–C13–C19–C22	135.04(16)
O4–C13–C19–C18	88.4(2)
C14–C13–C19–C18	−92.60(18)
O4–C13–C19–C16	−177.94(17)
C14–C13–C19–C16	1.08(18)
C21–C18–C19–C13	−49.8(3)
C17–C18–C19–C13	125.34(15)
C21–C18–C19–C22	83.0(2)
C17–C18–C19–C22	−101.84(16)
C21–C18–C19–C16	−156.6(2)

C17–C18–C19–C16	18.53(13)
C20–C16–C19–C13	105.76(17)
C17–C16–C19–C13	−132.74(14)
C15–C16–C19–C13	−21.93(16)
C20–C16–C19–C22	−25.3(2)
C17–C16–C19–C22	96.23(17)
C15–C16–C19–C22	−152.95(16)
C20–C16–C19–C18	−139.62(16)
C17–C16–C19–C18	−18.12(12)
C15–C16–C19–C18	92.70(13)
C14–C15–C23–O5	−179.36(14)
C16–C15–C23–O5	59.50(18)
C14–C15–C23–C24	−55.46(19)
C16–C15–C23–C24	−176.59(14)
O5–C23–C24–O6	−62.5(2)
C15–C23–C24–O6	174.41(15)

Compound **2**

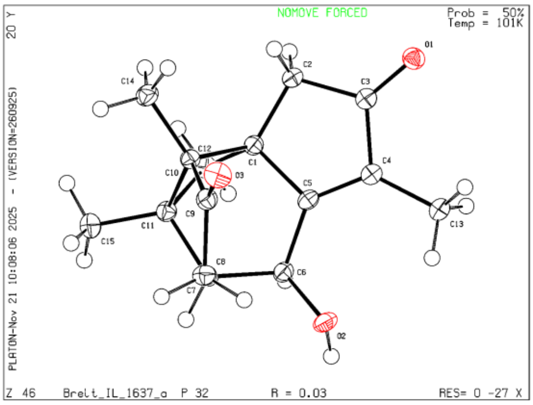

Crystals were obtained at room temperature by slow solvent evaporation from asolution of the compound dissolved in a mixture of diethylether and dichloromethane. A colourless, block-shaped crystal was mounted on a MiTeGen micromount with perfluoroether oil. Data for Breit_IL_1637_a were collected from a shock-cooled single crystal at 101(2) K on a Bruker D8 VENTURE dual wavelength Mo/Cu three-circle diffractometer with a microfocus sealed X-ray tube using a mirror optics as monochromator and a Bruker PHOTON III detector. The diffractometer was equipped with an Oxford Cryostream 800 low temperature device and used CuK_α radiation (λ = 1.54178 Å). All data were integrated with SAINT V8.42 and a multi-scan absorption correction using SADABS 2016/2 was applied.[12,13] The structure was solved by direct methods with SHELXT and refined by full-matrix least-squares methods against F^2 using SHELXL-2019/2.[14,15] All non-hydrogen atoms were refined with anisotropic displacement parameters. All C-bound hydrogen atoms were refined isotropic on calculated positions using a riding model with their U_{iso} values constrained to 1.5 times the U_{eq} of their pivot atoms for terminal sp^3 carbon atoms and 1.2 times for all other carbon atoms. Crystallographic data for the structures reported in this paper have been deposited with the Cambridge Crystallographic Data Centre.[16] CCDC 2504773 contain the supplementary crystallographic data for this paper. These data can be obtained free of charge from The Cambridge Crystallographic Data Centre via www.ccdc.cam.ac.uk/ structures. This report and the CIF file were generated using FinalCif.[17]

Table 6. Crystal data and structure refinement for Breit_IL_1637_a

CCDC number	2504773
Empirical formula	$C_{15}H_{18}O_3$
Formula weight	246.29
Temperature [K]	101(2)
Crystal system	trigonal
Space group (number)	$P3_2$ (145)
a [Å]	7.6005(3)
b [Å]	7.6005(3)
c [Å]	18.8568(11)
α [°]	90
β [°]	90
γ [°]	120
Volume [Å^3]	943.37(9)
Z	3
ρ_{calc} [gcm^{-3}]	1.301
μ [mm^{-1}]	0.723
F(000)	396
Crystal size [mm^3]	0.161×0.221×0.268
Crystal colour	colourless
Crystal shape	block
Radiation	CuK_α (λ=1.54178 Å)
2θ range [°]	13.45 to 149.12 (0.80 Å)
Index ranges	−9 ≤ h ≤ 9<br>−9 ≤ k ≤ 9<br>−23 ≤ l ≤ 23
Reflections collected	34843
Independent reflections	2600<br>R_{int} = 0.0547<br>R_{sigma} = 0.0226
Completeness to θ = 67.679°	100.0 %
Data / Restraints / Parameters	2600 / 1 / 168
Absorption correction T_{min}/T_{max} (method)	0.6712 / 0.7538 (multi-scan)
Goodness-of-fit on F^2	1.096
Final *R* indexes [$I \geq 2\sigma(I)$]	R_1 = 0.0321<br>wR_2 = 0.0849
Final *R* indexes [all data]	R_1 = 0.0321<br>wR_2 = 0.0849
Largest peak/hole [eÅ^{-3}]	0.22/−0.20
Flack X parameter	0.01(6)

Table 7. Atomic coordinates and U_{eq} [Å^2] for Breit_IL_1637_a

Atom	*x*	*y*	*z*	U_{eq}
O1	0.7875(2)	0.9877(2)	0.37858(7)	0.0192(3)
O2	0.7383(2)	0.2767(2)	0.45298(8)	0.0206(3)
H2	0.734(2)	0.178(5)	0.4297(18)	0.031
O3	0.5974(2)	0.6099(2)	0.62208(8)	0.0240(3)
C1	0.4072(3)	0.5125(3)	0.45195(10)	0.0154(4)
C2	0.4662(3)	0.7255(3)	0.42745(11)	0.0184(4)
H2A	0.375157	0.720943	0.389032	0.022
H2B	0.459701	0.807125	0.467232	0.022
C3	0.6822(3)	0.8137(3)	0.40080(10)	0.0158(4)
C4	0.7428(3)	0.6576(3)	0.40455(9)	0.0162(4)
C5	0.5872(3)	0.4890(3)	0.43292(10)	0.0154(4)
C6	0.5532(3)	0.2773(3)	0.44295(10)	0.0167(4)
H6	0.485701	0.196740	0.399348	0.020
C7	0.4066(3)	0.1790(3)	0.50604(11)	0.0171(4)
H7	0.353013	0.029120	0.507096	0.020
C8	0.5173(3)	0.2779(3)	0.57575(10)	0.0196(4)
H8A	0.451114	0.185897	0.616474	0.024
H8B	0.661350	0.312726	0.573590	0.024
C9	0.5006(3)	0.4683(3)	0.58208(10)	0.0179(4)
C10	0.3362(3)	0.4470(3)	0.53188(10)	0.0155(4)
C11	0.2273(3)	0.2254(3)	0.50255(10)	0.0162(4)
C12	0.2121(3)	0.3139(3)	0.42985(10)	0.0175(4)
H12A	0.229948	0.245011	0.388225	0.021
H12B	0.090299	0.328417	0.424693	0.021
C13	0.9467(3)	0.7003(3)	0.37808(11)	0.0208(4)
H13A	0.945106	0.694927	0.326147	0.031
H13B	0.976994	0.598276	0.396992	0.031
H13C	1.051194	0.835733	0.393822	0.031
C14	0.2076(3)	0.5331(3)	0.56032(11)	0.0204(4)
H14A	0.120671	0.535274	0.522343	0.031
H14B	0.296497	0.671633	0.577610	0.031
H14C	0.122597	0.447779	0.599355	0.031
C15	0.0355(3)	0.0632(3)	0.53843(11)	0.0207(4)
H15A	−0.069834	0.101505	0.535987	0.031
H15B	0.064775	0.050586	0.588180	0.031
H15C	−0.012242	−0.067039	0.514269	0.031

U_{eq} is defined as 1/3 of the trace of the orthogonalized U_{ij} tensor.

Table 8. Anisotropic displacement parameters [Å^2] for Breit_IL_1637_a. The anisotropic displacement factor exponent takes the form: $-2\pi^2[\, h^2(a^*)^2U_{11} + k^2(b^*)^2U_{22} + \ldots + 2hka^*b^*U_{12}\,]$

Atom	U_{11}	U_{22}	U_{33}	U_{23}	U_{13}	U_{12}
O1	0.0201(7)	0.0170(7)	0.0198(7)	−0.0004(5)	0.0002(5)	0.0088(5)
O2	0.0175(7)	0.0201(7)	0.0282(7)	−0.0033(6)	−0.0006(6)	0.0124(6)
O3	0.0266(7)	0.0231(7)	0.0207(7)	−0.0045(6)	−0.0056(6)	0.0113(6)
C1	0.0153(8)	0.0170(8)	0.0151(8)	0.0009(7)	0.0007(7)	0.0088(7)
C2	0.0174(9)	0.0198(9)	0.0210(9)	0.0022(7)	0.0024(7)	0.0117(8)
C3	0.0170(8)	0.0178(9)	0.0129(8)	−0.0021(7)	−0.0016(7)	0.0090(7)

C4	0.0159(9)	0.0177(8)	0.0157(8)	−0.0022(7)	−0.0016(7)	0.0089(7)
C5	0.0154(8)	0.0182(9)	0.0143(8)	−0.0030(6)	−0.0016(7)	0.0098(7)
C6	0.0143(8)	0.0161(8)	0.0198(9)	−0.0017(7)	0.0002(7)	0.0078(7)
C7	0.0176(8)	0.0154(8)	0.0194(9)	0.0000(7)	0.0010(7)	0.0091(7)
C8	0.0234(10)	0.0206(9)	0.0186(9)	0.0002(7)	−0.0007(7)	0.0138(8)
C9	0.0189(9)	0.0197(9)	0.0159(8)	0.0011(7)	0.0007(7)	0.0103(7)
C10	0.0167(8)	0.0152(8)	0.0155(8)	0.0001(7)	0.0011(7)	0.0086(7)
C11	0.0159(9)	0.0158(9)	0.0165(9)	−0.0011(7)	0.0002(7)	0.0077(7)
C12	0.0160(8)	0.0193(9)	0.0161(9)	−0.0004(7)	−0.0005(7)	0.0079(7)
C13	0.0175(9)	0.0218(9)	0.0249(10)	0.0020(8)	0.0038(7)	0.0111(7)
C14	0.0237(9)	0.0240(10)	0.0180(9)	0.0009(7)	0.0035(7)	0.0153(8)
C15	0.0183(9)	0.0203(9)	0.0204(10)	0.0004(7)	0.0012(7)	0.0072(8)

Table 9. Bond lengths and angles for Breit_IL_1637_a

**Atom–Atom**	**Length [Å]**
O1–C3	1.227(3)
O2–C6	1.422(2)
O2–H2	0.86(4)
O3–C9	1.215(3)
C1–C5	1.509(2)
C1–C2	1.520(3)
C1–C12	1.553(3)
C1–C10	1.594(3)
C2–C3	1.516(3)
C2–H2A	0.9900
C2–H2B	0.9900
C3–C4	1.474(3)
C4–C5	1.346(3)
C4–C13	1.501(3)
C5–C6	1.508(3)
C6–C7	1.543(3)
C6–H6	1.0000
C7–C8	1.539(3)
C7–C11	1.571(2)
C7–H7	1.0000
C8–C9	1.520(3)
C8–H8A	0.9900
C8–H8B	0.9900
C9–C10	1.510(3)
C10–C14	1.520(3)
C10–C11	1.560(3)
C11–C15	1.518(3)
C11–C12	1.557(3)
C12–H12A	0.9900
C12–H12B	0.9900
C13–H13A	0.9800
C13–H13B	0.9800
C13–H13C	0.9800

C14–H14A	0.9800
C14–H14B	0.9800
C14–H14C	0.9800
C15–H15A	0.9800
C15–H15B	0.9800
C15–H15C	0.9800
**Atom–Atom–Atom**	**Angle [°]**
C6–O2–H2	109.5
C5–C1–C2	104.62(15)
C5–C1–C12	107.62(15)
C2–C1–C12	125.66(16)
C5–C1–C10	111.93(15)
C2–C1–C10	119.56(15)
C12–C1–C10	86.59(14)
C3–C2–C1	104.41(15)
C3–C2–H2A	110.9
C1–C2–H2A	110.9
C3–C2–H2B	110.9
C1–C2–H2B	110.9
H2A–C2–H2B	108.9
O1–C3–C4	125.86(17)
O1–C3–C2	124.62(18)
C4–C3–C2	109.50(16)
C5–C4–C3	107.82(16)
C5–C4–C13	131.21(17)
C3–C4–C13	120.96(17)
C4–C5–C6	131.76(17)
C4–C5–C1	113.49(16)
C6–C5–C1	114.49(16)
O2–C6–C5	112.21(15)
O2–C6–C7	111.80(16)
C5–C6–C7	108.10(15)
O2–C6–H6	108.2

C5–C6–H6	108.2
C7–C6–H6	108.2
C8–C7–C6	109.63(16)
C8–C7–C11	104.48(15)
C6–C7–C11	110.86(15)
C8–C7–H7	110.6
C6–C7–H7	110.6
C11–C7–H7	110.6
C9–C8–C7	104.27(16)
C9–C8–H8A	110.9
C7–C8–H8A	110.9
C9–C8–H8B	110.9
C7–C8–H8B	110.9
H8A–C8–H8B	108.9
O3–C9–C10	125.85(17)
O3–C9–C8	126.19(18)
C10–C9–C8	107.92(16)
C9–C10–C14	114.10(16)
C9–C10–C11	107.55(15)
C14–C10–C11	118.78(16)
C9–C10–C1	115.22(15)
C14–C10–C1	112.94(15)
C11–C10–C1	85.19(14)
C15–C11–C12	119.66(16)
C15–C11–C10	119.63(16)
C12–C11–C10	87.67(14)
C15–C11–C7	112.82(16)
C12–C11–C7	113.21(16)
C10–C11–C7	100.14(14)
C1–C12–C11	86.71(14)
C1–C12–H12A	114.2
C11–C12–H12A	114.2
C1–C12–H12B	114.2
C11–C12–H12B	114.2
H12A–C12–H12B	111.4
C4–C13–H13A	109.5
C4–C13–H13B	109.5
H13A–C13–H13B	109.5
C4–C13–H13C	109.5
H13A–C13–H13C	109.5
H13B–C13–H13C	109.5
C10–C14–H14A	109.5
C10–C14–H14B	109.5
H14A–C14–H14B	109.5
C10–C14–H14C	109.5
H14A–C14–H14C	109.5
H14B–C14–H14C	109.5
C11–C15–H15A	109.5
C11–C15–H15B	109.5
H15A–C15–H15B	109.5
C11–C15–H15C	109.5
H15A–C15–H15C	109.5
H15B–C15–H15C	109.5

Table 10. Torsion angles for Breit_IL_1637_a

**Atom–Atom–Atom–Atom**	**Torsion Angle [°]**
C5–C1–C2–C3	−3.85(19)
C12–C1–C2–C3	−128.75(18)
C10–C1–C2–C3	122.42(18)
C1–C2–C3–O1	−177.34(17)
C1–C2–C3–C4	3.8(2)
O1–C3–C4–C5	179.04(18)
C2–C3–C4–C5	−2.1(2)
O1–C3–C4–C13	−1.9(3)
C2–C3–C4–C13	177.01(17)
C3–C4–C5–C6	173.10(18)
C13–C4–C5–C6	−5.9(4)
C3–C4–C5–C1	−0.6(2)
C13–C4–C5–C1	−179.53(18)
C2–C1–C5–C4	2.9(2)
C12–C1–C5–C4	138.57(17)
C10–C1–C5–C4	−127.95(17)
C2–C1–C5–C6	−171.89(16)
C12–C1–C5–C6	−36.3(2)
C10–C1–C5–C6	57.2(2)
C4–C5–C6–O2	30.4(3)
C1–C5–C6–O2	−155.93(16)
C4–C5–C6–C7	154.2(2)
C1–C5–C6–C7	−32.2(2)
O2–C6–C7–C8	52.1(2)
C5–C6–C7–C8	−71.86(18)
O2–C6–C7–C11	166.96(15)
C5–C6–C7–C11	43.0(2)
C6–C7–C8–C9	83.73(18)
C11–C7–C8–C9	−35.1(2)
C7–C8–C9–O3	−165.8(2)
C7–C8–C9–C10	16.4(2)
O3–C9–C10–C14	−35.2(3)
C8–C9–C10–C14	142.61(17)
O3–C9–C10–C11	−169.20(19)
C8–C9–C10–C11	8.6(2)
O3–C9–C10–C1	97.8(2)

C8–C9–C10–C1	−84.35(18)
C5–C1–C10–C9	27.0(2)
C2–C1–C10–C9	−95.8(2)
C12–C1–C10–C9	134.62(16)
C5–C1–C10–C14	160.57(16)
C2–C1–C10–C14	37.8(2)
C12–C1–C10–C14	−91.80(17)
C5–C1–C10–C11	−80.15(16)
C2–C1–C10–C11	157.09(17)
C12–C1–C10–C11	27.48(13)
C9–C10–C11–C15	94.5(2)
C14–C10–C11–C15	−36.9(3)
C1–C10–C11–C15	−150.51(17)
C9–C10–C11–C12	−142.33(15)
C14–C10–C11–C12	86.19(18)
C1–C10–C11–C12	−27.38(13)
C9–C10–C11–C7	−29.16(18)

C14–C10–C11–C7	−160.64(17)
C1–C10–C11–C7	85.78(14)
C8–C7–C11–C15	−89.20(19)
C6–C7–C11–C15	152.78(17)
C8–C7–C11–C12	130.94(17)
C6–C7–C11–C12	12.9(2)
C8–C7–C11–C10	39.12(18)
C6–C7–C11–C10	−78.90(18)
C5–C1–C12–C11	84.45(15)
C2–C1–C12–C11	−151.92(18)
C10–C1–C12–C11	−27.49(13)
C15–C11–C12–C1	151.22(17)
C10–C11–C12–C1	28.11(13)
C7–C11–C12–C1	−71.92(17)

Compound **28**

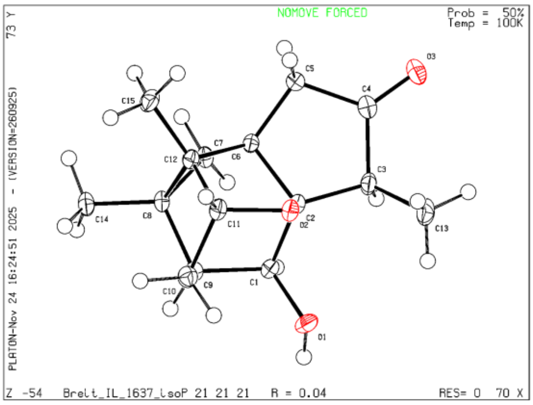

Crystals were obtained from diethylether by solvent evaporation at the rotavap. A colourless, needle-shaped crystal was mounted on a MiTeGen micromount with perfluoroether oil. Data for Breit_IL_1637_iso_a were collected from a shock-cooled single crystal at 100(2) K on a Bruker D8 VENTURE dual wavelength Mo/Cu three-circle diffractometer with a microfocus sealed X-ray tube using a mirror optics as monochromator and a Bruker PHOTON III detector. The diffractometer was equipped with an Oxford Cryostream 800 low temperature device and used CuK_{α} radiation (λ = 1.54178 Å). All data were integrated with SAINT V8.42 and a multi-scan absorption correction using SADABS 2016/2 was applied.[12,13] The structure was solved by direct methods with SHELXT and refined by full-matrix least-squares methods against F^2 using SHELXL-2019/2.[14,15] All non-hydrogen atoms were refined with anisotropic displacement parameters. All C-bound hydrogen atoms were refined isotropic on calculated positions using a riding model with their U_{iso} values constrained to 1.5 times the U_{eq} of their pivot atoms for terminal sp^3 carbon atoms and 1.2 times for all other carbon atoms. Crystallographic data for the structures reported in this paper have been deposited with the Cambridge Crystallographic Data Centre.[16] CCDC 2505248 contain the supplementary crystallographic data for this paper. These data can be obtained free of charge from The Cambridge Crystallographic Data Centre via www.ccdc.cam.ac.uk/structures. This report and the CIF file were generated using FinalCif.[17]

Table 11. Crystal data and structure refinement for Breit_IL_1637_iso_a

CCDC number	2505248
Empirical formula	$C_{15}H_{20}O_3$
Formula weight	248.31
Temperature [K]	100(2)
Crystal system	orthorhombic
Space group (number)	$P2_12_12_1$ (19)
a [Å]	6.0655(5)
b [Å]	10.8245(11)
c [Å]	19.3397(16)
α [°]	90
β [°]	90
γ [°]	90
Volume [Å^3]	1269.8(2)
Z	4
ρ_{calc} [gcm^{-3}]	1.299
μ [mm^{-1}]	0.716
F(000)	536
Crystal size [mm^3]	0.075×0.118×0.395
Crystal colour	colourless
Crystal shape	needle
Radiation	CuK_{α} (λ=1.54178 Å)
2θ range [°]	9.36 to 155.12 (0.79 Å)
Index ranges	−7 ≤ h ≤ 7<br>−11 ≤ k ≤ 12<br>−24 ≤ l ≤ 24
Reflections collected	59034
Independent reflections	2666<br>R_{int} = 0.0634<br>R_{sigma} = 0.0205
Completeness to θ = 67.679°	99.5 %
Data / Restraints / Parameters	2666 / 0 / 168
Absorption correction T_{min}/T_{max} (method)	0.6085 / 0.7541 (multi-scan)
Goodness-of-fit on F^2	1.084
Final R indexes [I≥2σ(I)]	R_1 = 0.0357<br>wR_2 = 0.0938
Final R indexes [all data]	R_1 = 0.0359<br>wR_2 = 0.0941
Largest peak/hole [eÅ^{-3}]	0.31/−0.30
Flack X parameter	-0.02(5)

Table 12. Atomic coordinates and U_{eq} [Å^2] for Breit_IL_1637_iso_a

Atom	*x*	*y*	*z*	U_{eq}
O1	0.5605(2)	0.27698(12)	0.50940(7)	0.0216(3)
H1A	0.453(4)	0.231(2)	0.4998(11)	0.032
O2	0.8774(2)	0.42558(11)	0.56540(6)	0.0158(3)
O3	0.7462(2)	0.76714(12)	0.47070(7)	0.0217(3)
C1	0.4939(3)	0.36852(15)	0.55806(9)	0.0162(3)
H1	0.341999	0.398404	0.546974	0.019
C2	0.6586(3)	0.47519(15)	0.55312(8)	0.0145(3)
C3	0.6551(3)	0.55098(17)	0.48675(8)	0.0172(4)
H3	0.499270	0.552460	0.469920	0.021
C4	0.7125(3)	0.68178(16)	0.50976(9)	0.0162(3)
C5	0.7198(3)	0.68938(16)	0.58844(8)	0.0160(3)
H5A	0.872992	0.701214	0.604783	0.019
H5B	0.628476	0.758999	0.605243	0.019
C6	0.6281(3)	0.56688(15)	0.61396(8)	0.0134(3)
C7	0.4009(3)	0.55305(16)	0.64887(8)	0.0153(3)
H7A	0.278124	0.537796	0.616226	0.018
H7B	0.364428	0.620189	0.681698	0.018
C8	0.4932(3)	0.43433(16)	0.68371(8)	0.0148(3)
C9	0.5039(3)	0.32177(16)	0.63374(8)	0.0162(3)
H9	0.387396	0.259009	0.643914	0.019
C10	0.7381(3)	0.27135(16)	0.64631(9)	0.0184(4)
H10A	0.753713	0.236184	0.693313	0.022
H10B	0.779111	0.208182	0.611603	0.022
C11	0.8734(3)	0.38945(16)	0.63777(9)	0.0158(3)
H11	1.024367	0.382455	0.658215	0.019
C12	0.7315(3)	0.48966(16)	0.67350(8)	0.0144(3)
C13	0.7982(4)	0.50711(19)	0.42686(9)	0.0286(5)
H13A	0.744102	0.427121	0.410277	0.043
H13B	0.951069	0.498390	0.442567	0.043
H13C	0.791633	0.567591	0.389212	0.043
C14	0.4144(3)	0.40161(17)	0.75604(8)	0.0187(4)
H14A	0.260816	0.373657	0.753982	0.028
H14B	0.424642	0.474602	0.785843	0.028
H14C	0.506799	0.335469	0.774928	0.028
C15	0.8351(3)	0.55864(18)	0.73326(9)	0.0192(4)
H15A	0.966189	0.602877	0.717018	0.029
H15B	0.877436	0.499816	0.769412	0.029
H15C	0.728688	0.617923	0.752062	0.029

U_{eq} is defined as 1/3 of the trace of the orthogonalized U_{ij} tensor.

Table 13. Anisotropic displacement parameters [Å^2] for Breit_IL_1637_iso_a. The anisotropic displacement factor exponent takes the form: $-2\pi^2[\, h^2(a^*)^2U_{11} + k^2(b^*)^2U_{22} + \ldots + 2hka^*b^*U_{12}\,]$

Atom	U_{11}	U_{22}	U_{33}	U_{23}	U_{13}	U_{12}
O1	0.0268(7)	0.0199(7)	0.0182(6)	−0.0068(5)	0.0001(5)	−0.0042(5)
O2	0.0166(6)	0.0190(6)	0.0119(5)	0.0003(5)	0.0013(4)	0.0015(5)
O3	0.0210(6)	0.0244(7)	0.0197(6)	0.0075(5)	0.0005(5)	−0.0034(5)
C1	0.0193(8)	0.0170(8)	0.0123(7)	−0.0019(6)	−0.0002(6)	−0.0014(6)

C2	0.0160(7)	0.0165(8)	0.0109(7)	−0.0001(6)	0.0004(6)	0.0003(6)
C3	0.0205(8)	0.0209(8)	0.0101(7)	0.0015(6)	0.0003(6)	−0.0005(7)
C4	0.0123(7)	0.0198(8)	0.0164(8)	0.0032(6)	0.0007(6)	0.0014(6)
C5	0.0186(8)	0.0148(8)	0.0145(7)	−0.0001(6)	0.0007(6)	−0.0020(6)
C6	0.0156(7)	0.0142(8)	0.0105(7)	0.0001(6)	0.0010(6)	0.0003(6)
C7	0.0159(7)	0.0175(8)	0.0124(7)	0.0019(6)	0.0026(6)	0.0003(6)
C8	0.0163(7)	0.0177(8)	0.0104(7)	0.0022(6)	0.0012(6)	−0.0003(6)
C9	0.0184(8)	0.0162(8)	0.0139(7)	0.0007(6)	0.0003(6)	−0.0016(7)
C10	0.0211(8)	0.0181(8)	0.0159(8)	0.0013(6)	−0.0010(7)	0.0012(7)
C11	0.0168(8)	0.0187(8)	0.0119(7)	0.0006(6)	−0.0006(6)	0.0019(7)
C12	0.0165(8)	0.0159(8)	0.0108(7)	0.0006(6)	−0.0015(6)	−0.0006(6)
C13	0.0449(12)	0.0259(9)	0.0149(8)	0.0013(7)	0.0098(8)	0.0050(9)
C14	0.0208(8)	0.0227(9)	0.0125(7)	0.0037(6)	0.0023(6)	−0.0015(7)
C15	0.0201(8)	0.0244(9)	0.0131(7)	−0.0026(7)	−0.0020(6)	−0.0020(7)

Table 14. Bond lengths and angles for Breit_IL_1637_iso_a

**Atom–Atom**	**Length [Å]**
O1–C1	1.425(2)
O1–H1A	0.84(3)
O2–C2	1.4512(19)
O2–C11	1.4534(19)
O3–C4	1.211(2)
C1–C2	1.530(2)
C1–C9	1.550(2)
C1–H1	1.0000
C2–C3	1.523(2)
C2–C6	1.550(2)
C3–C13	1.523(2)
C3–C4	1.524(3)
C3–H3	1.0000
C4–C5	1.524(2)
C5–C6	1.520(2)
C5–H5A	0.9900
C5–H5B	0.9900
C6–C7	1.542(2)
C6–C12	1.555(2)
C7–C8	1.555(2)
C7–H7A	0.9900
C7–H7B	0.9900
C8–C14	1.520(2)
C8–C9	1.556(2)
C8–C12	1.577(2)
C9–C10	1.541(2)
C9–H9	1.0000
C10–C11	1.528(2)
C10–H10A	0.9900
C10–H10B	0.9900
C11–C12	1.548(2)
C11–H11	1.0000
C12–C15	1.513(2)
C13–H13A	0.9800
C13–H13B	0.9800
C13–H13C	0.9800
C14–H14A	0.9800
C14–H14B	0.9800
C14–H14C	0.9800
C15–H15A	0.9800
C15–H15B	0.9800
C15–H15C	0.9800
**Atom–Atom–Atom**	**Angle [°]**
C1–O1–H1A	109.5
C2–O2–C11	104.00(12)
O1–C1–C2	107.37(14)
O1–C1–C9	112.68(14)
C2–C1–C9	106.25(14)
O1–C1–H1	110.1
C2–C1–H1	110.1
C9–C1–H1	110.1
O2–C2–C3	110.49(13)
O2–C2–C1	107.91(13)
C3–C2–C1	116.74(14)
O2–C2–C6	102.84(12)
C3–C2–C6	107.03(13)
C1–C2–C6	110.95(13)
C13–C3–C2	117.69(15)
C13–C3–C4	112.41(16)
C2–C3–C4	104.54(13)

C13–C3–H3	107.2
C2–C3–H3	107.2
C4–C3–H3	107.2
O3–C4–C3	124.41(16)
O3–C4–C5	125.22(17)
C3–C4–C5	110.37(14)
C6–C5–C4	105.45(14)
C6–C5–H5A	110.7
C4–C5–H5A	110.7
C6–C5–H5B	110.7
C4–C5–H5B	110.7
H5A–C5–H5B	108.8
C5–C6–C7	123.63(14)
C5–C6–C2	105.56(13)
C7–C6–C2	112.15(13)
C5–C6–C12	124.16(14)
C7–C6–C12	89.09(12)
C2–C6–C12	99.76(12)
C6–C7–C8	87.04(12)
C6–C7–H7A	114.1
C8–C7–H7A	114.1
C6–C7–H7B	114.1
C8–C7–H7B	114.1
H7A–C7–H7B	111.3
C14–C8–C7	118.56(14)
C14–C8–C9	113.71(15)
C7–C8–C9	113.14(13)
C14–C8–C12	119.46(14)
C7–C8–C12	87.82(12)
C9–C8–C12	100.43(13)
C10–C9–C1	107.51(14)
C10–C9–C8	102.60(13)
C1–C9–C8	109.21(13)
C10–C9–H9	112.3
C1–C9–H9	112.3
C8–C9–H9	112.3
C11–C10–C9	100.48(14)
C11–C10–H10A	111.7
C9–C10–H10A	111.7
C11–C10–H10B	111.7
C9–C10–H10B	111.7
H10A–C10–H10B	109.4
O2–C11–C10	109.76(13)
O2–C11–C12	104.51(13)
C10–C11–C12	103.84(14)
O2–C11–H11	112.7
C10–C11–H11	112.7
C12–C11–H11	112.7
C15–C12–C11	117.13(15)
C15–C12–C6	117.91(15)
C11–C12–C6	105.71(12)
C15–C12–C8	118.19(13)
C11–C12–C8	107.43(13)
C6–C12–C8	85.81(12)
C3–C13–H13A	109.5
C3–C13–H13B	109.5
H13A–C13–H13B	109.5
C3–C13–H13C	109.5
H13A–C13–H13C	109.5
H13B–C13–H13C	109.5
C8–C14–H14A	109.5
C8–C14–H14B	109.5
H14A–C14–H14B	109.5
C8–C14–H14C	109.5
H14A–C14–H14C	109.5
H14B–C14–H14C	109.5
C12–C15–H15A	109.5
C12–C15–H15B	109.5
H15A–C15–H15B	109.5
C12–C15–H15C	109.5
H15A–C15–H15C	109.5
H15B–C15–H15C	109.5

Table 15. Torsion angles for Breit_IL_1637_iso_a

**Atom–Atom–Atom–Atom**	**Torsion Angle [°]**
C11–O2–C2–C3	163.46(13)
C11–O2–C2–C1	−67.82(15)
C11–O2–C2–C6	49.51(14)
O1–C1–C2–O2	−56.58(16)
C9–C1–C2–O2	64.22(16)
O1–C1–C2–C3	68.49(19)
C9–C1–C2–C3	−170.70(14)
O1–C1–C2–C6	−168.54(13)
C9–C1–C2–C6	−47.73(17)
O2–C2–C3–C13	35.6(2)
C1–C2–C3–C13	−88.2(2)
C6–C2–C3–C13	146.83(17)
O2–C2–C3–C4	−89.95(15)
C1–C2–C3–C4	146.28(14)
C6–C2–C3–C4	21.30(17)
C13–C3–C4–O3	44.1(2)
C2–C3–C4–O3	172.84(15)
C13–C3–C4–C5	−136.31(16)

C2–C3–C4–C5	−7.53(18)
O3–C4–C5–C6	170.29(16)
C3–C4–C5–C6	−9.33(18)
C4–C5–C6–C7	−108.80(17)
C4–C5–C6–C2	22.15(17)
C4–C5–C6–C12	135.83(15)
O2–C2–C6–C5	88.89(14)
C3–C2–C6–C5	−27.55(17)
C1–C2–C6–C5	−155.96(14)
O2–C2–C6–C7	−133.88(13)
C3–C2–C6–C7	109.68(15)
C1–C2–C6–C7	−18.72(18)
O2–C2–C6–C12	−40.86(14)
C3–C2–C6–C12	−157.30(13)
C1–C2–C6–C12	74.30(15)
C5–C6–C7–C8	−155.78(15)
C2–C6–C7–C8	76.00(14)
C12–C6–C7–C8	−24.17(12)
C6–C7–C8–C14	146.49(15)
C6–C7–C8–C9	−76.58(15)
C6–C7–C8–C12	23.83(11)
O1–C1–C9–C10	53.84(18)
C2–C1–C9–C10	−63.48(16)
O1–C1–C9–C8	164.46(14)
C2–C1–C9–C8	47.14(17)
C14–C8–C9–C10	−88.35(16)
C7–C8–C9–C10	132.57(14)
C12–C8–C9–C10	40.48(14)
C14–C8–C9–C1	157.80(15)
C7–C8–C9–C1	18.73(19)
C12–C8–C9–C1	−73.37(15)

C1–C9–C10–C11	64.15(16)
C8–C9–C10–C11	−50.93(15)
C2–O2–C11–C10	74.38(16)
C2–O2–C11–C12	−36.44(15)
C9–C10–C11–O2	−71.52(16)
C9–C10–C11–C12	39.75(15)
O2–C11–C12–C15	−124.00(15)
C10–C11–C12–C15	120.95(16)
O2–C11–C12–C6	9.66(16)
C10–C11–C12–C6	−105.39(14)
O2–C11–C12–C8	100.16(14)
C10–C11–C12–C8	−14.89(16)
C5–C6–C12–C15	35.2(2)
C7–C6–C12–C15	−95.97(16)
C2–C6–C12–C15	151.70(15)
C5–C6–C12–C11	−98.00(17)
C7–C6–C12–C11	130.79(13)
C2–C6–C12–C11	18.46(15)
C5–C6–C12–C8	155.05(15)
C7–C6–C12–C8	23.85(12)
C2–C6–C12–C8	−88.48(13)
C14–C8–C12–C15	−26.0(2)
C7–C8–C12–C15	95.91(16)
C9–C8–C12–C15	−150.96(15)
C14–C8–C12–C11	109.33(17)
C7–C8–C12–C11	−128.80(13)
C9–C8–C12–C11	−15.67(15)
C14–C8–C12–C6	−145.51(15)
C7–C8–C12–C6	−23.64(11)
C9–C8–C12–C6	89.49(12)

6 DFT-calculations on relative stability of 24a/ent-24b (exo/endo)

DFT Calculations

In order to understand the reaction outcome, we performed DFT calculations using Gaussian16.[18] All structures were optimized using the M06-2x functional[19] in combination with the def2SVP basis set.[20] All optimized structures showed no imaginary frequencies during vibrational analysis. All visualizations of calculated structures were obtained using cylview20.[21]

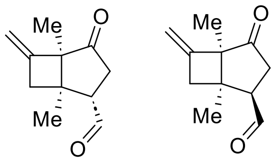

	exo	endo
ΔG/kcal/mol	0.00	-2.04

Relative stability both epimers (M06-2x/def2SVP).

Atomic Coordinates

Endo (**ent-24b**)

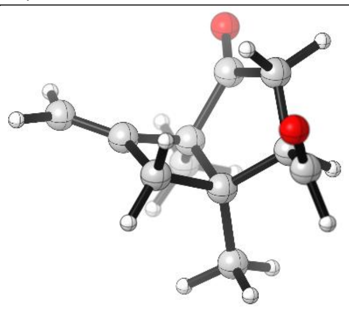	E/hartree	-577.315156
	E+zvp/hartree	-577.089121
	G/hartree	-577.128207

C	0.9338291	0.2547352	0.1724567
C	-0.4533529	-0.4648758	0.2946817
C	-1.5107619	0.6651812	0.4783567
C	-0.9121109	1.8902442	-0.2063793
H	-1.1407539	1.8646642	-1.2853413
H	-1.2608859	2.8557902	0.1798067
C	2.0280271	0.0723452	1.2057217
H	2.3370051	-0.9789978	1.2771097
H	2.8953191	0.6811022	0.9131737
H	1.7003471	0.4186652	2.1974267
C	-0.5454139	-1.5779228	1.3200867
H	-1.5127339	-2.0993258	1.2549727
H	0.2447681	-2.3244098	1.1513917
H	-0.4330109	-1.1878908	2.3428127
C	-0.3042219	-0.9598028	-1.1782073
H	-0.3950659	-2.0476608	-1.3142873
H	-0.9810089	-0.4646808	-1.8923853
C	1.1088191	-0.4174858	-1.1815023
C	2.1624091	-0.5395078	-1.9809883
H	3.1128081	-0.0628958	-1.7298843
H	2.1069301	-1.1160538	-2.9068793
C	0.5877981	1.7396192	-0.0162333
O	1.3908431	2.6290232	0.0232227
C	-2.8438209	0.2343152	-0.0798193
O	-3.2801819	0.6096262	-1.1339403
H	-3.4082629	-0.5109748	0.5322587
H	-1.6273179	0.8371732	1.5623697

Exo (**24a**)

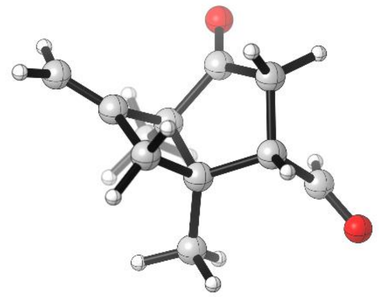	E/hartree	-577.311640
	E+zvp/hartree	-577.085773
	G/hartree	-577.124952

C	0.6471914	0.3150655	0.4241743
C	-0.4407536	-0.6370995	-0.1857547
C	-1.5093196	0.2708475	-0.8568177
C	-0.7434746	1.5452545	-1.2390387
H	-1.9836396	-0.2436685	-1.7050497
H	-0.2189066	1.4156915	-2.1993887
H	-1.3541626	2.4546205	-1.3173147
C	0.9274574	0.3767985	1.9121373
[18]H	1.2402914	-0.6028975	2.2972063
H	1.7286624	1.1065485	2.0959863
H	0.0400074	0.7125765	2.4697633
C	-1.0051966	-1.7065425	0.7308623
H	-1.7257456	-2.3368885	0.1875993
H	-0.1997026	-2.3515065	1.1113493
H	-1.5276176	-1.2738635	1.5968393
C	0.6172984	-1.1727385	-1.1974267
H	0.7610864	-2.2630295	-1.1754827
H	0.4447704	-0.8687605	-2.2416177
C	1.6734654	-0.3658725	-0.4719017
C	3.0001234	-0.3075685	-0.5022967
H	3.5460064	0.3553445	0.1731283
H	3.5787024	-0.9193205	-1.1979117
C	0.3105554	1.6976685	-0.1471667
O	0.7832984	2.7295305	0.2384233
C	-2.5953546	0.6027515	0.1385653
O	-3.7058076	0.1521645	0.1091233
H	-2.2892356	1.3148935	0.952010

7 References

[1] J. R. Frost, C. M. Pearson, T. N. Snaddon, R. A. Booth, R. M. Turner, J. Gold, D. M. Shaw, M. J. Gaunt, S. V. Ley, *Chemistry* **2015**, *21*, 13261.

[2] W. Jeong, M. J. Kim, H. Kim, S. Kim, D. Kim, K. J. Shin, *Angew. Chem., Int. Ed. Engl.* **2010**, *49*, 752.

[3] T. Sugihara, M. Yamaguchi, *Synlett* **1998**, *1998*, 1384.

[4] M. E. Jung, P. Koch, *Tetrahedron Lett.* **2011**, *52*, 6051.

[5] Y. Kaburagi, Y. Kishi, *Org. Lett.* **2007**, *9*, 723.

[6] *Org. Synth.* **1999**, *76*, 214.

[7] J. Pereira, N. Casaretto, G. Frison, B. Nay, *Chem. Sci.* **2025**, *16*, 11375.

[8] E. C. Gonzalez, I. M. de La Torre Roehl, B. M. Stoltz, *Chem. Sci.* **2025**, *16*, 11381.

[9] J. B. Martinez, P. R. Hanson, *J. Org. Chem.* **2025**, *90*, 14977.

[10] Y. Zhang, P. Yu, W. Chen, J. Li, K. Liu, X. Xie, H. Li, X. She, *Org. Lett.* **2025**, *27*, 5480.

[11] S.-Z. Huang, Q. Wang, J.-Z. Yuan, C.-H. Cai, H. Wang, A. Mándi, T. Kurtán, H.-F. Dai, Y.-X. Zhao, *J. Nat. Prod.* **2022**, *85*, 3.

[12] Bruker, SAINT, V8.42, Bruker AXS Inc., Madison, Wisconsin, USA.

[13] L. Krause, R. Herbst-Irmer, G. M. Sheldrick, D. Stalke, *J. Appl. Crystallogr.* **2015**, *48*, 3.

[14] G. M. Sheldrick, *Acta Crystallogr., Sect. A:Found. Adv.* **2015**, *71*, 3.

[15] G. M. Sheldrick, *Acta Crystallogr., Sect. C:Struct. Chem.* **2015**, *71*, 3.

[16] C. R. Groom, I. J. Bruno, M. P. Lightfoot, S. C. Ward, *Acta Crystallogr., Sect. B:Struct. Sci., Cryst. Eng. Mater.* **2016**, *72*, 171.

[17] D. Kratzert, FinalCif, V143, https://dkratzert.de/finalcif.html.

[18] Gaussian 16, Revision C.01, M. J. Frisch, G. W. Trucks, H. B. Schlegel, G. E. Scuseria, M. A. Robb, J. R. Cheeseman, G. Scalmani, V. Barone, G. A. Petersson, H. Nakatsuji, X. Li, M. Caricato, A. V. Marenich, J. Bloino, B. G. Janesko, R. Gomperts, B. Mennucci, H. P. Hratchian, J. V. Ortiz, A. F. Izmaylov, J. L. Sonnenberg, D. Williams-Young, F. Ding, F. Lipparini, F. Egidi, J. Goings, B. Peng, A. Petrone, T. Henderson, D. Ranasinghe, V. G. Zakrzewski, J. Gao, N. Rega, G. Zheng, W. Liang, M. Hada, M. Ehara, K. Toyota, R. Fukuda, J. Hasegawa, M. Ishida, T. Nakajima, Y. Honda, O. Kitao, H. Nakai, T. Vreven, K. Throssell, J. A. Montgomery, Jr., J. E. Peralta, F. Ogliaro, M. J. Bearpark, J. J. Heyd, E. N. Brothers, K. N. Kudin, V. N. Staroverov, T. A. Keith, R. Kobayashi, J. Normand, K. Raghavachari, A. P. Rendell, J. C. Burant, S. S. Iyengar, J. Tomasi, M. Cossi, J. M. Millam, M. Klene, C. Adamo, R. Cammi, J. W. Ochterski, R. L. Martin, K. Morokuma, O. Farkas, J. B. Foresman, and D. J. Fox, Gaussian, Inc., Wallingford CT, 2016.

[19] Y. Zhao, D. G. Truhlar, *Theor. Chem. Account.* **2008**, *120*, 215.

[20] F. Weigend, R. Ahlrichs, *Phys. Chem.Chem. Phys.* **2005**, *7*, 3297.

[21] CYLview20, Legault, C. Y., Université de Sherbrooke, 2020 (http://www.cylview.org).
